# Self-immolative nanocapsules precisely regulate depressive neuronal microenvironment for synergistic antidepression therapy

**DOI:** 10.1186/s12951-023-02008-9

**Published:** 2023-08-17

**Authors:** Ziyao Liu, Bei Chen, Shijun Xiang, Shuo Hu

**Affiliations:** 1 Department of Nuclear Medicine, Xiangya Hospital, Central South University, Changsha, 410008 China; 2 National Clinical Research Center for Geriatric Diseases, Xiangya Hospital, Central South University, Changsha, 410008 China; 3 Key Laboratory of Biological, Nanotechnology of National Health Commission, Xiangya Hospital, Central South University, Changsha, 410008 China

**Keywords:** Nanoparticle degradation, Protein-loaded nanocapsules, Neurotransmitter delivery, Self-immolative nanocapsules, Synergistic antidepression therapy

## Abstract

**Background:**

Pharmacotherapy constitutes the first-line treatment for depression. However, its clinical use is hindered by several limitations, such as time lag, side effects, and narrow therapeutic windows. Nanotechnology can be employed to shorten the onset time by ensuring permeation across the blood brain barrier (BBB) to precisely deliver more therapeutic agents; unfortunately, formidable challenges owing to the intrinsic shortcomings of commercial drugs remain.

**Results:**

Based on the extraordinary capability of monoamines to regulate the neuronal environment, we engineer a network nanocapsule for delivering serotonin (5-hydroxytryptamine, 5-HT) and catalase (CAT) to the brain parenchyma for synergistic antidepression therapy. The nanoantidepressants are fabricated by the formation of 5-HT polymerization and simultaneous payload CAT, following by surface modifications using human serum albumin and rabies virus glycoprotein. The virus-inspired nanocapsules benefit from the surface-modifying strategies and exhibit pronounced BBB penetration. Once nanocapsules reach the brain parenchyma, the mildly acidic conditions trigger the release of 5-HT from the sacrificial nanocapsule. Releasing 5-HT further positively regulate moods, relieving depressive symptoms. Meanwhile, cargo CAT alleviates neuroinflammation and enhances therapeutic efficacy of 5-HT.

**Conclusion:**

Altogether, the results offer detailed information encouraging the rational designing of nanoantidepressants and highlighting the potential of nanotechnology in mental health disorder therapies.

**Graphical abstract:**

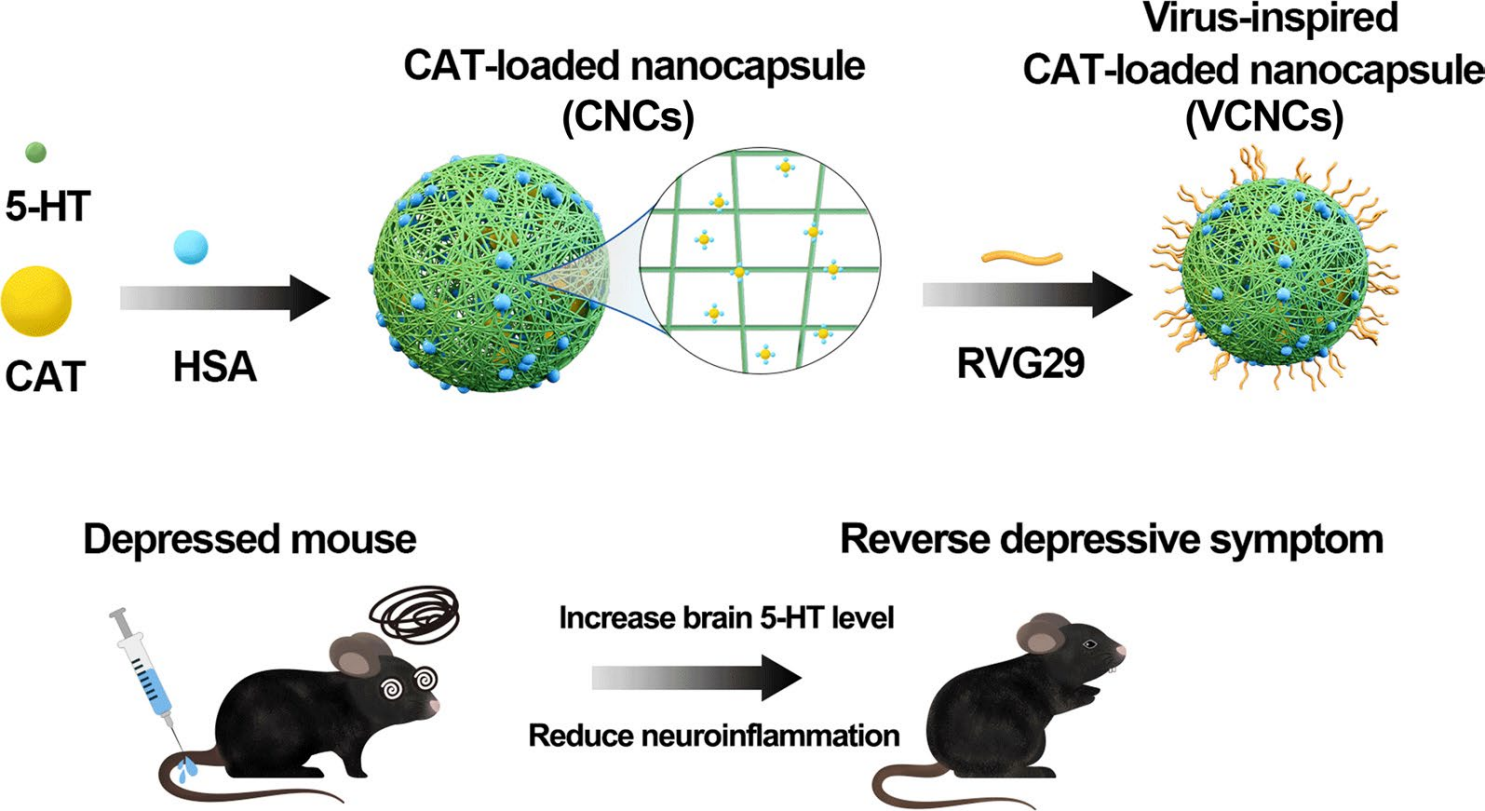

**Supplementary Information:**

The online version contains supplementary material available at 10.1186/s12951-023-02008-9.

## Introduction

As faster pace of life causally involved in the mental stress, depression is a common mental health disorder worldwide, and more than 300 million people are suffering from depression all over the world according to the World Health Organization [1]. Major depressive disorder is even the leading cause of disability and the major contributor to the suicide burden [2, 3], hence progressive attentions are paid to depressive disorder treatment. The antidepressant agents currently used are designed to raise monoamine levels in the brain [4, 5]. It is reported that the depression is caused by decreased monoamine levels; this hypothesis has been widely supported by the effects of pharmaceuticals agents that increase the levels of monoamine neurotransmitters [6]. Monoamine neurotransmitters, including serotonin, dopamine, and norepinephrine, synergistically regulate the different aspects of emotional, rewards, and general behaviors [7, 8]. Among commercial drugs, fluoxetine is the first choice in pharmacological treatment and it selectively inhibits serotonin reuptake [9]. However, the current pharmacotherapies takes weeks to months to achieve full efficacy [10], and is ineffective in approximately 30%–50% of patients or causes intolerable side effects [11]. This disappointing therapeutic outcome may reflect the complicated mechanism of depression. The actual etiology of depression is yet to be clarified, which constitute multiple alterations in the brain comprising the dysregulation of neurotransmitters [12], pathologic increase in inflammatory markers [13], and decrements in neurotrophic factors [14]. Taken together, depression is complex and multifaceted, thereby the dilemma of monotherapy that targets monoamine prompting the need for novel and synergistic therapeutic strategies.

To achieve rapid antidepressant action, nanocarriers were adopted to precisely deliver fluoxetine to the brain parenchyma [15, 16]. Normally, it requires two weeks to produce a therapeutic response, reducing the delayed onset of the response. However, the low rate of responsiveness and non-negligible adverse effects of fluoxetine limit its broader use and highlight the need for combined therapies. Some studies adopted nanoantidepressants combined with photothermal therapy to broaden the antidepressant spectrum and enhance its efficacy [15–17]. Still, the uncertain biosafety of increased brain temperature due to the photothermal procedures may pose unknown risks to patients. Furthermore, these strategies are unable to directly remodel the neuronal microenvironment. Inspired by neuroregulation capability of the neurotransmitters, delivering high amounts of monoamines to the brain parenchyma could be a safer and faster way to achieve antidepression. Notwithstanding, delivering monoamines into the brain is challenging owing to their intrinsic properties, including polarity and nonspecific distribution, which prevent monoamines from permeating the blood brain barrier (BBB). The BBB maintains the homeostasis of the central nervous system (CNS) by tightly controlling the passage of substances in and out, thereby restricting the entry of nearly all macromolecules and > 98% of small molecule drugs [18]. In addition, the high degradability and clearance rate of most monoamines hinder their therapeutic effect in vivo. Hence, the development of a robust carrier system for monoamine delivery provides a new insight for the advancement of antidepressant agents.

**Scheme 1:**
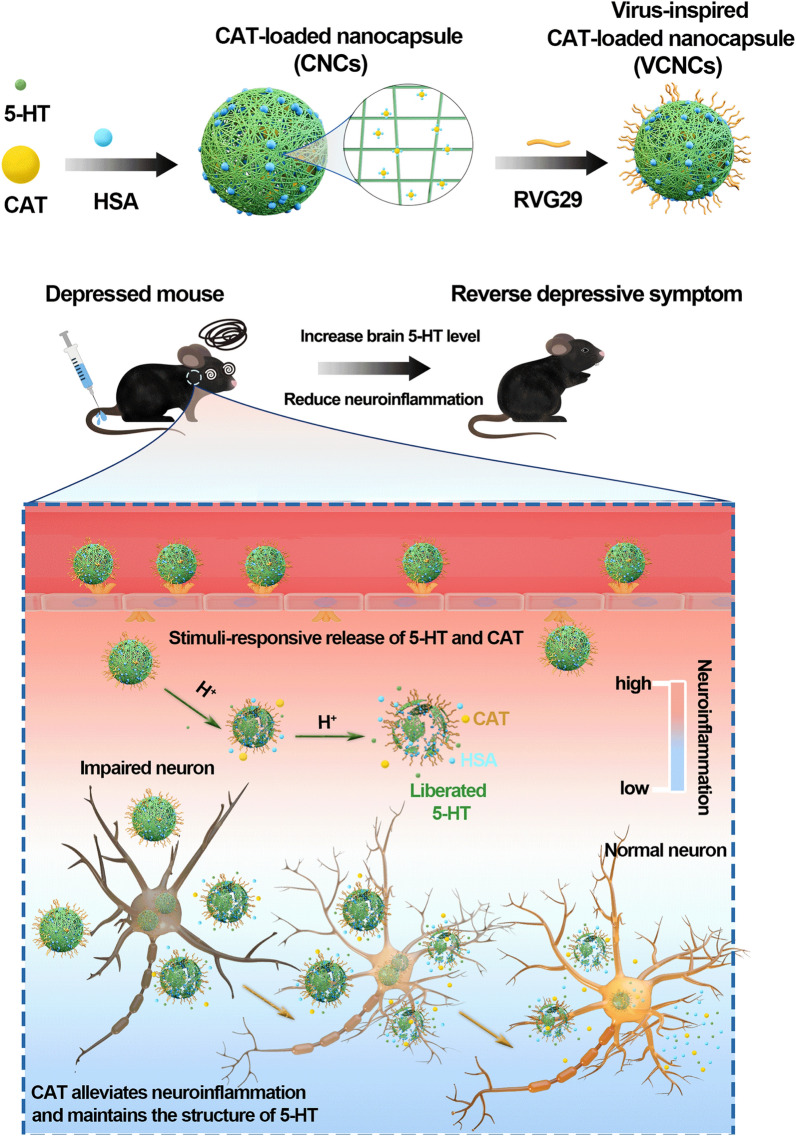
Schematic illustration of nanocapsules for synergistic antidepression therapy

Our study introduced rabies-virus inspired network nanocapsules to achieve the brain delivery of serotonin (5-hydroxytryptamine, 5-HT) and catalase (CAT) for antidepression therapy. These nanocapsules were surface-modified using rabies virus glycoprotein (RVG29), which enabled them to permeate the BBB and enter the brain parenchyma, presumably via specific recognition by nicotinic acetylcholine receptors (nAchR) [19, 20]. Furthermore, surface modification with human serum albumin (HSA) prolonged the blood circulation time of the nanocapsules [21, 22]. Several publications have indicated that the pH of the brain in patients with depression falls within the range of 6.2 to 6.4 [23–25]. Once the nanoantidepressant reaches the depressive neuronal microenvironment, 5-HT is gradually released from the self-immolative nanoformulation following degradation due to the mildly acidic microenvironment (Scheme 1). This nanoantidepressant with stimuli-responsive release can be used for synergistic dual-action therapy with the aim of (i) precisely supplementing with monoamine neurotransmitters as 5-HT could potently alleviate depressive symptoms and (ii) positively regulating the neuronal microenvironment via CAT as 5-HT is easily oxidized under inflammatory conditions. The alleviation of inflammation via CAT can in turn enhance the efficacy of 5-HT. The network nanoantidepressant constituting active molecules exhibits stable delivery into the blood, high BBB penetrance, and precise controllability of drug release. Consequently, the multitargeting nanosystem is expected to positively regulate the depressive neuronal microenvironment in a highly efficient and precise manner.

## Materials and methods

### Synthesis protocols of network nanocapsules with different proteins

The synthesis protocol of network nanocapsules with different proteins was carried out by varying the pH to achieve optimal conditions. The aqueous solution of 5-HT and CAT/HSA samples were added to 21 mL Tris–HCl buffer (pH 9.5 or pH 7.4) to obtain inner-core nanoparticles (NPs). To obtain CAT-loaded nanocapsules (CNCs), the above inner NPs were incubated with HSA at a mass ratio of 1:1 in PBS for 1 h. Unabsorbed HSA was removed by membrane dialysis (100 kDa) in ultrapure water at pH 7.4 for 6 h. The size distribution of hydrodynamic diameters N(d_h_) of NPs from different pH buffers was characterized by dynamic light scattering (DLS) using a UV-cuvette (ZH 8.5 mm; Deckel; Sarstedt, Germany). The distribution of ζ-potential I(ζ) of these NPs was also recorded by DLS with laser Doppler anemometry (LDA). The optimal pH value was found to be 9.5. After performing the optimized synthesis protocol, the load capacity of different proteins was tested. Four different proteins were chosen, namely bovine serum albumin (BSA, Aladdin, #A104912, China), catalase (CAT, Aladdin, #C128526, China), α-chymotrypsin (α-CT, Sigma-Aldrich, #C4129, Germany), and lysozyme (Lys, Aladdin, #L105521, China), each with different molecular weights and isoelectric points. Nanocapsules loaded with proteins were constructed using the above protocol. Since the synthetic procedures contained two proteins (HSA and cargo proteins), the cargo proteins were conjugated with fluorescein5(6)-isothiocyanate (FITC, Aladdin, #F106837, China) to quantify the loading efficiencies. The samples were purified and concentrated by lyophilization. The labeling efficiency was calculated using Beer-Lambert’s law through ultraviolet visible spectrum (UV–vis, UV-2450, Shimadzu, Japan). Furthermore, the nanocapsules loaded with fluorescent proteins were synthesized using the same methods. The fluorescence intensity I_FITC_ at 520 nm emission wavelength under excitation at 488 nm of proteins-FITC was recorded by a fluorescence meter (HORiBA Fluoromax-4, Horiba Scientific, Japan) to quantify the loading efficiencies. To achieve brain targeting ability, rabies virus glycoprotein (RVG29) was used for the surface functionalization of NPs. CNCs were incubated with RVG29 at a mass ratio of 4 eq of CNCs per 1 eq of RVG29 in PBS for 1 h to obtain virus-inspired CAT-loaded nanocapsules (VCNCs). Unabsorbed RVG29 was removed by membrane dialysis (5 kDa) in ultrapure water at pH 7.4 for 4 h. For the control group (virus-inspired nanocapsules (VNCs)), only HSA (1500 μL) was used for synthesis. The hydrodynamic diameters d_h(N)_ and ζ-potential values of the samples during the synthetic procedures were measured by DLS and LDA, respectively. The shape of VCNCs and CNCs was determined using transmission electron microscopy (TEM, Talo F200S SUPERX, Thermo Scientific, USA). To obtain TEM images, a droplet of the sample was placed on top of a copper grid. The synthesis protocol for the inner core NPs and the FITC labeling protocol are detailed in the supporting information section.

### Structural characterizations of 5-HT covalent bonds

To confirm the formation of 5-HT covalent bonds, the structure was characterized using UV–vis spectra and Fourier Transform Infra-Red (FTIR, Nicolet is 50, Thermo Scientific, USA). Additionally, CAT, HSA, 5-HT, VCNCs, CNCs, and VNCs at a concentration of C = 1 mg/mL were characterized using UV–vis spectroscopy and FTIR spectroscopy.

### Characterizations of VCNCs, CNCs, and VNCs

The morphology of the nanocapsules was characterized using DLS and TEM. The colloidal stability of the nanocapsules was studied using DLS in PBS. Specifically, 1 mg of nanocapsules was mixed with 1 mL of PBS, and the hydrodynamic diameter d_h(N)_ and ζ-potential were measured using DLS over a total period of 12 d. The enzyme stability of CAT in CNCs and VCNCs was evaluated in PBS over a period of 5 d. For this, VCNCs, CNCs, and free CAT were dissolved in 1 mL of PBS at a concentration of C_CAT_ = 10 μg/mL each and were kept at room temperature for 5 d. The CAT viability was measured at t = 0 h as a control using a testing kit (Solarbio, #BC0200, China). After the specific incubation times, the data were measured and normalized to V = 100% at t = 0 h. The data are presented as viability percentages. To explore the anti-adsorption properties of unspecific proteins, the interaction between the nanocapsules and bovine serum albumin (BSA) was investigated. Briefly, 0.5 mL of VCNCs, CNCs, and VNCs at a concentration of C_NPs_ = 1 mg/mL was incubated with 0.5 mL of BSA at a concentration of C_BSA_ = 1 mg/mL on a shaker for 24 h. At specific time points, the free proteins were separated using an ultracentrifuge filter (cutoff molecular weight 100 kDa) at 2000 rpm for 10 min. The eluents were collected and tested using BCA assays according to the instructions (Beyotime, #P0010, China). The data were normalized using the sham group (BSA alone). To quantify the 5-HT concentration in the nanocapsules, the UV–vis spectra of different dilutions of 5-HT were measured, and the UV–vis absorbance at 204 nm versus the 5-HT concentration was fitted with a linear function. The absorbances of VCNCs, CNCs, and VNCs at 204 nm were extracted to calculate the concentration of 5-HT. Next, the protein concentrations in the nanocapsules were measured using a BCA assay (Beyotime, #P0010, China), and the CAT concentration was measured using a testing kit (Solarbio, #BC0200, China) according to the instructions.

### Stimuli-responsive release of 5-HT

To simulate the real environment, we tested the release of 5-HT in PBS buffer (pH 7.4), citric acid-Na_2_HPO_4_ buffer (pH 6.4), pH 7.4 + ROSUP buffer, and pH 6.4 + ROSUP buffer (1:1000, Beyotime, #S0033S, China). Initially, we characterized the morphology changes of nanocapsules in acidic and inflammatory conditions. We immersed 1 mg of VCNCs, CNCs, and VNCs respectively in 1 mL of pH 6.4 buffer (Citric acid-Na_2_HPO_4_ buffer), pH 7.4 + ROSUP buffer, and pH 6.4 + ROSUP buffer for 8 d. At specific time points, we tested the mean hydrodynamic diameter d_h(N)_ by dynamic light scattering (DLS). We also measured the nanocapsule degradation using high-performance liquid chromatography (HPLC, Agilent 1260 Infinity, Agilent Technologies, USA) and ESI. VCNCs, CNCs, and VNCs at a concentration of C_5-HT_ = 5000 μg/mL were dissolved in different media, namely pH 7.4 (PBS), pH 6.4 buffer, pH 7.4 + ROSUP buffer (1:1000), and pH 6.4 + ROSUP buffer (1:1000) for 72 h. The mixture was then placed in an ultracentrifuge tube with a 3 kDa cutoff and centrifuged at 8000 rpm for 30 min. The remaining NPs and proteins were retained in the filters, while the liberated 5-HT monomers were eluted. We collected the eluate and analyzed it by HPLC. Next, we collected the separated samples at retention time from 2 to 4 min and tested them by ESI–MS. To quantify the cumulative release profile of 5-HT, we measured the eluate by HPLC at different time points. To demonstrate that CAT protects the structure of 5-HT under oxidative conditions, we incubated 150 μg/mL of 5-HT with CAT (2 μg/mL) for 1 h in pH 7.4 + ROSUP buffer. The control groups represented the same concentration of 5-HT and CAT, respectively, in pH 7.4 + ROSUP buffer for 1 h. Additionally, 150 μg/mL of 5-HT was incubated in ultrapure water for 1 h. After incubation, samples were centrifuged with ultracentrifuge tubes with a cutoff molecular weight of 3 kDa at 8000 rpm for 30 min. The eluent was characterized by UV–Vis spectra. The methods of HPLC are shown in the supporting information. To comprehensively investigate the polymerization of 5-HT in this study, we synthesized poly(5-HT) using the same method without the presence of proteins. To obtain the degraded poly(5-HT), the powder of poly(5-HT) was incubated with a pH 6.4 buffer (Citric acid-Na_2_HPO_4_ buffer) for 72 h. The structure of samples was measured using FTIR (Nicolet is 50, Thermo Scientific, USA), X-Ray Photoelectron Spectroscopy (XPS, ESCALAB Xi + , ThermoFischer, USA), Electrospray Ionization Mass Spectroscopy (ESI–MS, LTQ Orbitrap Velos Pro, Thermo Scientific, USA). The detailed information of methods are provided in suppporting information.

### Fluorescence labeling

Human serum albumin (HSA, Aladdin, #H304436, China) was labeled with Cy5.5 NHS (Aladdin, #C171354, China) and Cy7.5 NHS (Aladdin, #C171364, China), and rabies virus glycoprotein (RVG29, Glbiochem, China) was labeled with FITC (Aladdin, #F106387, China). The labeling process is described in detail in the supporting information. HSA-Cy5.5 or HSA-Cy7.5 and RVG29-FITC were used to construct fluorescence-labeled nanocapsules using the method described above.

### Cell cultures and cytotoxicity assays

Mouse brain cell line bEnd.3 cells and rat pheochromocytoma cell line PC-12 cells were cultured in Dulbecco’s modified Eagle’s medium (DMEM, Gibco, USA) supplemented with 10% fetal bovine serum (Excell, China) and 100U/mL penicillin/streptomycin (Gibco, USA) at 37 °C and 5% CO_2_.

The resazurin assay was used to evaluate the cell viability of bEnd.3 and PC-12 cells after exposure to VCNCs, CNCs, VNCs, CAT, 5-HT, and HSA. PC-12 and bEnd.3 cells were seeded in 96-well plates with 0.32 cm^2^ growth area per well at a density of 30,000 cells in 150 μL medium per well. After overnight growth, the cells were exposed to different samples for 24 and 48 h. The concentration ranges of all the samples are shown in Additional file 1: Table S4. After incubation, the cells were washed once with 150 μL of PBS. Then, 100 μL of 10% resazurin solution (0.25 mg/mL, Aladdin, #R105538, China) in complete cell media was added to the cells and incubated for 4 h. The OD value of each well was measured at 570 nm and 600 nm using a microplate reader (Epoch, BioTek, USA). The OD value is positively related to the number of living cells. The OD values of cells incubated with test samples were normalized to the cell medium containing resazurin which had not been exposed to cells and samples. The cell viability was calculated using Eq. 1 and Additional file 1: Table S4.

1 $$\% \,{\text{Reduction of resazurin agent}}\, = \,\left( {{{\text{E}}_{{\text{oxi}}}}600\, \times \,{\text{A}}570} \right) - \left( {{{\text{E}}_{{\text{oxi}}}}570\, \times \,{\text{A}}600} \right)/\left( {{{\text{E}}_{{\text{red}}}}570\, \times \,{\text{C}}600} \right) - \left( {{{\text{E}}_{{\text{red}}}}600\, \times \,{\text{C}}570} \right)$$

Where E_oxi_570 = molar extinction coefficient of oxidized resazurin agent at 570 nm = 80,586; E_oxi_600 = molar extinction coefficient of oxidized resazurin agent at 600 nm = 117,216; E_red_570 = molar extinction coefficient of reduced resazurin agent at 570 nm = 155,677; E_red_600 = molar extinction coefficient of reduced resazurin agent at 600 nm = 14,652; A570 = absorbance of samples at 570 nm; A600 = absorbance of samples at 600 nm; C570 = absorbance of negative controls (cell medium with resazurin agent) at 570 nm; C600 = absorbance of negative controls (cell medium with resazurin agent) at 600 nm.

### Endocytosis studies using flow cytometry

In this study, we employed flow cytometry (BD LSR Forteassa Biosciences, USA) to analyze the cellular uptake of VCNCs-Cy5.5, CNCs-Cy5.5, and VNCs-Cy5.5. Initially, bEnd.3 cells were seeded at a density of 150,000 cells/mL in a volume of 1 mL into 24-well plates (growth area = 1.9 cm^2^) and were allowed to incubate overnight. Subsequently, the cells were incubated with fresh cell medium containing Cy5.5 labeled nanocapsules at a concentration of C_5-HT_ = 25 μg/mL for 1, 3, and 5 h. Time-dependent endocytosis of nanocapsules was monitored by characterizing the cellular fluorescence over time using flow cytometry. At specific time points, the supernatant was removed, and cells were washed twice with 1 mL of PBS. The cells were then detached by adding 0.1 mL of 0.05% trypsin/EDTA solution (Procell, China) and neutralized with 0.4 mL of complete cell medium. We collected the cells by centrifugation at 400*g* for 8 min, and the obtained cell pellet was resuspended in 0.2 mL of PBS and analyzed using flow cytometry (BD LSR Forteassa Biosciences, USA). For each experiment, 20,000 gated cells were counted and analyzed.

### In vitro model for detecting the penetration efficiency of nanocapsules

PC-12 cells and bEnd.3 cells were used to construct an in vitro blood–brain barrier (BBB) penetration model. Briefly, bEnd.3 cells were seeded onto transwell filters (pore size 0.4 μm, 6.5 mm, Costar, USA) at a density of 80,000 cells/well. Meanwhile, PC-12 cells were seeded into 24-well plates (surface area 1.9 cm^2^) with 120,000 cells per well in 1 mL of complete cell medium. It's important to note that bEnd.3 cells and PC-12 cells were not in the same well. The next day, the cells were exposed to 0.3 mL of VCNCs-Cy5.5, CNCs-Cy5.5, and VNCs-Cy5.5 (C_5-HT_ = 25 μg/mL) in complete cell medium for an exposure time of t_exp_ = 3 h. After that, one part was used for the analysis of the uptake study, characterized by flow cytometry. The other part was used for further study. The supernatant was replaced by washing with PBS twice, and the cells were further incubated with fresh medium. The insert filters containing bEnd.3 cells were further coincubated with PC-12 cells at the lower chamber for another incubation time of t_inc_ = 24 h to characterize the penetration of nanocapsules from the apical chamber to the basolateral chamber. The intracellular fluorescence of collected cells in the apical chamber and basolateral chamber was analyzed by flow cytometry to calculate the penetration efficiency at t_exp_ + t_inc_ = 3 h + 24 h. The intracellular fluorescence per cell decreases over time due to proliferation. Cell proliferation needs to be taken into account when studying permeability efficiency. Therefore, the number of cells in every testing sample was counted. The normalized penetration efficiencies were calculated from the data in Additional file 1: Fig. S28 and Fig. 3A, multiplied by the growth factor in Additional file 1: Fig. S29. The penetration efficiency was also visualized by confocal laser scanning microscopy (CLSM; LSM900, Zeiss, Germany). At t_exp_ + t_inc_ = 3 h + 24 h, the PC-12 cells and bEnd.3 cells were fixed with 4% paraformaldehyde (100 μL/well), washed twice with PBS, and stained with DAPI (Beyotime, #C1006, China) at 37 °C. The images of the samples were finally captured under CLSM. To further study the integrity of nanocapsules after passing through bEnd.3 cells, dual-labeled nanocapsules (HSA-Cy5.5, RVG29-FITC) were adopted to establish the transwell system. The methods were the same as the ones using Cy5.5 labeled nanocapsules.

### Cellular anti-inflammation of nanocapsules after passing through bEnd.3 cells

To assess the anti-inflammatory properties of nanocapsules after passing through bEnd.3 cells, we investigated their ability to scavenge ROS. 2ʹ,7ʹ –dichlorofluorescein diacetate (DCFH-DA) is commonly used to detect intracellular ROS levels. To induce intracellular ROS production, we added the ROSUP reagent (1:1000, Beyotime, #S0033S, China) to PC-12 cells in serum-supplemented medium for 0.5 h. The cells were pre-incubated with bEnd.3 cells containing nanocapsules in the transwell filter for t_exp_ + t_inc_ = 3 h + 24 h. The PC-12 cells were then washed twice with PBS and incubated with 0.5 mL of 0.1% DCFH-DA (Beyotime, #S0033S, China) diluted in serum-free DMEM medium for 0.5 h at 37 °C. The cells were then washed twice with PBS and exposed to 0.5 mL of FBS-free DMEM medium. The fluorescence of DCFH was measured using a microplate reader (Envision@2015, PerkinElmer, USA) and an inverted fluorescence microscope (Eclipse Ti2, Nikon, Japan) at excitation and emission wavelengths of 488 nm and 520 nm, respectively. To investigate whether phenolic hydroxyl groups in 5-HT polymerization influences ROS concentration, we performed additional experiments using the same methods as described above. Further details of these experiments are provided in the supporting information.

### Cytoplasmic calcium concentration assay

To assess changes in cytoplasmic calcium concentration, we used Fluo-4 AM (Beyotime, #S1060, China). We seeded 150,000 PC-12 cells in 1 mL of cell culture medium with 10% FBS in a 24-well plate (growth area 1.9 cm^2^) and incubated them overnight. The cells were then exposed to different concentrations of VCNCs, CNCs, VNCs, 5-HT, CAT, and HSA for 3 h, as specified in Additional file 1: Table S5. After exposure, we washed the PC-12 cells twice with 1 mL of PBS and stained them in 0.5 mL of FBS-free DMEM medium containing 0.2 μM Fluo-4 AM for 0.5 h. The cells were then washed twice with PBS and kept stained for another 0.5 h. The fluorescence of Fluo-4 was detected using a microplate reader (Envision@2015, PerkinElmer, USA) at excitation and emission wavelengths of 488 nm and 520 nm, respectively.

### Cellular release of 5-HT monomers from nanocapsules

To investigate the cellular release of 5-HT monomers from nanocapsules, PC-12 cells were seeded in 24-well plates with a surface area of 1.9 cm^2^ at a density of 150,000 cells per well in a volume of 1 mL. The following day, pH 6.4 medium was prepared by mixing 1 M HCl and serum-supplemented medium to obtain the medium at pH 6.4. The ROSUP medium was prepared by diluting 1 μL of ROSUP reagent (Beyotime, #S0033S, China) with 1 mL of complete DMEM medium. Cells were incubated with VCNCs, CNCs, and VNCs at a dose of C_5-HT_ = 25 μg/mL in complete DMEM media, pH 6.4 media, ROSUP media (1:1000), and pH 6.4 + ROSUP media for 3 h. After incubation, the supernatant above the cells was collected, and the cells were washed twice with PBS. The cells were detached from the 24-well plate using 0.05% trypsin/EDTA in a volume of 0.1 mL. After aspirating the trypsin, 0.3 mL of medium was added to collect the cell pellets, which were then centrifuged at 400*g* for 8 min and washed twice with PBS to obtain the cell pellet. Each sample was added to 0.1 mL of PBS, and a handheld homogenizer was used to crumb the cell samples. The supernatant and cell samples were then analyzed using a 5-HT Elisa Kit (Elabscience, #E-EL-0033c, China), following the manufacturer's instructions**.**

### Behavioral tests and chronic unpredictable mild stress (CUMS) model

Male C57bl/6j mice (3–4 w) were obtained from Hunan SJA laboratory animal Co., Ltd (Changsha, China) and were housed at a constant temperature of 21 °C and 50% humidity. All procedures were approved by the Committee on Experimental Animal at XiangYa Hospital, Central South University (grant number: 2022020460). After one week of habituation, behavioral tests were used to select mice with normal locomotion. Subsequently, the mice were subjected to different and repeated unpredictable stressors for five weeks to establish the CUMS model. The CUMS-induced depressed mice were exposed to various mild stressors that changed from day to day to establish an unpredictable procedure. The stressors involved changes in the environment (reversed light/dark cycle, restraint, cage tilting, strobe light, wet bedding), social stressors (crowding), fear stressors (cold swim, tail pinch), and water/food deprivation. A detailed account of the procedures in the five weeks is provided in Additional file 1: Table S6. Behavioral tests were used to confirm the establishment of the CUMS depression model. Subsequently, the mice were randomly divided into six groups (n = 6): 0.9% saline (CUMS + saline), 400 μg/kg fluoxetine (CUMS + Flu), C_5-HT_: 400 μg/kg VCNCs (CUMS + VCNCs), C_5-HT_: 400 μg/kg CNCs (CUMS + CNCs), C_5-HT_: 400 μg/kg VNCs (CUMS + VNCs) via intravenous injection at 2-day intervals for two weeks. To maintain the depressed state, the mice continued to be subjected to stressors during the treatment session (Additional file 1: Table S7). Finally, the therapeutic outcomes were measured using three behavioral tests: the sucrose preference test (SPT), open field test (OFT), and forced swim test (FST). The results were analyzed using Smart V03 software. The details of the behavioral tests are provided in the supporting information.

### Blood collections and measurements

After the third-session behavioral tests, blood was drawn from the mice's eyeball. One part of the blood was collected in heparin anticoagulant tubes, and the blood panel parameters were measured using an Automatic Analyzer. Another part of the blood was left undisturbed at room temperature for 30 min to allow it to clot. The samples were then centrifuged at 4000*g* for 10 min at 4 °C to remove the clot and collect the serum. The alanine aminotransferase (ALT), aspartate aminotransferase (AST), and blood urea nitrogen (BUN) in the serum were analyzed using an Automatic Analyzer. The serum cortisol level was characterized using the ELISA kit (Elabscience, #E-EL-0161c, China) according to the manufacturer’s instructions.

### In vivo brain targeting ability

To investigate the brain targeting ability of the nanocapsules, we intravenously injected Cy7.5-labeled nanocapsules at a dose of C_5-HT_: 400 μg/kg into male C57bl/6j mice (20–25 g) and CUMS mice for 3 h. To minimize interference from fur, a large portion of the mice's fur was removed before injection. We used the IVIS Lumina II (Perkin Elmer, USA) to observe the in vivo fluorescence of Cy7.5. Subsequently, we harvested the brains for ex vivo fluorescence imaging. To further validate our findings, we conducted a similar study on nude mice (4 w) that received intravenous administration of Cy5.5-labeled nanocapsules at C_5-HT_: 400 μg/kg. After 3 h of injection, we used the IVIS Lumina II to observe the in vivo fluorescence.

### The measurements of biochemical factors in the hippocampus

For the enzyme-linked immunosorbent assay (ELISA) study, the dissected hippocampus was added to PBS and homogenized for 1 min. The sample solutions were then measured using IL-6 (Elabscience, #E-MSEL-M0001, China), IL-1β (Elabscience, #E-EL-M0037c, China), TNF-α (Elabscience, #E-EL-M3063, China), BDNF (Elabscience, #E-EL-M0203c, China), Nrf2 (Cusabio, #CSB-E16188m, China), and 5-HT ELISA kits (Elabscience, #E-EL-0033c China) according to the manufacturer’s instructions. Furthermore, the protein concentration of samples were analyzed by BCA anssys (Beyotime, #P0010, China). The biochemical factors were normalized by the protein concentrations. For reverse transcription quantitative polymerase chain reaction (RT-qPCR), the total RNA of the hippocampus was extracted with TrizolTM Reagent (Life Technologies, Carlsbad, USA), and PCR amplification was performed using GoScriptTM Reverse Transcription System (Promega, #A5000, USA). The sequences of primers are provided as follows. The RT-qPCR was analyzed using GoTaq@ qPCR Master Mix (A6001, Promega, USA). The relative mRNA expression of target genes was monitored by QuantStudio 6 Flex (Applied Biosystems, Life Technologies, USA) with the 2^−△△Ct^ method. Endogenous GAPDH was adopted as the negative control. The primer pairs were as follows: GAPDH, forward 5ʹ-GCCAAGGTCATCCATGACAACT-3ʹ, reverse 5ʹ-GAGGGGCCATCCACAGTCTT -3ʹ; NLRP3, forward 5ʹ-CTCGCATTGGTTCTGAGCTC-3ʹ, reverse 5ʹ-AGTAAGGCCGGAATTCACCA-3ʹ; BDNF, forward 5′-CAGGGGCATAGACAAAAG-3ʹ, reverse 5ʹ-GAGGGGCCATCCACAGTCTT-3ʹ; Nrf2, forward 5ʹ-CTATAGGTCCCGTTCGCTGAG-3ʹ, reverse 5ʹ-AAGAGACCTCCGTCGGT ACT-3ʹ; Iba 1, forward 5ʹ-CAGACTGCCAGCCTAAGACA-3ʹ, reverse 5ʹ- AGGAATTGCTTGTTGATCCC -3ʹ; GFAP, forward 5ʹ- CCTTCTGACACGGATTTGGT-3ʹ, reverse 5ʹ- TAAGCTAGCCCTGGACATCG-3ʹ. The expressions of Nrf2 and BDNF were detected by western blot (WB) analysis. The methods of WB are povided in the supporting information.

### Histological assays

After blood collection, one part of the mice was perfused with PBS and 4% paraformaldehyde, and the brains were harvested, embedded in paraffin and OCT compound, and sectioned into 4 μm and 9 μm thickness, respectively. Other parts of the mice were sacrificed after blood collection, and major organs (heart, liver, spleen, lung, and kidney) and brain were harvested. The hippocampal regions were dissected from the brain on ice and stored at − 80 °C. The slices were processed with ROS fluorescent staining, immunofluorescence staining, immunohistochemistry (IHC), Nissl, and Hematoxylin–eosin staining (HE). Details are provided in the supporting information.

### mRNA sequencing analysis

Total RNA was extracted from the hippocampus of mice brains using TrizolTM Reagent (Life Technologies, Carlsbad, USA), and mRNA was purified from total RNA using poly-T oligo-attached magnetic beads. First-strand cDNA was synthesized using a random hexamer primer and M-MuLV Reverse Transcriptase. Subsequently, second-strand cDNA synthesis was performed using DNA Polymerase I and dNTP. The AMPure XP system (Beckman Coulter, Beverly, USA) was used to select cDNA fragments with a length of 370–420 bp for PCR amplification. After PCR procedures, the product was purified by AMPure XP beads to obtain the library. The quality of the library was tested using the Qubit2.0 Fluorometer and Agilent 2100 bioanalyzer. After confirming that the insert size met the expectation, the concentration of the library was quantified using qRT-PCR. The different libraries were pooled according to the effective concentration and the target amount of data off the machine, and then sequenced using the Illumina NovaSeq 6000. For differential expression analysis, the read counts were normalized using the DESeq2 program through the scaling factor. P values were adjusted using the Benjamini & Hochberg method. A padj ≤ 0.05 and |log2(fold change)|≥ 1.5 were set as the threshold for significantly differential expression. The Kyoto Encyclopedia of Genes and Genomes (KEGG) database and Gene Ontology (GO) were used to identify enriched pathways through the clusterProfiler R package (3.8.1). Gene Set Enrichment Analysis (GSEA) is a computational approach to determine significant differences between two samples. The genes were ranked to check for enriched genes at the top or bottom of the Gene Set list.

## Results and discussion

### Design and fabrication of network nanocapsules

The formation of 5-HT polymerization is the basement for protein delivery. Therefore, the pH value for triggering the formation of 5-HT covalent bonds was firstly determined. In brief, the inner-core nanoparticles (NPs) were obtained via the facile one-step method wherein 5-HT was mixed with CAT + HSA (m_CAT_: m_HSA_ = 1:4) in pH 7.4 or pH 9.5 Tris–HCl buffer, and the mixture was kept under stirring for 20 h. The obtained inner-core NPs were incubated with HSA at a mass ratio of 1:1 in phosphate-buffered saline (PBS) to get CAT-loaded nanocapsules (CNCs). In a Tris–HCl buffer solution having pH 9.5, the transparent mixture solution turned brownish, indicating the formation of 5-HT covalent bonds can be easily initiated and CAT can be successfully coloaded. Following the surface absorption of HSA, the obtained nanocapsule was characterized by dynamic light scattering (DLS). The nanocapsules were observed to be 124.3 ± 8.00 nm in hydrodynamic diameters d_h(N)_ with a surface ζ-potential of − 17.47 ± 1.33 mv (Additional file 1: Fig. S1). Conversely, the solution remained transparent at pH 7.4. DLS could only detect free proteins of < 10 nm in hydrodynamic diameters d_h(N)_ with a high negative ζ-potential, indicating the failure of nanocapsule formation. Therefore, the above data showed the nanocapsules from 5-HT polymerization were successfully constructed at pH 9.5 [26–29]. These properties indicate that 5-HT is primarily adsorbed on the protein surface via π-π stacking, hydrogen bonds, and van der Waals forces [30], thereby allowing the formed 5-HT polymerization to load the proteins simultaneously.

**Fig. 1:**
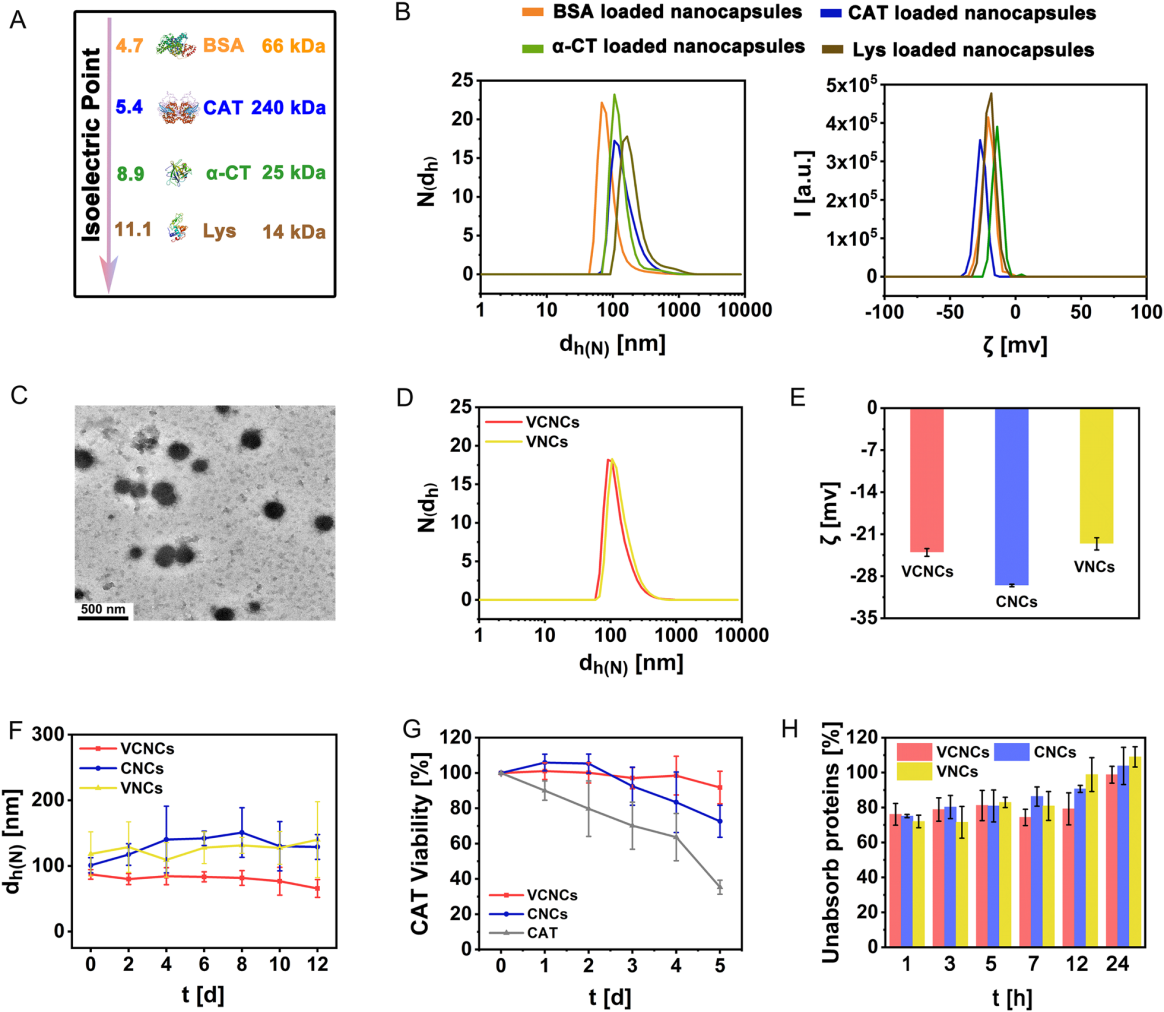
Characterizations of network nanocapsules. **A** Model proteins with different isoelectric points and molecular weights were used in this study. **B** Size distribution N(d_h_) and surface ζ-potential distribution I(ζ) of the nanocapsules loaded with model proteins were measured in ultrapure water at a concentration of C_NPs_ = 1 mg/mL. **C** TEM images of VCNCs. **D** Number distribution N(d_h_) and **E** surface ζ-potential of VCNCs, CNCs, and VNCs were recorded in ultrapure water at a concentration of C_NPs_ = 1 mg/mL. **F** Time-dependent colloidal stability of VCNCs, CNCs, and VNCs (C_NPs_ = 1 mg/mL) in PBS was measured in terms of hydrodynamic size d_h(N)_. **G** Relative CAT viability V_CAT_ in VCNCs, CNCs, and free CAT was measured at the same amount of CAT C_CAT_ = 10 μg/mL in PBS for up to 5 d. Data at each time point were normalized to V = 100% at t = 0 d (n = 3). **H** Time-dependent ratio of anti-BSA absorption of VCNCs, CNCs, and VNCs was measured (n = 3)

Subsequently, we investigated the generality and modularity of our synthetic strategies by loading four kinds of proteins, including bovine serum albumin (BSA), CAT, lysozyme (Lys), and α-chymotrypsin (α-CT) with different molecular weights (from 14 to 240 kDa) and isoelectric points (from 4.7 to 11.1) (Fig. 1A). The obtained nanocapsules loaded with the different proteins were characterized by DLS, and the results demonstrated that the hydrodynamic diameters d_h(N)_ of those NPs were approximately 100–250 nm with a negative ζ-potential (Fig. 1B and Additional file 1: Fig. S2). To further evaluate loading efficiency, the proteins were separately labeled with FITC (Additional file 1: Fig. S3 and Table S1). The nanocapsules loaded with different fluorescent proteins were synthesized according to the above-described method. The loading efficiencies were detected via FITC fluorescence (Additional file 1: Fig. S4 and Table S2). In this case, the data indicated that the formation of 5-HT polymerization is a simple yet effective procedure to cargo different types of protein.

We next constructed nanoantidepressants with CAT. CNCs were incubated with RVG29 (m_CNCs_: m_RVG29_ = 4:1) to obtain the nanoantidepressants (virus-inspired CAT-loaded nanocapsules, VCNCs). Meanwhile, only HSA without CAT was used as the negative control (virus-inspired nanocapsules, VNCs). Transmission electron microscopy (TEM) images confirmed the successful synthesis of nanocapsules with spherical shapes (Fig. 1C and Additional file 1: Fig. S5). Surface modification with RVG29 exhibited negligible impact on the hydrodynamic diameters d_h(N)_ of NPs (Fig. 1D). During the procedures, the hydrodynamic diameters d_h(N)_ of all the NPs was ~ 150 nm. The ζ-potentials of the NPs were reduced by further surface decoration with RVG29 (VCNCs and VNCs) (Fig. 1E). To verify the chemical and structural variations during the synthetic procedures, the ultraviolet–visible (UV–vis) spectra of VCNCs, CNCs, and VNCs were obtained, which displayed a slight blue shift, and the maximum absorbance peaked at a wavelength of 204 nm, attributed to benzene (Additional file 1: Fig. S6A). Fourier transform infrared spectrum further displayed the signals at 1300–1000 cm^−1^ related to the C–O stretching vibration of the phenolic hydroxyl group. The amide II band of the proteins at 1500–1600 cm^−1^ was chiefly derived from the C–N stretching vibration (Additional file 1: Fig. S6B). Proportions of 5-HT, CAT, and proteins in the nanoantidepressant were quantified (Table 1). The concentration of 5-HT was determined by UV–vis spectra using the standard curve (Additional file 1: Fig. S7). The amount of CAT and protein was detected by the test kits. The mass ratio between CAT and 5-HT was 81.90 ± 4.58 (for VCNCs) and 82.74 ± 10.45 (for CNCs). The percentage of 5-HT in VCNCs, CNCs, and VNCs was 14.00 ± 1.11%, 17.95 ± 0.50%, and 14.06 ± 0.87%, respectively. For CNCs, the concentration of C_5-HT_ + C_protein_ was almost equal to the theoretical value (1000 μg/mL). The theoretical percentage of RVG29 should be 20%, while the calculated percentage of RVG29 was approximately 17% for VCNCs and 16% for CNCs (Table 1). Then, the nanocapsules were incubated with PBS for 12 d to assess their colloidal stability. The hydrodynamic diameters d_h(N)_ and ζ-potential remained stable during the measurement, indicating that the obtained nanoantidepressants exhibited high colloidal stability under neutral conditions (Fig. 1F and Additional file 1: Fig. S8). Next, we investigated the formulation of the nanocapsules that improved the stability of the enzymatic activity. The viabilities of CAT V_CAT_ in VCNCs and CNCs were much higher than that of free CAT in PBS at 5 d (Fig. 1G). In a more complex biological environment, mimicked by incubating VCNCs, CNCs, and VNCs in BSA solutions, ~ 100% of unabsorbed protein ratios were observed after 24 h (Fig. 1H). This suggests that through surface modifications with HSA, non-specific protein adsorption on nanoantidepressants can be largely avoid. The above results demonstrate the successful preparation of nanoantidepressants with desirable colloidal stability and high enzyme activity preservation.

**Table 1:** The elemental concentrations of 5-HT, CAT, proteins from the NPs at C_NPs_ = 1 mg/mL

	VCNCs	CNCs	VNCs
C_5-HT_ [μg/mL]	139.99 ± 11.12	179.50 ± 4.98	140.64 ± 8.66
C_CAT_ [μg/mL]	1.71 ± 0.042	2.19 ± 0.23	/
C_CAT_ [μg/mL]/C_5-HT_ [μg/mL]	81.90 ± 4.58	82.74 ± 10.45	/
C_5-HT_ [%]	14.00 ± 1.11	17.95 ± 0.50	14.06 ± 0.87
C_protein_ [μg/mL]	689.31 ± 35.22	829.59 ± 29.40	698.98 ± 12.56
C_5-HT_ + C_protein_ [μg/mL]	829.30 ± 24.86	1009.91 ± 26.56	839.62 ± 8.01

### Stimuli-responsive releases of 5-HT from sacrificial nanocapsules

The strategy of utilizing self-immolative dynamic bonds enables precise drug release at the lesion site through stimuli-responsiveness, thereby enhancing efficacy and minimizing side effects [31, 32]. The depressive neuronal microenvironment is an inflammatory condition with a slightly acidic pH. We hypothesized that self-immolative nanocapsules would degrade under mildly acidic conditions, thereby releasing 5-HT monomers [33]. To further evaluate this stimuli-responsive release, a neutral condition at pH 7.4 was selected to simulate 5-HT release from nanoantidepressant in circulation, whereas pH 6.4, pH 7.4 + ROSUP, and pH 6.4 + ROSUP (reactive oxygen species up, 1:1000) conditions were selected to determine the release in a depressive neuronal microenvironment. These four conditions were adopted to comprehensively delineate the release of 5-HT following systemic delivery. First, we analyzed the transformations of the nanocapsules under acidic and inflammatory conditions using DLS. A mildly acidic buffer at pH 6.4 initially caused an increase in the hydrodynamic diameters d_h(N)_ of the VCNCs (Additional file 1: Fig. S9A). Subsequently, the intermediate aggregation of VCNCs exhibited time-dependent disintegration. Conversely, CNCs and VNCs gradually degraded in the pH 6.4 buffer. CNCs and VNCs then showed pronounced aggregation in the pH 7.4 + ROSUP buffer, whereas VCNCs mildly aggregated in the condition (Additional file 1: Fig. S9B). Next, VCNCs initially formed the aggregation, and the aggregation progressively degraded in pH 6.4 + ROSUP buffer. CNCs and VNCs still formed significant aggregation in pH 6.4 + ROSUP buffer (Additional file 1: Fig. S9C). The results indicated that the pH value degraded nanocapsule, while the ROSUP condition induced nanocapsule aggregation. Compared with VCNCs, CNCs formed significant aggregation under ROSUP conditions. We hypothesized that surface modification with RVG29 would increase the colloidal stability of the nanocapsules under ROSUP conditions. Furthermore, CAT decreased ROS concentration, thereby the aggregation of VCNCs degraded under pH 6.4 + ROSUP condition.

**Fig. 2:**
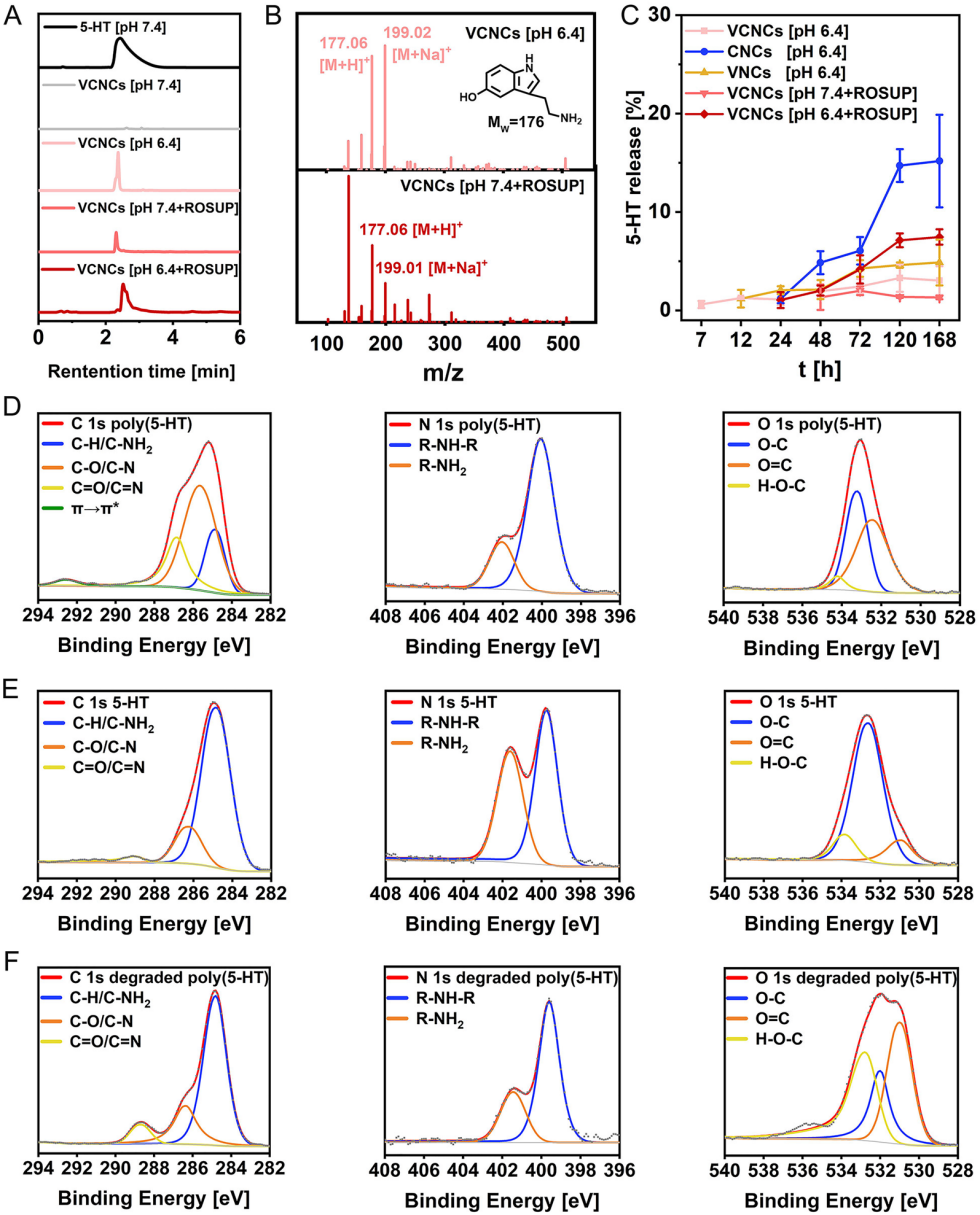
Stimuli-responsive degradation of nanocapsules. **A** The HPLC curve of 5-HT monomers (C_5-HT_ = 500 μg/mL) and VCNCs (C_5-HT_ = 5000 μg/mL) with different conditions for 72 h. **B** The samples at retention time (2–4 min) were collected and analyzed them using ESI–MS. **C** The release profile of VCNCs, CNCs, and VNCs at a concentration C_5-HT_ = 50 μg/mL in different conditions (n = 3). XPS spectra of C 1 s, N 1 s, and O 1 s in **D** poly(5-HT), **E** 5-HT, and **F** degraded poly(5-HT)

**Fig. 3:**
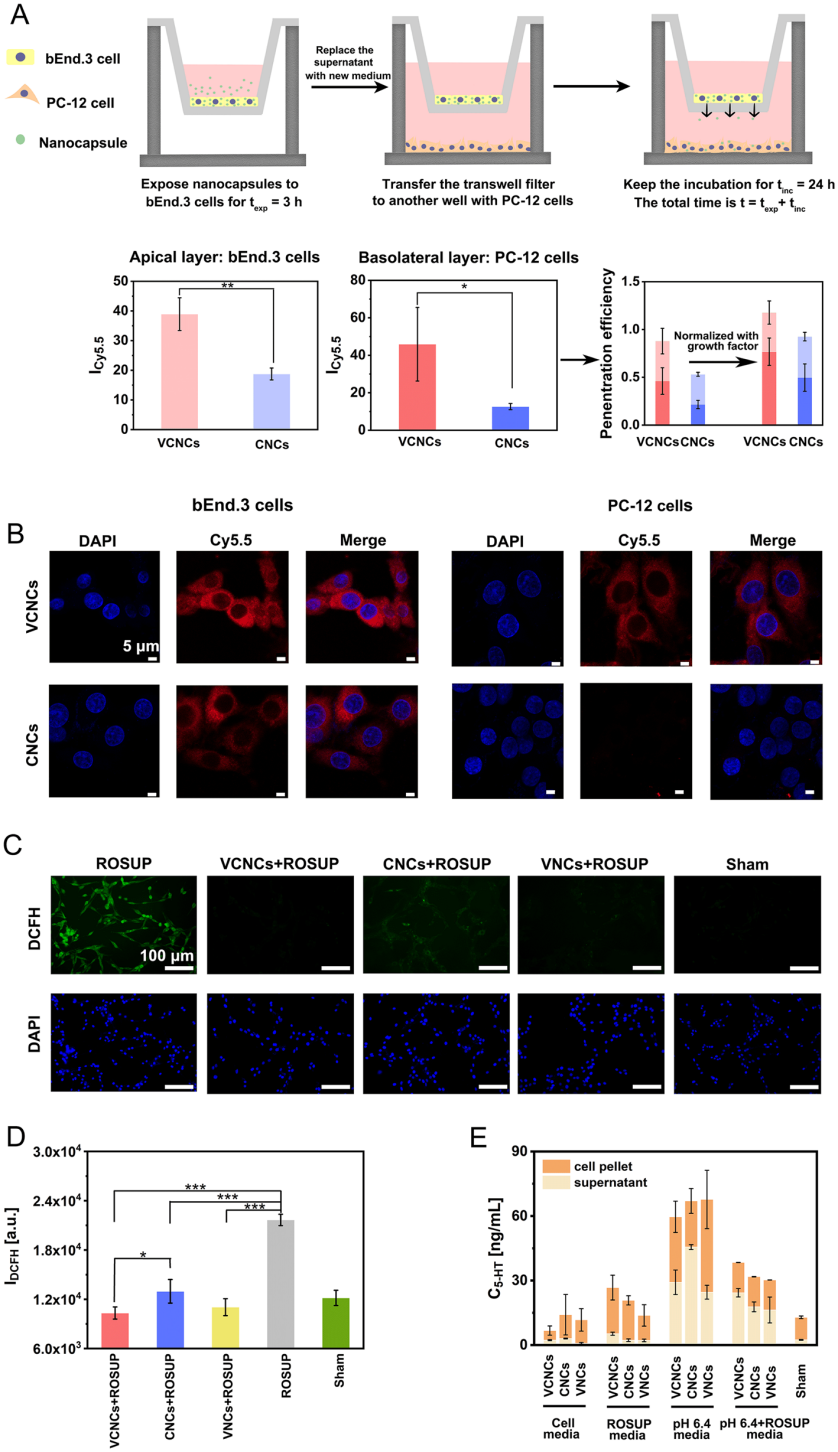
In vitro studies of network nanocapsules. **A** Illustration of an in vitro BBB-crossing model. bEnd.3 cells on the transwell filter were exposed to VCNCs-Cy5.5 and CNCs-Cy5.5 for an exposure time of t_exp_ = 3 h and t_exp_ + t_inc_ = 3 h + 24 h at a concentration of C_5-HT_ = 25 μg/mL. Mean Cy5.5 fluorescence per cell I_Cy5.5_ was measured by flow cytometry for bEnd.3 cells and PC-12 cells. Normalized penetration efficiencies of the nanocapsules were calculated based on these results with growth factors. **B** At t_exp_ + t_inc_ = 3 h + 24 h, confocal images of VCNCs-Cy5.5 and CNCs-Cy5.5 in bEnd.3 and PC-12 cells at C_5-HT_ = 25 μg/mL were obtained, including Cy5.5 channel (red) and DAPI channel (blue). The scale bar represents 5 μm. **C** Fluorescence images and **D** fluorescence intensities of DCFH, corresponding to the ROS level, were detected in PC-12 cells after incubation with VCNCs, CNCs, and VNCs at t_exp_ + t_inc_ = 3 h + 24 h (C_5-HT_: 25 μg/mL, n = 3). The scale bar represents 100 μm. Significant differences between groups were analyzed using the one-way ANOVA method, *P < 0.05, **P < 0.01, ***P < 0.001. **E** The amount of liberated 5-HT was detected in PC-12 cells incubated with VCNCs, CNCs, and VNCs (C_5-HT_: 25 μg/mL) for 3 h in complete cell media, pH 6.4 cell media, ROSUP cell media, and pH 6.4 + ROSUP media (n = 3)

Next, the release of 5-HT monomer at pH 6.4, pH 7.4 + ROSUP and pH 6.4 + ROSUP conditions was detected from high-performance liquid chromatography (HPLC). The appearance of peaks (t = 2–4 min) from three nanocapsules corresponding to 5-HT monomers indicated the disintegration of the nanocapsules (Fig. 2A and Additional file 1: Fig. S10). ESI–MS further confirmed the molecular weight of the monomer (Fig. 2B). Furthermore, the release profile of 5-HT from self-immolative nanocapsules was quantified using HPLC at specific timepoints. The stability of the three nanocapsules was evident by the absence of 5-HT release at pH 7.4 (Additional file 1: Fig. S12). For VCNCs and VNCs, 3.04 ± 1.46% and 4.88 ± 2.32% of 5-HT were released at pH 6.4 for 168 h, respectively (Fig. 2C, Additional file 1: Figs. S13, and S15). We found that CNCs showed 15.18 ± 4.71% liberation of 5-HT under mildly acidic conditions over 168 h (Fig. 3C and Additional file 1: Fig. S14). Despite having similar structures, a faster release of 5-HT from CNCs was observed, suggesting that surface modification with RVG29 protected the nanoantidepressants from degradation. The results showed that 1.32 ± 0.17% of 5-HT was released from VCNCs within 168 h in the pH 7.4 + ROSUP buffer (Fig. 3C and Additional file 1: Fig. S16). Notably, the released 5-HT from CNCs and VNCs in the pH 7.4 + ROSUP buffer over 168 h was barely detectable (Additional file 1: Figs. S10 and S17). This resistance can be attributed to ROS-dependent aggregation that inhibits nanocapsule degradation. It is speculated that CAT protects the released 5-HT from oxidation in the pH 7.4 + ROSUP buffer by decomposing hydrogen peroxide, which is known to prevent oxidative damage. This speculation was further confirmed using UV–vis spectra, which showed that 5-HT lost its structure in the pH 7.4 + ROSUP buffer, whereas CAT acted as an efficient ROS scavenger and inhibited the oxidation of 5-HT (Additional file 1: Fig. S18). In the pH 6.4 + ROSUP buffer, VCNCs exhibited 7.47 ± 0.78% of released 5-HT for 168 h (Fig. 3C and Additional file 1: Fig. S19). Overall, these findings suggest that 5-HT can be successfully released from the self-immolative nanocapsules triggered by mildly acidic and highly inflammatory buffers. The mild acidic condition is the major contributor to nanocapsule degradation, while the low release of 5-HT from VCNCs is promising for alleviating depressive symptoms as an acute increase in 5-HT can induce serotonin syndrome.

To further investigate polymerization and degradation processes, 5-HT was dissolved in a pH 9.5 Tris–HCl buffer without proteins and stirred for 20 h to obtain poly(5-HT). Subsequently, the poly(5-HT) powder was incubated with a pH 6.4 buffer (Citric acid-Na_2_HPO_4_ buffer) for 72 h to produce degraded poly(5-HT). The poly(5-HT) exhibited slight water solubility, whereas degraded poly(5-HT) and 5-HT displayed good solubility in water (Additional file 1: Fig. S20). Due to the insolubility of poly(5-HT) in water, we employed solid-state analysis techniques to investigate their structures, namely X-Ray Photoelectron Spectroscopy (XPS) and FTIR. The XPS spectra of the C 1 s region revealed four distinct peaks, namely C-H/C-NH_2_ (284.8 eV), C–O/C–N (286.1 eV), C=O/C=N (288.2 eV), and π-stacking (292.5 eV). Similarly, the N 1 s region exhibited two peaks corresponding to R-NH_2_ (399.8 eV) and R-NH-R (401.9 eV) for all three samples. It is evident that the primary amine group is prominent in 5-HT, while its intensity diminishes upon polymerization (Fig. 2D and E). In the O 1 s region, three peaks were observed, corresponding to O=C (531.4 eV), O–C (532.6 eV), and H–O–C (533.6 eV). In comparison to 5-HT, poly(5-HT) displayed an increased abundance of O=C and a decreased intensity of H–O–C. These findings collectively indicate that the polymerization of 5-HT is attributed to autoxidation, wherein the primary amine groups and phenolic hydroxyl groups are consumed. On the other hand, the degraded poly(5-HT) exhibits a higher degree of oxidation compared to 5-HT, albeit retaining a portion of primary amine groups and phenolic hydroxyl groups (Fig. 2F). FTIR spectrum further confirmed the presence of primary amine groups and phenolic hydroxyl groups in poly(5-HT), degraded poly(5-HT), and 5-HT (Additional file 1: Fig. S21). The chemical structure of poly(5-HT) was characterized using electrospray ionization-mass spectroscopy (ESI–MS) for the clear supernatant, and the MS spectra indicated the formation of covalent bonds from 5-HT (Additional file 1: Fig. S22). Based on the results and previous publications, we propose two possible mechanisms for the release of 5-HT. Firstly, polymerization is initiated through the auto-oxidation of 5-HT, resulting in the formation of a quinone radical. Subsequently, the quinone radical engages in further reactions with the parent molecules of 5-HT, leading to the generation of dimer intermediates via intramolecular rearrangement. It is worth noting that the amine group present in these dimer intermediates can also undergo a reaction with quinone intermediates, resulting in the formation of an imine structure. Notably, this imine structure exhibits susceptibility to degradation under acidic conditions. Consequently, the degradation process facilitates the release of 5-HT (Additional file 1: Fig. S23A).Secondly, the functional groups of 5-HT, including amino (−NH_2_) and hydroxyl (−OH), participate in intermolecular hydrogen-bonding interactions with the hydroxyl and primary amino groups of poly(5-HT). Additionally, the indole moiety of 5-HT can interact with the aromatic heterocycles of poly(5-HT) through π-stacking. As a result, the unreacted 5-HT monomers can adsorb onto the surface of poly(5-HT) via intermolecular hydrogen-bonding and π-stacking. Upon the degradation of poly(5-HT) induced by acidic conditions, 5-HT can be released (Additional file 1: Fig. S23B).

### In vitro studies of BBB penetrating of nanocapsules

For the in vitro study, we first evaluated the cytotoxicity of the nanocapsules and reagents, and the results exhibited excellent biocompatibility with rat pheochromocytoma PC-12 cells and mouse endothelial bEnd.3 cells (Additional file 1: Figs. S24 and S25). We then investigated the in vivo BBB penetration of nanocapsules. It has been reported that specific binding between RVG29 and nAchR may not only mediate BBB passage but also facilitate cellular uptake [34, 35]. Because bEnd.3 cells overexpress nAchR [36, 37], we then evaluated whether RVG29 modification would enhance the cellular uptake of VCNCs and VNCs. bEnd.3 cells were incubated separately with the three nanocapsules labeled with Cy5.5. The fluorescence intensity of intracellular Cy5.5 was determined using flow cytometry. Following surface modification with RVG29, an obvious shift in fluorescence intensity for VCNCs and VNCs was observed in the bEnd.3 cells, indicating successful receptor-mediated transcytosis (Additional file 1: Fig. S27). The in vitro BBB permeation evaluation was further performed using the transwell system (Fig. 3A). bEnd.3 cells were seeded on the insert transwell filter and PC-12 cells were seeded in the basolateral chamber. bEnd.3 cells were first exposed to VCNCs-Cy5.5 and CNCs-Cy5.5 for 3 h (exposure time t_exp_). Then, the supernatant in the filter was replaced with fresh media and incubated with PC-12 for 24 h (incubation time t_inc_). bEnd.3 cells treated with VCNCs-Cy5.5 for t_exp_ = 3 h exhibited a positive fluorescence signal ratio, 1.64 ± 0.36 times higher than the cells treated with the same amount of CNCs-Cy5.5 (Additional file 1: Fig. S28). At t_exp_ + t_inc_ = 3 h + 24 h, cellular fluorescence inside bEnd.3 and PC-12 cells exposed to VCNCs-Cy5.5 were respectively 2.10 ± 0.42 times and 3.54 ± 1.21 higher than that inside the cells exposed to CNCs-Cy5.5 (Fig. 3A). The penetration efficiencies of the nanocapsules were further calculated using these fluorescence intensities. Because flow cytometry detects fluorescence per cell, cell proliferation needs to be considered (Additional file 1: Fig. S29) [38]. The penetration efficiencies were normalized by the ratio of cell numbers, following which it was revealed that 76.79% ± 14.29% of the targeting VCNCs had penetrated the bEnd.3 cell layer and was detected on the basolateral side (Fig. 3A). Conversely, only 49.74% ± 14.30% of the nontargeting CNCs was found in the basolateral side under the same conditions. The confocal images confirmed the difference between VCNCs and CNCs at t_exp_ + t_inc_ = 3 h + 24 h, indicating that BBB permeation for delivery was achieved via RVG29 modifications (Fig. 3B). To further evaluate the integrity of nanocapsules during BBB permeation, we established the a transwell model with dual-labeled nanocapsules (HSA-Cy5.5 and RVG29-FITC, Additional file 1: Fig. S30). The fluorescence ratio of I_FITC_/I_Cy5.5_ was calculated at t_exp_ and t_exp_ + t_inc_. (Additional file 1: Figs. S31 and S32). Compared with the one at t_exp_, the ratio displayed a significant decrease at t_exp_ + t_inc_, revealing that RVG29 was gradually lost after the BBB transportation.

Cargo CAT in nanocapsules is expected to alleviate neuroinflammation after penetrating BBB. Therefore, we continued to detect the ROS scavenging efficiency of nanocapsules after permeating bEnd.3 cells. Using the method described above, we established the studies again with the transwell model. Intracellular ROS in PC-12 cells at t_exp_ + t_inc_ = 3 h + 24 h was verified by 2ʹ,7ʹ-dichlorofluorescein diacetate (DCFH-DA), which penetrates cell membranes and is oxidized to 2,7-dichlorofluorescein (DCFH) by ROS, producing green fluorescence. The three nanocapsules exhibited an inhibitory effect on total ROS (Fig. 3C and D). Differences of DCFH intensity were found between VCNCs and CNCs, further verifying that RVG29 facilitated BBB penetration. Notably, VNCs without CAT also showed strong ROS scavenging activity. We speculated that this can be attributed to the antioxidation ability of phenolic hydroxyl groups in the 5-HT polymerization. To investigate the reason, we systematically explored the scavengers in the nanocapsules against intracellular ROS. First, PC-12 cells were separately treated with VCNCs, CNCs, VNCs, 5-HT, CAT, and HSA with different concentrations for 3 h to allow sufficient cellular uptake. Cellular DCFH was used to determine ROS using a fluorescence microplate reader and inverted fluorescence microscope. A high fluorescence signal was observed following ROSUP treatment, whereas the intracellular fluorescence intensity of the cells treated with 5-HT decreased significantly, suggesting that the extra ROS was removed by 5-HT alone (Additional file 1: Figs. S33 and S34). It indicated that the phenolic hydroxyl groups in the structure of 5-HT polymerization can be used as the ROS scavengers [39]. These findings also explained that VNCs containing phenolic hydroxyl groups exhibited antioxidative activity during the procedure. A positive correlation between increased intracellular calcium (Ca^2+^) concentrations and oxidative stress indicated that the increase in intracellular Ca^2+^ concentrations is causally related to oxidative stress [40, 41]. Intracellular Ca^2+^ level changes were assessed using the cell-permeable indicator Fluo-4 AM, which exhibits low cytotoxic and high fluorescence. PC-12 cells were separately incubated with nanocapsules and reagents for 3 h, loaded with Fluo-4 AM, fluorescence intensity was detected using the fluorescence microplate reader. All cells treated with the three nanocapsules and CAT exhibited a decrease in intracellular Ca^2+^ concentration, thereby confirming the efficient antioxidant activity of the nanocapsules (Additional file 1: Fig. S35). These results prove that nanocapsules after permeating bEnd.3 cells still can greatly alleviate the ROS concentration.

### Cellular release of 5-HT triggered by mildly acidic and highly inflammatory conditions

Acidic pH and inflammatory response are the two characteristic features of the depressive neuronal microenvironment. The 5-HT polymerization are expected to degrade because of acidic conditions. The above data showed that the stimuli-responsive release of 5-HT can be achieved in buffer solutions. To further address this issue, we incubated PC-12 cells with VCNCs, CNCs, and VNCs in complete, pH 6.4, ROSUP media (1:1000), and pH 6.4 + ROSUP media (1:1000) for 3 h. A window covered the typical routine for drug release, including into circulation fluid (neutral condition) and depressive neuronal microenvironment (pH 6.4 and ROSUP). The concentrations of 5-HT monomers in the cell pellet and supernatant were determined using an enzyme-linked immunosorbent assay (ELISA) kit (Fig. 3E). In line with the degradation behavior of 5-HT in the buffers, a negligible cumulative 5-HT release was observed under neutral conditions in the complete media. Showing positive correlation with the release profiles of VCNCs in ROSUP buffers, a detectable amount of 5-HT was released from VCNCs over 3 h in the presence of ROSUP media. At a mildly acidic pH of 6.4, the nanocapsules exhibited a burst release profile. Among these, CNCs displayed the rapidest release profile, further confirming that RVG29 modification impeded 5-HT release from the nanocapsules. Compared with the cell pellet, the amount of liberated 5-HT was more pronounced in the supernatant under pH 6.4 + ROSUP media. Above all, the results showed the self-immolative nanocapsules were successfully degraded and released 5-HT in pH 6.4 and ROSUP media. Low pH value is the key factor for the degradation of nanocapsules.

### Behavioral improvements in depressed mice treated with nanocapsules

**Fig. 4:**
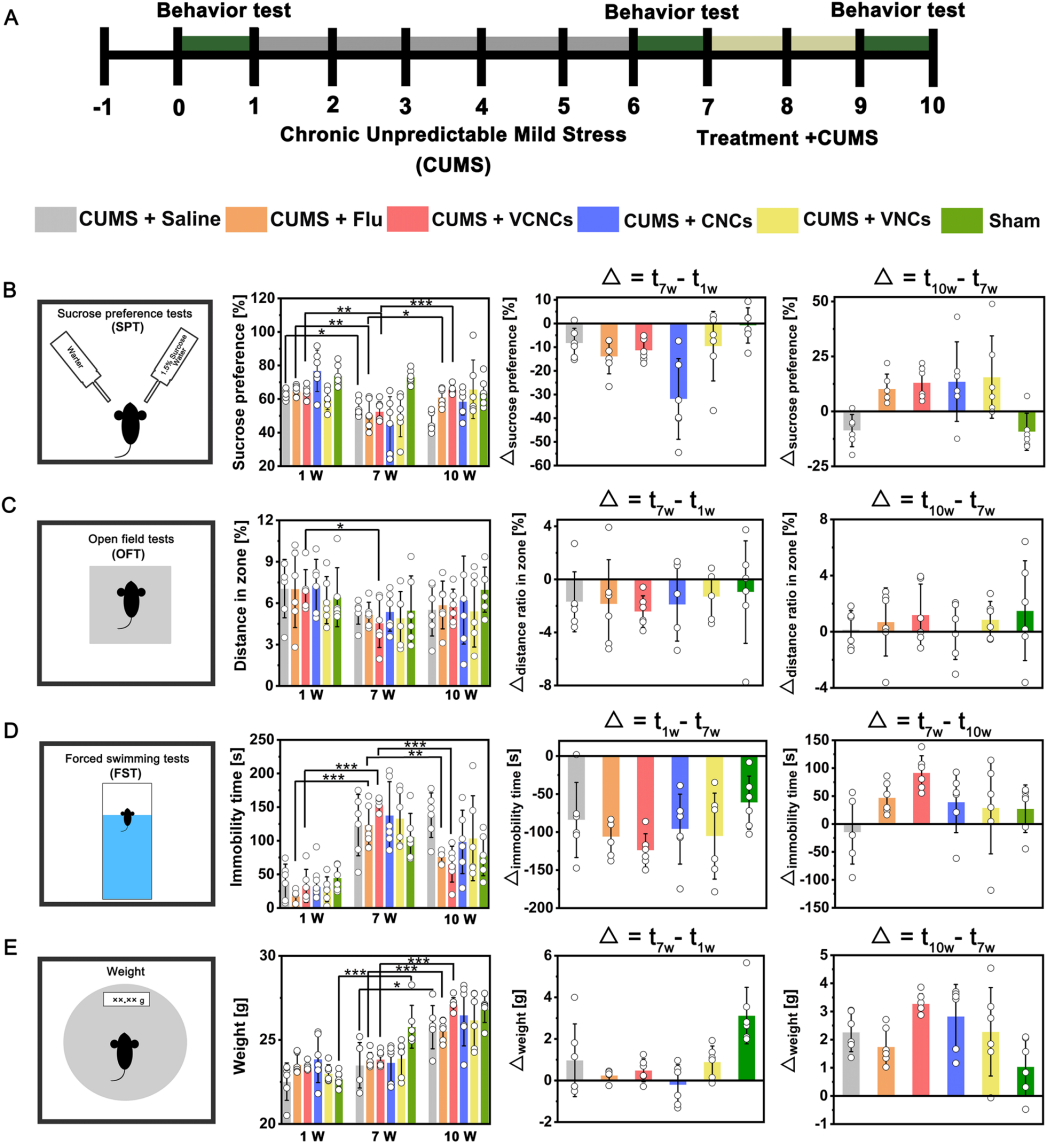
Network nanocapsules alleviate depressive-like behaviors in mice. **A** Experimental timeline of the behavior tests, CUMS procedures, and treatment schedules. Behavior tests comprised **B** SPT, **C** OFT, and **D** FST. **E** Body weight was measured during the procedure (n = 6). The significant differences between groups were analyzed by one-way ANOVA method, *P < 0.05, * *P < 0.01, * * * P < 0.001

After C57BL/6J mice adapted to the new environment for 1 w, several standard assay, such as sucrose preference tests (SPT), a measure of anhedonia; forced swimming tests (FST), a measure of behavioral despairs; and open field tests (OFT), a measure of anxiety [42, 43], were performed to select the mice exhibiting normal locomotor activity. We then administered chronic unpredictable mild stress (CUMS) on the C57BL/6J mice for 5 w to establish a depressive-like state, which is a well-defined mouse model of stress-induced depression (Additional file 1: Tables S6 and S7) [44, 45]. In the CUMS paradigm, mice exhibiting normal behaviors were exposed to a range of unpredictable mild stressors in a semi-random order for weeks, thus triggering diverse behavioral and biochemical changes (Fig. 4A). Antidepressant treatments can subsequently reverse these changes. Consistent with the findings of previous studies, CUMS mice developed depressive-like behaviors, as characterized by decreased sucrose preference in SPT compared with the control mice (Fig. 4B). [46, 47] This behavior reflects major depressive symptoms, such as anhedonia. The depressive-like state observed in CUMS mice was further confirmed by the results of the other two behavior tests, OFT and FST. CUMS mice exhibited a decrease in the ratio of distance traveled in the open section of OFT, reflecting the anxiety symptoms of depressed mice (Fig. 4C). The immobility time of FST was used as an index of behavioral despair. The CUMS procedures largely increased the immobility time (Fig. 4D). Further, CUMS mice were observed to gain significantly less weight over time than the control mice (Fig. 4E). Furthermore, the delta values were calculated from results at 1 w, 7 w and 10 w. It clearly showed the CUMS procedures reduced the sucrose preference, decreased the distance traveled in the zone, increased the immobility time, and mitigated the tendency of gain weight. Taken together, these results demonstrate that CUMS successfully increased the depressive-like and anxiety-associated behaviors in stressed C57BL/6J mice. Next, the mice were divided into six groups (n = 6): sham (not exposed to CUMS), CUMS, CUMS + fluoxetine (CUMS + Flu), CUMS + VCNCs, CUMS + CNCs, and CUMS + VNCs groups. To determine whether the treatments can reverse depressive-like symptoms, behavior assays were conducted again. We used the results from the behavior test at 10 w and 7 w to obtain delta values. Treatments with fluoxetine and the three nanocapsules increased the sucrose preference, extended the distance traveled in the zone, and reduced the immobility time of the mice (Fig. 4B–D). Furthermore, the depressed mice obviously gained weight during the VCNCs treatment (Fig. 4E). Notably, VCNCs exhibited a more prominent antidepressive effect than CNCs and VNCs. VCNCs even showed better therapeutic outcomes than fluoxetine.

### In vivo therapeutic efficacy of nanocapsules

**Fig. 5:**
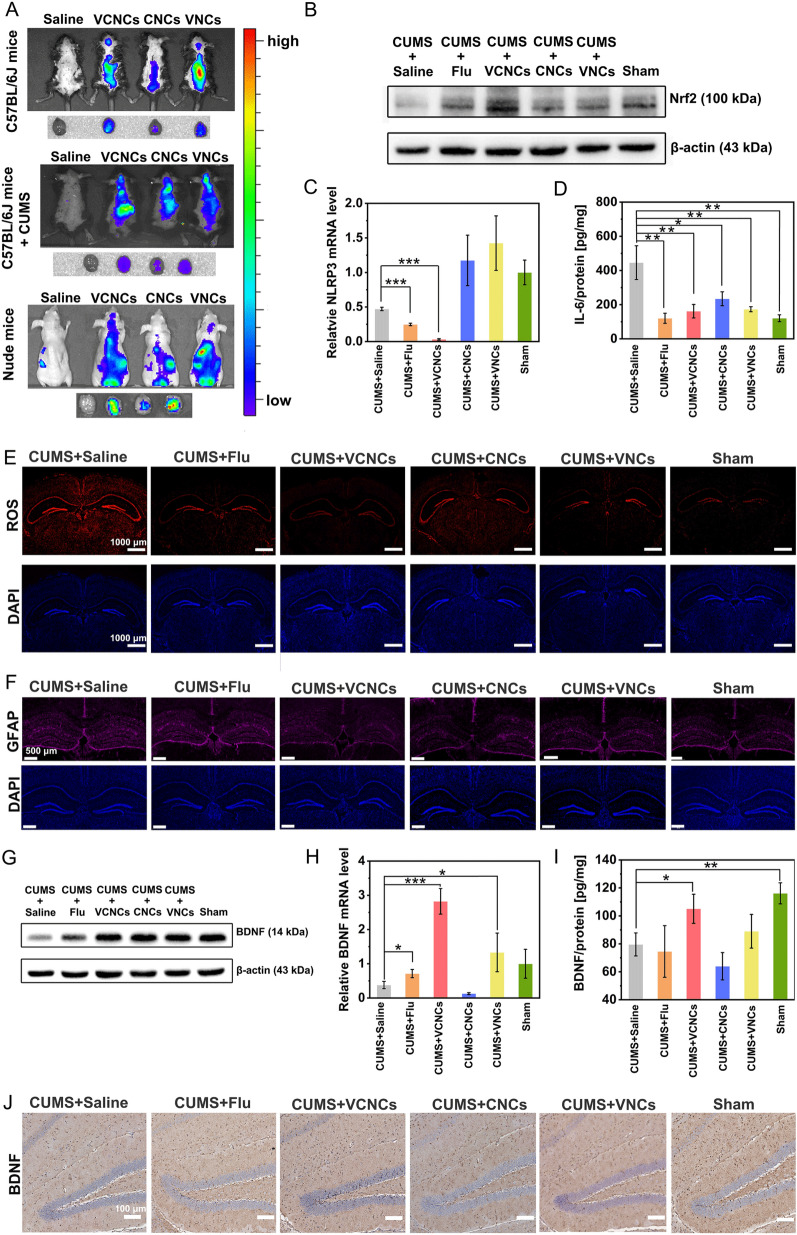
In vivo antidepressive effects of nanocapsules. **A** In vivo Cy7.5 fluorescence of C57BL/6J mice and CUMS mice after intravenous injection with VCNCs-Cy7.5, CNCs-Cy7.5, and VNCs-Cy7.5 at 400 μg 5-HT/kg for 3 h and 7 h. Living Cy 5.5 fluorescence images of mice after treatment with VCNCs-Cy5.5, CNCs-Cy5.5, and VNCs -Cy5.5 (C_5-HT_: 400 μg /kg) for 3 h. The representative western blot analysis of **B** Nrf 2 in the hippocampus of brain across all groups. The other two replicates were presented in Additional file 1: Fig. S38. Reverse transcription quantitative polymerase chain reaction analysis (RT-qPCR) of relative mRNA levels of **C** NLRP3 in the hippocampi of mice in all groups (n = 3). The levels of hippocampal **D** IL-6 in the mice hippocampi after treatment were detected using ELISA kits. The results were normalized by the protein concentration of each sample (n = 3). **E** ROS/DAPI staining and **F** GFAP / DAPI staining brains in groups subjected to different treatments. Analysis of hippocampal BDNF expression using **G** western blot, **H** RT-qPCR, **I** ELISA kits and **J** immunohistochemistry slides. The significant differences between groups were analyzed using the one-way ANOVA method, *P < 0.05, **P < 0.01, *** P < 0.001

First, the brain targeting of RVG29 modification was monitored using IVIS Spectrum in vivo imaging system to detect fluorescence. For the C57BL/6J groups, an obvious Cy 7.5 fluorescence signal was detected in the brain of mice treated with VCNCs-Cy7.5, CNCs-Cy7.5 and VNCs-Cy7.5 after intravenous administration for 3 h, whereas no fluorescence signal was detected in the brains of the CNCs-Cy7.5 treated mice (Fig. 5A). To further confirm the location of the nanocapsules in the brain, ex vivo images of the extracted brains were acquired. The results indicated that the RVG29-modified nanocapsules (VCNCs and VNCs) exhibited higher accumulation than CNCs in the brain. Because brain structure changes have been widely reported in depression [48, 49], we further performed the assays with CUMS mice (Fig. 5A and Additional file 1: Fig. S37). These results confirmed the brain targeting ability of RVG29 modification. We performed the same experiments with nude mice to exclude the interference of the fur (Fig. 5A). The data of nude mice showed good correlation with those of the C57BL/6J groups. Overall, RVG29 ligand could permeate BBB by binding with α7 nAchR.

Next, we assessed the therapeutic efficacy of the nanoantidepressant in detail by measuring biochemical and physiological indicators. Reduced hippocampal volume was the most replicated finding in neuroimaging studies of depression [50]. Given that depression is highly associated with hippocampus dysfunction, we investigated the same using the dissected hippocampus for the study of in vivo therapeutic efficacy. Some patients with depression normally exhibit higher levels of several inflammatory markers, such as tumor necrosis factor (TNF), interleukin-6 (IL-6), and interleukin-1β (IL-1β). It has been widely reported that neuroinflammation contributes to the etiology of depression [51]. Patients with inflammatory illnesses such as coronary artery disease and diabetes also exhibit a higher risk of depression, compared with the general population. [52] Therefore, we further evaluated the i*n vivo* inflammatory factors associated with depression. Previous publications have demonstrated the involvement of the Nrf2 antioxidant pathway in the mechanism of depression [53, 54]. The results revealed a significant decrease in Nrf2 protein expression and mRNA levels in the CUMS group compared to the sham group (Fig. 5B, Additional file 1: Fig. S38A and B). However, treatment with VCNCs significantly increased the Nrf2 at protein and mRNA levels under CUMS procedures. This trend was further confirmed by the ELISA testing (Additional file 1: Fig. S38C). Furthermore, VCNCs significantly decreased the hippocampal NLRP3 expression at the mRNA level than CUMS (Fig. 5C). The expression of IL-6, TNF-α, and IL-1β in the hippocampus of CUMS mice was higher than that in the sham group. Fluoxetine and the VCNCs reduced the concentrations of IL-6, TNF-α, and IL-1β (Fig. 5D and Additional file 1: Fig. S39). In the ROS staining of the brain slice, the CUMS group demonstrated a burst production of ROS, impairing the neurological function of the mice. The ROS levels were significantly lower in mice that received therapeutic treatment than the CUMS group (Fig. 5E and Additional file 1: Fig. S40). Among the treatments, VCNCs reduced the ROS levels to the normal range, similar to that in the sham group.

The neuroinflammatory response induces microglial dysfunction. Over-activated microglia lead to excessive production of pro-inflammatory cytokines, which further enhance neuroinflammation [55]. Furthermore, activated microglia also increase astrocyte activity. [56] To comprehensively study neuroinflammation, we employed glial fibrillary acidic protein (GFAP) and ion calcium-binding adaptor protein 1 (Iba1) as markers for astrocytes and microglia, respectively. The relative mRNA levels of Iba1 were elevated under CUMS procedures, but the groups receiving antidepressive treatments showed a reduction in relative Iba1 mRNA levels during CUMS procedures (Additional file 1: Fig. S41A). Consistently, immunofluorescence images demonstrated an increase in Iba1 signal after CUMS procedures compared to the sham group, indicating microglial activation. A decrease in Iba1 intensity was observed after nanoantidepressant treatments (Additional file 1: Fig. S41B and C). Regarding astrocytes, relative GFAP mRNA levels were elevated after CUMS procedures (Additional file 1: Fig. S41D). A significant increase in GFAP immunofluorescence was observed in the CUMS group, indicating astrocyte activation. The nanoantidepressant treatments largely ameliorated the over-activated astrocytes (Fig. 5F and Additional file 1: Fig. S41E). Among these treatments, VCNCs exhibited a significant effect in alleviating the activated state of glial cells.

Brain-derived neurotrophic factor (BDNF), a secretory protein in the neurotrophin family, is reportedly involved in depression. The idea is based on the phenomenon that reduction in hippocampal BDNF levels is associated with depressive behaviors, and antidepressant treatment is targeted to enhance BDNF expression [57]. CUMS mice displayed lower hippocampal BDNF protein and mRNA levels than the sham group (Fig. 5G–I and Additional file 1: Fig. S42). VCNCs reversed this condition, increasing the BDNF concentrations at the mRNA and protein levels. BDNF immunohistochemistry (IHC) images indicated that the hippocampal BDNF intensity in CUMS group was significantly lower than that observed in the sham group. The administration of VCNCs upregulated the BDNF expression compared with the other treatments (Fig. 5J).

**Fig. 6:**
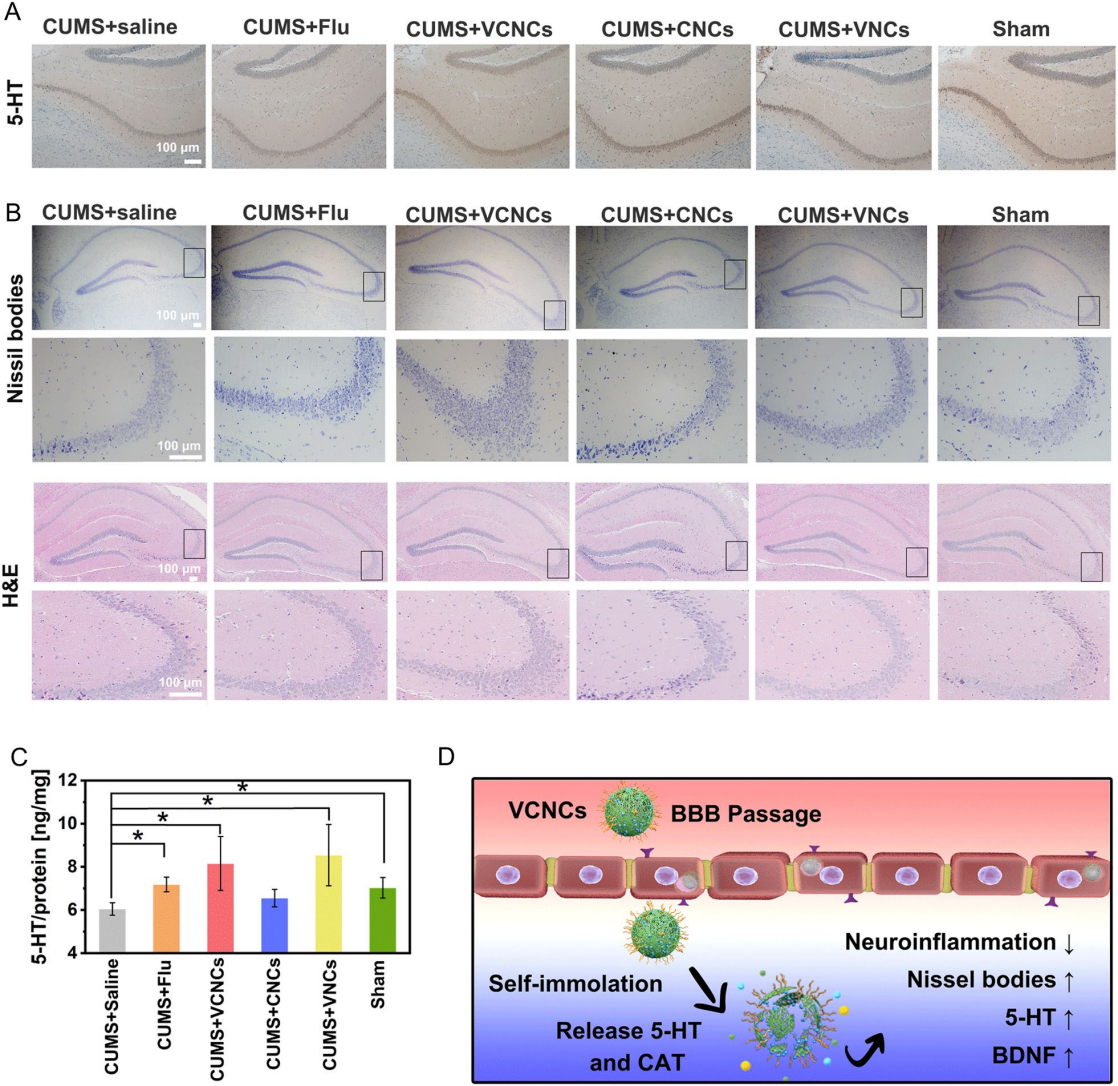
Effects of nanocapsules on in vivo 5-HT level and neuroprotection. **A** 5-HT IHC and **B** nissil and HE staining of brains after administering with fluoxetine, VCNCs, CNCs, and VNCs. **C** The levels of hippocampal 5-HT in the mice hippocampi after treatment were detected using ELISA kits. The results were normalized by the protein concentration of each sample (n = 3). The significant differences between groups were analyzed using the one-way ANOVA method, *P < 0.05. **D** The in vivo therapeutic outcomes of nanocapsules

Classical antidepressant drugs act via increasing the 5-HT levels in the brain by blocking its reuptake and degradation. However, these drugs are marred by certain shortcomings, such as low efficacy and delayed response onset [58]. Therefore, we aimed to achieve the precise delivery of 5-HT to the brain using nanocapsules, which would positively regulate depressive neuronal microenvironment. The 5-HT expression in the brain was determined using an Elisa kit and IHC staining. The images displayed dense accumulations of 5-HT in the CA1 regions of the hippocampi of the sham group. Large reductions in hippocampal 5-HT levels were observed following CUMS procedure. After VCNCs treatment, hippocampal 5-HT levels returned to their normal range (Fig. 6A). The CUMS group showed downregulated expression of hippocampal 5-HT levels compared with the sham group. Subsequently, the administration of fluoxetine, VCNCs, and VNCs increased the hippocampal 5-HT levels in the depressive mice (Fig. 6C). Neuron loss and impairment constitute major depression hallmarks [59, 60]. The hippocampal neurons was assessed using Nissl staining to determine the neuroprotection imparted by the nanocapsules (Fig. 6B). A decrease in the number and density of Nissl bodies was found in the CUMS mice. Following VCNCs, VNCs, and fluoxetine treatment, the Nissl bodies in the CA3 region of the hippocampus showed a clear increase, especially in the VCNCs group; whereas CNCs treatment did not produce such changes. The results were further confirmed via H&E staining, suggesting that VCNCs treatment significantly attenuated neuronal impairment and loss (Fig. 6B). Cortisol is a stress hormone secreted from the hypothalamus pituitary adrenal (HPA) axis. The HPA is considered as a marker of stress response and mediator of pathological consequences [61]. It has been well reported that high levels of cortisol is found in the serum of patients with depression [62]. An increase in the serum cortisol levels was observed in CUMS mice. VCNCs treatment lowered the serum cortisol levels, indicating an alleviation of the depressive symptoms (Additional file 1: Fig. S43). Collectively, the nanoantidepressants inhibit neuroinflammation, increase BDNF concentration, enhance 5-HT levels, reverse neuronal impairment, and suppresses serum cortisol levels (Fig. 6D).

### Genome-scale analysis of antidepressive nanocapsules

**Fig. 7:**
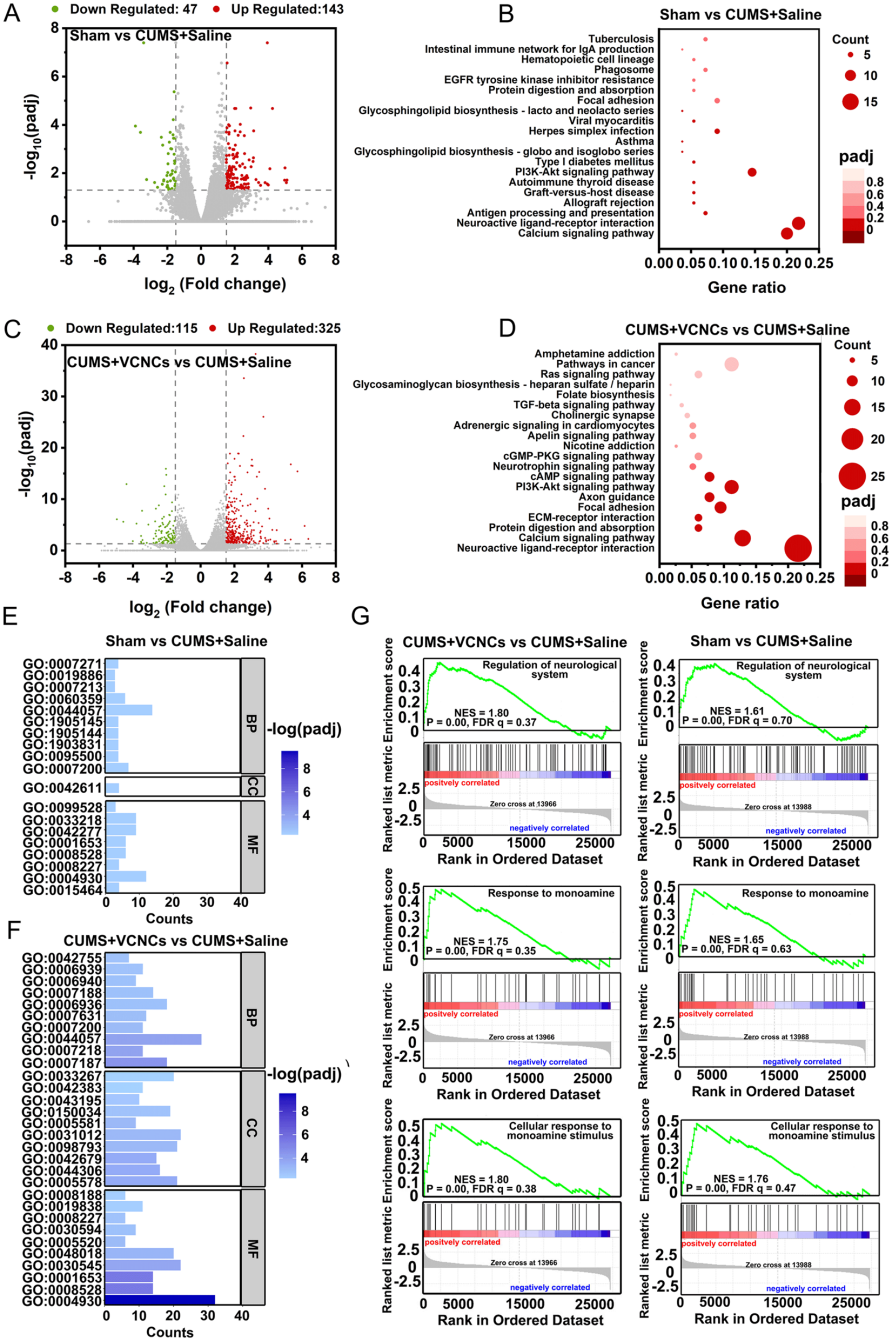
Differentially expressed genes (DEGs) profiles in Sham, CUMS + Saline, and CUMS + VCNCs. **A** Volcano plot comparing Sham vs CUMS + Saline cases. Upregulated genes (n = 143) are represented in red, while downregulated genes (n = 47) are represented in green. **B** The top 20 KEGG pathways for DEGs in the Sham vs CUMS + Saline group. **C** Volcano plot comparing CUMS + VCNCs vs CUMS + Saline cases. Upregulated genes (n = 325) are represented in red, while downregulated genes (n = 115) are represented in green. **D** The top 20 KEGG pathways for DEGs in the CUMS + VCNCs vs CUMS + Saline group. **E** The most significant GO terms associated with DEGs in Sham vs CUMS + Saline and **F** CUMS + VCNCs vs CUMS + Saline groups. The DEGs and significant pathways are identified using the criteria of | log2(FC)|≥ 1.5 and padj < 0.05. **G** GSEA analysis of GO terms applied to the Sham vs CUMS + Saline and CUMS + VCNCs vs CUMS + Saline groups. The results are from three independent samples (n = 3)

To gain a deeper understanding of the molecular mechanisms underlying the therapeutic outcomes of nanoantidepressants, we conducted a genome-scale analysis. The volcano plot showed that 190 mRNAs had a 1.5-fold ratio of Sham to CUMS + Saline (with |log2(FC)|≥ 1.5 and padj < 0.05), with 143 mRNAs upregulated and 47 downregulated (Fig. 7A). Among the differentially expressed genes (DEGs), we identified enriched pathways based on the Kyoto Encyclopedia of Genes and Genomes (KEGG) database (Fig. 7B). The top pathways were associated with the neuron system, such as the neuroactive ligand-receptor interaction and calcium signaling pathway. In contrast, the CUMS + VCNCs group had 440 differential mRNAs compared with the CUMS + Saline group, including 325 upregulated and 115 downregulated (Fig. 7C). The top pathways from the KEGG database still showed that VCNCs treatment significantly enriched the neuroactive ligand-receptor interaction and calcium signaling pathway (Fig. 7D). To further reveal the functional significance of mRNA expression, we performed Gene Ontology (GO) term analysis and established GO terms with a cutoff |log2(FC)|≥ 1.5 and padj < 0.05. The GO analysis showed standardized terms of biological process (BP), cellular component (CC), and molecular function (MF). The enriched GO terms in Sham versus CUMS + Saline and CUMS + VCNCs versus CUMS + Saline were related to neurotransmission, including G-protein coupled receptor activity (GO: 0004930), G-protein coupled amine receptor activity (GO: 000827), and phospholipase C-activating G-protein coupled receptor signaling pathway (GO: 0007200) (Fig. 7E and F). Furthermore, the raw data were run through Gene Set Enrichment Analysis (GSEA), which is the whole-genome ranked list of all available genes without the threshold (Fig. 7G). GSEA highlighted specific changes in neuron function and neurotransmitters, confirming that VCNCs treatment upregulated the neurological system and response to monoamine compared to the CUMS group. The GSEA from the Sham versus CUMS + Saline group showed similar changes. In summary, genome-scale analysis revealed that VCNCs treatment significantly upregulated several pathways closely related to neuron activity.

### In vivo biocompatibility of antidepressive nanocapsules

To evaluate the biosafety of the nanoantidepressants, blood biochemistry assay and hematoxylin and eosin (H&E) staining of the major organs were performed. The blood biochemical indicators, including the levels of white blood cells, neutrophilic granulocyte percentage, and red blood cells, were within the normal range in all the groups (Additional file 1: Table S8). The collected serum was further used to measure the biomarkers of liver and kidney function, including alanine aminotransferase (ALT), aspartate aminotransferase (AST), and blood urea nitrogen (BUN). Compared with the sham group, the nanocapsule-treated groups showed no significant changes (Additional file 1: Fig. S44A–C). In addition, the histological sections of the heart, liver, spleen, lungs, and kidneys via H&E staining revealed no notable evidence of major organ damage (Additional file 1: Fig. S44D).

## Conclusion

Depression is a common, complex, and devastating illness. The depletion of monoamines has been causally related to the etiology of depression. Commercial drugs treat depression by elevating the 5-HT levels in the brain. Unfortunately, the current antidepressant medications exhibit limited clinical efficacy due to delayed response onset, intolerable side effects, and low response rate. Nanotechnology can potentially circumvent the clinical dilemma of antidepressants by accurately delivering the commercial drugs to the brain. Some studies have constructed nanoantidepressants as fluoxetine-loaded NPs to shorten the response duration and minimize the side effects of the antidepressants. However, such efforts have been thwarted by the intrinsic limitations of fluoxetine. Given the pivotal function of neurotransmitters in mood regulation, attention should be paid to the direct and precise supplementation of the brain 5-HT level.

Fundamentally, we designed and fabricated a network nanocapsule using the stimuli-responsive properties of the nanoantidepressants to carry 5-HT and CAT cross BBB. A major advantage of our system is that we adopted the 5-HT polymerization to construct nanocapsules by using a one-step method, which offers more benefits, including improved biocompatibility and versatility in the different payload proteins. Furthermore, the stimuli-responsive nanocapsules maintained their integrity in circulation, while they released 5-HT monomers into the depressive neuronal microenvironment owing to their self-immolation triggered by the acidic conditions. In addition, the nanoantidepressants carry loaded CAT and migrate to the neuronal microenvironment by permeating the BBB using RMT, which can largely alleviate neuroinflammation and enhance 5-HT efficacy. We would like to highlight two therapeutic mechanisms for remodeling the depressive neuronal microenvironment: (i) the released 5-HT monomers from stimuli-responsive degradation can change the abnormality of the 5-HT circuitry and further positively regulate mood and (ii) the delivered CAT with enzyme viability relieves the neuroinflammation and maintains the bioactivity of 5-HT under oxidative conditions. Moreover, this effective approach encourages the promising prospect of using nanotechnology to achieve rapid antidepression therapy, offering useful strategies for different therapeutic treatments for mental health disorders.

### Supplementary Information

**Additional file 1.** Supporting information.

## Data Availability

The datasets that were utilized and/or analyzed during the current study are available from the corresponding author upon reasonable request.

## References

[1] Mishra A, Galhotra A (2018). Mental healthcare act 2017: need to wait and watch. Int J Appl Basic Med Res.

[2] Warren PA, Warren PA (2018). Disability system. Handbook of behavioral health disability management.

[3] Gonçalves-Pinho M, Ribeiro JP, Fernandes L, Freitas A (2022). Depressive disorder related hospitalizations in Portugal between 2008–2015: a nationwide observational study. Psychiatr Q.

[4] Fava M, Kendler KS (2000). Major depressive disorder. Neuron.

[5] Sulzer D, Edwards RH (2005). Antidepressants and the monoamine masquerade. Neuron.

[6] Wong M-L, Licinio J (2004). From monoamines to genomic targets: a paradigm shift for drug discovery in depression. Nat Rev Drug Discov.

[7] Castrén E (2005). Is mood chemistry?. Nat Rev Neurosci.

[8] Nestler EJ, Barrot M, DiLeone RJ, Eisch AJ, Gold SJ, Monteggia LM (2002). Neurobiology of depression. Neuron.

[9] Wong DT, Perry KW, Bymaster FP (2005). The discovery of fluoxetine hydrochloride (Prozac). Nat Rev Drug Discov.

[10] Li X, Frye MA, Shelton RC (2012). Review of pharmacological treatment in mood disorders and future directions for drug development. Neuropsychopharmacology.

[11] Krishnan V, Nestler EJ (2008). The molecular neurobiology of depression. Nature.

[12] Nemeroff CB, Owens MJ (2002). Treatment of mood disorders. Nat Neurosci.

[13] Hodes GE, Kana V, Menard C, Merad M, Russo SJ (2015). Neuroimmune mechanisms of depression. Nat Neurosci.

[14] Martinowich K, Manji H, Lu B (2007). New insights into BDNF function in depression and anxiety. Nat Neurosci.

[15] Jin L, Hu P, Wang Y, Wu L, Qin K, Cheng H, Wang S, Pan B, Xin H, Zhang W, Wang X (2020). Fast-acting black-phosphorus-assisted depression therapy with low toxicity. Adv Mater.

[16] Zhang G, Liu X, Xie W, Hong C, Xu Y, Zhang W, Zhao S, Xin H, Wang X (2021). Trash to treasure: a human beard derived photothermal drug delivery platform for depression therapy. Appl Mater Today.

[17] Liu Y, Hu P, Zheng Z, Zhong D, Xie W, Tang Z, Pan B, Luo J, Zhang W, Wang X (2021). Photoresponsive vaccine-like CAR-M system with high-efficiency central immune regulation for inflammation-related depression. Adv Mater.

[18] Banks WA (2016). From blood–brain barrier to blood–brain interface: new opportunities for CNS drug delivery. Nat Rev Drug Discov.

[19] Ruan S, Zhou Y, Jiang X, Gao H (2021). Rethinking CRITID procedure of brain targeting drug delivery: circulation, blood brain barrier recognition, intracellular transport, diseased cell targeting, internalization, and drug release. Adv Sci.

[20] Furtado D, Björnmalm M, Ayton S, Bush AI, Kempe K, Caruso F (2018). Overcoming the blood–brain barrier: the role of nanomaterials in treating neurological diseases. Adv Mater.

[21] Chen Q, Liu Z (2016). Albumin carriers for cancer theranostics: a conventional platform with new promise. Adv Mater.

[22] Yue Y, Zhao T, Wang Y, Ma K, Wu X, Huo F, Cheng F, Yin C (2022). HSA-Lys-161 covalent bound fluorescent dye for in vivo blood drug dynamic imaging and tumor mapping. Chem Sci.

[23] Hagihara H, Catts VS, Katayama Y, Shoji H, Takagi T, Huang FL, Nakao A, Mori Y, Huang K-P, Ishii S (2018). Decreased brain pH as a shared endophenotype of psychiatric disorders. Neuropsychopharmacology.

[24] Thomsen MS, Weyn A, Mikkelsen JD (2011). Hippocampal α7 nicotinic acetylcholine receptor levels in patients with schizophrenia, bipolar disorder, or major depressive disorder. Bipolar Disord.

[25] Reinhart V, Bove SE, Volfson D, Lewis DA, Kleiman RJ, Lanz TA (2015). Evaluation of TrkB and BDNF transcripts in prefrontal cortex, hippocampus, and striatum from subjects with schizophrenia, bipolar disorder, and major depressive disorder. Neurobiol Dis.

[26] Sardoiwala MN, Mohanbhai SJ, Karmakar S, Choudhury SR (2022). Hytrin loaded polydopamine-serotonin nanohybrid induces IDH2 mediated neuroprotective effect to alleviate Parkinson’s disease. Biomater Adv.

[27] Jeon K, Andoy NMO, Schmitt CW, Xue Y, Barner L, Sullan RMA (2021). Size-controlled synthesis of bioinspired polyserotonin nanoparticles with free radical scavenging activity. J Mater Chem B.

[28] Nakatsuka N, Hasani-Sadrabadi MM, Cheung KM, Young TD, Bahlakeh G, Moshaverinia A, Weiss PS, Andrews AM (2018). Polyserotonin nanoparticles as multifunctional materials for biomedical applications. ACS Nano.

[29] Shi P, Miao X, Yao H, Lin S, Wei B, Chen J, Lin X, Tang Y (2013). Characterization of poly(5-hydroxytryptamine)-modified glassy carbon electrode and applications to sensing of norepinephrine and uric acid in preparations and human urines. Electrochim Acta.

[30] Zhou J, Lin Z, Ju Y, Rahim MA, Richardson JJ, Caruso F (2020). Polyphenol-mediated assembly for particle engineering. Acc Chem Res.

[31] Sun T, Jiang C (2023). Stimuli-responsive drug delivery systems triggered by intracellular or subcellular microenvironments. Adv Drug Deliv Rev.

[32] Wang Q, Serda M, Li Q, Sun T (2023). Recent advancements on self-immolative system based on dynamic covalent bonds for delivering heterogeneous payloads. Adv Healthcare Mater.

[33] Meng Y, Zhu J, Ding J, Zhou W (2022). Polyserotonin as a versatile coating with pH-responsive degradation for anti-tumor therapy. Chem Commun.

[34] Lee C, Hwang HS, Lee S, Kim B, Kim JO, Oh KT, Lee ES, Choi HG, Youn YS (2017). Rabies virus-inspired silica-coated gold nanorods as a photothermal therapeutic platform for treating brain tumors. Adv Mater.

[35] Qu M, Lin Q, He S, Wang L, Fu Y, Zhang Z, Zhang L (2018). A brain targeting functionalized liposomes of the dopamine derivative N-3, 4-bis (pivaloyloxy)-dopamine for treatment of Parkinson’s disease. J Control Release.

[36] Chen W, Zuo H, Zhang E, Li L, Henrich-Noack P, Cooper H, Qian Y, Xu ZP (2018). Brain targeting delivery facilitated by ligand-functionalized layered double hydroxide nanoparticles. ACS Appl Mater Interfaces.

[37] Farshbaf M, Mojarad-Jabali S, Hemmati S, Khosroushahi AY, Motasadizadeh H, Zarebkohan A, Valizadeh H (2022). Enhanced BBB and BBTB penetration and improved anti-glioma behavior of Bortezomib through dual-targeting nanostructured lipid carriers. J Control Release.

[38] Liu Z, Escudero A, Carrillo-Carrion C, Chakraborty I, Zhu D, Gallego M, Parak WJ, Feliu N (2020). Biodegradation of bi-labeled polymer-coated rare-earth nanoparticles in adherent cell cultures. Chem Mater.

[39] Guo Y, Sun Q, Wu F-G, Dai Y, Chen X (2021). Polyphenol-containing nanoparticles: synthesis, properties, and therapeutic delivery. Adv Mater.

[40] Xia T, Kovochich M, Liong M, Mädler L, Gilbert B, Shi H, Yeh JI, Zink JI, Nel AE (2008). Comparison of the mechanism of toxicity of zinc oxide and cerium oxide nanoparticles based on dissolution and oxidative stress properties. ACS Nano.

[41] Zhou M, Zhang T, Zhang B, Zhang X, Gao S, Zhang T, Li S, Cai X, Lin Y (2022). A DNA nanostructure-based neuroprotectant against neuronal apoptosis via inhibiting toll-like receptor 2 signaling pathway in acute ischemic stroke. ACS Nano.

[42] Yang Y, Cui Y, Sang K, Dong Y, Ni Z, Ma S, Hu H (2018). Ketamine blocks bursting in the lateral habenula to rapidly relieve depression. Nature.

[43] Cheng J, Umschweif G, Leung J, Sagi Y, Greengard P (2019). HCN2 channels in cholinergic interneurons of nucleus accumbens shell regulate depressive behaviors. Neuron.

[44] Li S, Luo H, Lou R, Tian C, Miao C, Xia L, Pan C, Duan X, Dang T, Li H (2021). Multiregional profiling of the brain transmembrane proteome uncovers novel regulators of depression. Sci Adv.

[45] Yu H, Chen L, Lei H, Pi G, Xiong R, Jiang T, Wu D, Sun F, Gao Y, Li Y (2022). Infralimbic medial prefrontal cortex signalling to calbindin 1 positive neurons in posterior basolateral amygdala suppresses anxiety- and depression-like behaviours. Nat Commun.

[46] Leng L, Zhuang K, Liu Z, Huang C, Gao Y, Chen G, Lin H, Hu Y, Wu D, Shi M (2018). Menin deficiency leads to depressive-like behaviors in mice by modulating astrocyte-mediated neuroinflammation. Neuron.

[47] Uchida S, Hara K, Kobayashi A, Otsuki K, Yamagata H, Hobara T, Suzuki T, Miyata N, Watanabe Y (2011). epigenetic status of Gdnf in the ventral striatum determines susceptibility and adaptation to daily stressful events. Neuron.

[48] Miguel-Hidalgo JJ, Rajkowska G (2002). Morphological brain changes in depression. CNS Drugs.

[49] Bremner JD (2002). Structural changes in the brain in depression and relationship to symptom recurrence. CNS Spectr.

[50] MacQueen G, Frodl T (2011). The hippocampus in major depression: evidence for the convergence of the bench and bedside in psychiatric research?. Mol Psychiatry.

[51] Miller AH, Raison CL (2016). The role of inflammation in depression: from evolutionary imperative to modern treatment target. Nat Rev Immunol.

[52] Otte C, Gold SM, Penninx BW, Pariante CM, Etkin A, Fava M, Mohr DC, Schatzberg AF (2016). Major depressive disorder. Nat Rev Dis Primers.

[53] Nakayama T, Okimura K, Shen J, Guh Y-J, Tamai TK, Shimada A, Minou S, Okushi Y, Shimmura T, Furukawa Y (2020). Seasonal changes in NRF2 antioxidant pathway regulates winter depression-like behavior. Proc Natl Acad Sci.

[54] Bouvier E, Brouillard F, Molet J, Claverie D, Cabungcal JH, Cresto N, Doligez N, Rivat C, Do KQ, Bernard C (2017). Nrf2-dependent persistent oxidative stress results in stress-induced vulnerability to depression. Mol Psychiatry.

[55] Almeida PGC, Nani JV, Oses JP, Brietzke E, Hayashi MAF (2020). Neuroinflammation and glial cell activation in mental disorders. Brain Behav Immun Health.

[56] Greenhalgh AD, David S, Bennett FC (2020). Immune cell regulation of glia during CNS injury and disease. Nat Rev Neurosci.

[57] Park H (2013). Poo M-m: Neurotrophin regulation of neural circuit development and function. Nat Rev Neurosci.

[58] Licinio J, Wong M-L (2005). Depression, antidepressants and suicidality: a critical appraisal. Nat Rev Drug Discov.

[59] Lee AL, Ogle WO, Sapolsky RM (2002). Stress and depression: possible links to neuron death in the hippocampus. Bipolar Disord.

[60] Grace AA (2016). Dysregulation of the dopamine system in the pathophysiology of schizophrenia and depression. Nat Rev Neurosci.

[61] Jankord R, Herman JP (2008). Limbic regulation of hypothalamo-pituitary-adrenocortical function during acute and chronic stress. Ann N Y Acad Sci.

[62] Schatzberg AF, Keller J, Tennakoon L, Lembke A, Williams G, Kraemer FB, Sarginson JE, Lazzeroni LC, Murphy GM (2014). HPA axis genetic variation, cortisol and psychosis in major depression. Mol Psychiatry.

