# Supplementary material for: Self-immolative nanocapsules precisely regulate depressive neuronal microenvironment for synergistic antidepression therapy
Source: J Nanobiotechnology. 2023 Aug 17;21:274. doi: 10.1186/s12951-023-02008-9 (PMC10433581; doi:10.1186/s12951-023-02008-9)
Supplement: Supplementary file 1 — Additional file 1. Supporting information. [file 12951_2023_2008_MOESM1_ESM.pdf]

Self-immolative nanocapsules precisely regulate depressive neuronal microenvironment for synergistic antidepression therapy

*Ziyao Liu, Bei Chen, Shijun Xiang, Shuo Hu**

Z.Liu, B.Chen, S.Xiang, S.Hu
Department of Nuclear Medicine, Xiangya Hospital, Central South University, Changsha 410008, China
E-mail: hushuo2018@163.com

S. Hu
National Clinical Research Center for Geriatric Diseases, Xiangya Hospital, Central South University, Changsha 410008, China

Z.Liu, S.Hu
Key Laboratory of Biological Nanotechnology of National Health Commission, Xiangya Hospital, Central South University, Changsha 410008, China

Materials and methods

Synthesis protocol of inner-core nanoparticles

To synthesize inner-core nanoparticles, 33.6 mg of 5-HT was dissolved in water at a concentration of $C_{5\text{-HT}}$ = 32 mg/mL. Then, 0.45 mL of the 5-HT aqueous solution was added to 21 mL of Tris-HCl buffer (pH 9.5 or pH 7.4) under vigorous stirring at room temperature. Separately, CAT and HSA were dissolved in PBS (pH 7.4) at a mass concentration of $C_{CAT/HSA}$ = 3.2 mg/mL. Next, 300 μL of CAT solution and 1200 μL of HSA solution were mixed, and after 16 h, 750 μL of the resulting protein solution was added to the reaction mixture. After 1 h, another 750 μL of the protein mixture was added to the reaction mixture. Following another 1 h, 0.6 mL of the 5-HT aqueous solution was added to the reaction mixture. After an additional 2 h of standing with vigorous stirring, the mixture was centrifuged at 1000 rpm for 10 min to remove any remaining aggregated big nanoparticles (NPs) that remained in the black pellet. To purify the inner-NPs, the pellet was removed, and the clear supernatant was kept. Then, the unreacted 5-HT was washed out by membrane dialysis (2 kDa) in ultrapure water at pH 9.5 solution for 6 h, with the ultrapure water at pH 9.5 solution being changed every 2 h.

FITC labeling proteins

To label the proteins with FITC, the fluorophore was dissolved in ethanol, while the proteins were dissolved in Tris-HCl buffer at pH 9.5. The FITC solution was then added immediately to the protein solution and kept under gentle stirring at room temperature for 5 h, using a ratio of 1 mole of FITC per 2 moles of protein. Unconjugated dyes were subsequently removed through membrane dialysis (2 kDa) in ultrapure water for 6 h.

HPLC methods

The mobile phase gradient in HPLC comprised two solvents: Solvent A, a solution of 0.01 M CH_3COONa and 0.02 M citric acid adjusted to pH 4.0, and Solvent B, 100% methanol. The solvents were filtered through a 0.22 μm membrane filter before use in the assay. The eluent consisted of 95% solvent A and 5% solvent B from t = 0-10 min and 5% solvent A and 95% solvent B from t = 10-15 min. The velocity was maintained at 2 mL/min and signals were obtained at a wavelength of 254 nm. To

determine the release profile of VCNCs, CNCs, and VNCs at a concentration of $C_{5\text{-}HT}$ = 50 μg/mL, the samples were incubated at pH 7.4, pH 6.4, pH 7.4+ROSUP, and pH 6.4+ROSUP for 0, 7, 12, 24, 48, 72, 120, and 168 h. After incubation, the samples were ultracentrifuged with a 3 kDa tube at 8000 rpm for 30 min, and the eluents were collected and quantified using HPLC as described above. First, a calibration curve for 5-HT was prepared using 5-HT monomers (Alaadin, #S111161, China) at different concentrations ($C_{5\text{-}HT}$: 31.25 - 0.49 μg/mL) in ultrapure water. The peak areas at retention time 2-4 min were plotted against the concentration of 5-HT as determined by weighing. Finally, the liberated 5-HT was calculated from the peak area at 2-4 min of each sample according to the standard curve.

Structure analysis of poly(5-HT), degraded poly(5-HT), and 5-HT

To comprehensively investigate the polymerization of 5-HT in this study, we synthesized poly(5-HT) using the same method without the presence of proteins. In brief, 0.45 mL of the aqueous solution of 5-HT ($C_{5\text{-}HT}$ = 32 mg/mL) was added to 21 mL of Tris-HCl buffer (pH 9.5) and vigorously stirred at room temperature for 18 h. Furthermore, 0.6 mL of the 5-HT aqueous solution was added to the reaction mixture and vigorously stirred for 2 h at room temperature. The resulting mixture was then centrifuged at 1000 rpm for 10 min, and the supernatant was retained and purified by membrane dialysis (2 kDa) in ultrapure water at pH 9.5 for 6 h. The product was obtained through lyophilization. Subsequently, the powder of poly(5-HT) was incubated with a pH 6.4 buffer (Citric acid-Na_2HPO_4 buffer) for 72 h. The mixture was then centrifuged using an ultracentrifuged tube (with a cutoff molecular weight of 3 kDa) at 8000 rpm for 30 min. The eluent was collected, and the degraded poly(5-HT) was obtained through lyophilization. The structure of poly(5-HT), degraded poly(5-HT), and 5-HT was measured using FTIR (Nicolet is 50, Thermo Scientific, USA), X-Ray Photoelectron Spectroscopy (XPS, ESCALAB Xi+ , ThermoFischer, USA), Electrospray Ionization Mass Spectroscopy (ESI-MS, LTQ Orbitrap Velos Pro, Thermo Scientific, USA). For XPS analysis, the spectrometer employed an AI Kα X-ray source (hv=1486.6eV) under ultrahigh vacuum conditions (8×10^{-10} Pa). Spectra were acquired with a pass energy of 100 eV, using 0.05 eV steps for the survey spectra and 30 eV for high-resolution spectra of C 1s, O 1s, and N 1s. In addition, the poly(5-HT) sample was centrifuged at 1000 rpm for 10 minutes, and the supernatant was collected for ESI-MS analysis.

Fluorescence labeling

The Cy5.5 NHS or Cy7.5 NHS was dissolved in dimethylformamide (DMF) at a concentration of 1 mg/mL, and the HSA was dissolved in Tris-HCl buffer (pH = 9.5) at a concentration of 5 mg/mL (50 mg in 5 mL, 150.38 μM of HSA). Under gentle stirring, 269.3 μL of the Cy5.5 NHS solution (75.19 μM of Cy5.5 NHS) or 294.1 μL of the Cy7.5 NHS solution (75.19 μM of Cy7.5 NHS) was immediately added to the protein solution. The mixture was kept stirring for 5 h. Free fluorophores were separated from labeled proteins by membrane dialysis with a 2 kDa cutoff. Next, we used lyophilization to obtain the Cy5.5 labeled HSA (HSA-Cy5.5) and Cy7.5 labeled HSA (HSA-Cy7.5). The labeling efficiency was characterized by the UV-vis spectrum and fluorescence spectrum at 678 nm excitation and 788 nm excitation, respectively (Fig. S26 and Fig. S36). Next, 866 μL of FITC/DMF solution at a concentration C_{FITC} = 1 mg/mL (445 μM) and 15 mg of RVG29 (890 μM) were dissolved in 5mL of pH 9.5 buffer (Tris-HCl buffer). The reaction mixture was allowed to stir for 5 h at RT. The unlabeled FITC was removed by membrane dialysis with a 2 kDa cutoff for 6 h. The final product, FITC-labeled RVG29 (RVG29-FITC), was dried through lyophilization. Then, we used HSA-Cy5.5 and RVG29-FITC to synthesize double-labeled VCNCs and VNCs by the method described above. The FITC labeling efficiency was also tested by the UV-vis spectrum and fluorescence spectrum (Fig. S30).

Cellular anti-inflammation by incubating with nanocapsules and reagents

PC-12 cells were seeded into 24-well plates (surface area/well = 1.9 cm^2) at a density of 150,000 cells/well with a volume of 1 mL and were incubated overnight. Then, the cells were respectively exposed to VCNCs, CNCs, VNCs, 5-HT, CAT, and HSA at different concentrations and incubated for 3 h. The specific concentrations of the samples used for the study are provided in Table S5. After exposure, the cell medium was removed, and the cells were washed twice with 1 mL of PBS. The cells were then incubated with 1.0 mL FBS-supplemented medium containing 1.0 μL ROSUP reagent for 0.5 h at 37°C. As a reference control, one group of cells was not exposed to samples or the ROSUP reagent. We then used DCFH-DA to detect the level of ROS. The data were recorded using the fluorescent microplate reader (Envision@2015, PerkinElmer, USA). Furthermore, the intracellular ROS level was visualized using the inverted fluorescence microscope (Eclipse Ti2, Nikon, Japan) at the FITC channel.

Behavioral tests

To establish SPT, we used the two-bottle free-choice method. Mice were individually housed during the measurement and trained to adapt to two bottles of 1.5% sucrose solution (w/v) in each cage for 2 h, 48 h prior to the test. Before 24 h, mice were trained to adapt to one bottle of 1.5% sucrose solution (w/v) and one bottle of ultrapure water for 2 h. Following this, mice were completely deprived of food and water, and presented with two pre-weighed bottles for 2 h, one containing 1.5% sucrose solution and the other containing pure water. The bottles were weighed again after the measurement, and the sucrose preference was calculated as a percentage using the following equation: Sucrose preference [%] = sucrose consumption / (sucrose consumption + water consumption) × 100. For OFT, each mouse was placed in a black open box (60 cm × 40 cm × 30 cm) within a quiet room. The testing session lasted 5 min, preceded by a 3-min habituation session. The total traveled distance was recorded and analyzed using Smart V03, while the percentage of the distance traveled in the center zone was used to measure locomotion in the open field. In FST, mice were placed individually in a plastic cylinder (30 cm height × 11 cm diameter) containing water at 25°C in a quiet room. The mice were allowed to adapt to the environment for 15 min before 24 h. During the measurements, the mouse was placed in the water and forced to swim for 5 min, and the immobility time within this period was recorded using the video tracking system. Smart V03 was used for the analysis of immobility time.

Western blot analysis

To detect protein expression, the hippocampus samples were homogenized in RIPA buffer containing phosphatase inhibitors. The lysates were then centrifuged at 15,000g for 15 min at 4 °C to remove cellular debris. The resulting supernatants were diluted 20 times and subjected to the Bradford assay (Beyotime, #P0006C, China) for protein quantification. For protein separation, 40 μg of protein was loaded onto SDS-PAGE gels, with 8% polyacrylamide gel used for Nrf2 and 12% polyacrylamide gel used for BDNF. The gels were subsequently transferred onto PVDF membranes using standard procedures, with a transfer time of 120 min at 200 mA for Nrf2 and 40 min at 200 mA for BDNF. The PVDF membranes were then subjected to immunoblotting by incubating them with primary and secondary antibodies. Immunoreactive bands were visualized using enhanced chemiluminescence (Beyotime, #P0018AS, China) and

captured using a gel documentation system (Bio-Rad). The following antibody dilutions were used: 1:2000 for rabbit polyclonal antibodies against Nrf2 (Proteintech, #16396-1-AP, China), 1:1000 for rabbit monoclonal antibodies against BDNF (Abcam, #ab108319, UK), 1:2000 for rabbit polyclonal antibodies against β-actin (Proteintech, #120536-1-AP, China), and 1:2000 for goat anti-rabbit IgG (H+L) peroxidase/HRP-conjugated secondary antibodies (Elabscience, #E-AB-1003, China).

Slice staining

The ROS fluorescent staining was performed on frozen brain slides. The slides were left at room temperature for 30 min, then incubated with anti-fluorescence quenching agents for 5 min and washed with running water for 10 min. Next, the slides were stained with ROS fluorescence solution (Sigma, #D7008, United States) at 37°C for 30 min, followed by incubation with DAPI solution (Servicebio, #G1012, China) at room temperature for 10 min. The slides were then washed with PBS three times for 5 min each. Lastly, the slides were visualized using the fluorescent imaging system (Eclipse C1 and DS-U3, Nikon, Japan).

For immunofluorescence staining, the frozen slides were allowed to reach room temperature for 30 min. Subsequently, the slides were incubated with PBS for 10 min. Following this, permeabilization agent (Proteintech, #PR30007, China) was applied to the slides for 20 min, followed by block solution (Proteintech, #PR30008, China) for 1 h. Next, the slides were incubated overnight at 4 °C with the primary antibodies. The dilutions of the primary antibodies used were as follows: 1:200 for goat polyclonal antibodies against Iba 1 (Abcam, #ab5076, UK) and 1:500 for rabbit polyclonal antibodies against GFAP (Abcam, #ab7260, UK). After incubation, the slides were washed three times with PBS for 10 min each. For the subsequent step, the slides were incubated with the secondary antibodies for 1 h at 37 °C. The dilutions of the secondary antibodies used were as follows: 1:500 for donkey anti-goat IgG H&L (Alexa Fluor® 488) (Abcam, #ab150129, UK) and 1:250 for donkey anti-rabbit IgG(H+L) (CoraLite594) (Proteintech, #SA00013-8, China). After incubation, the slides were washed three times with PBS for 10 min each. Finally, the slides were mounted with an anti-fading agent containing DAPI (Solarbio, #S2110, China).

For Hematoxylin-eosin staining (HE) staining, the first step is the dewax procedure. The organ slides were placed at 60°C for 2 h, followed by deparaffinization with xylene (20 min for three times) and alcohol solutions with gradient concentrations (5 min in 100% ethanol twice, 5 min in 90%, 80%, and 70% ethanol) and rehydration in ultra-pure water (5 min). The slides were then treated with PBS three times for 5 min. Afterward, the slides were stained with hematoxylin for 10 min, washed with running water for 1 min, differentiated with 1% hydrochloric acid ethanol for 30 seconds, washed with running water for 1 min, placed with blue returning liquid (Servicebio, #G1040, China) for 30 seconds, and washed with running water for 1 min. Thereafter, the slides were stained with eosin (Biosharp, #BL700A-2, China) solution for 2 min. The final step is dehydration, where the tissue slides were immersed in gradient concentrations of ethanol (5 min in 100% ethanol twice, 5 min in 90%, 80%, and 70% ethanol) and fresh xylene (10 min). Finally, the slides were sealed with neutral gum. For brain slides, the samples were visualized by Vectra Polaris (Akoya Bioscience, USA), while for major tissue slides, the samples were captured by a microscope (Zeiss, Germany).

For immunohistochemistry (IHC) staining, the dewax procedures were conducted after obtaining the paraffin slides, which are described in HE staining. The slides were microwaved in EDTA solution (pH = 9) for 20 min to repair antigens, washed with ultra-pure water three times for 5 min, and incubated with 3% hydrogen peroxide solution for 10 min. Next, slices were washed with ultra-pure water twice for 5 min, PBS once for 5 min, and blocked with 10% goat serum (Solarbio, #SL038, China) for 1 h at room temperature. Then, the slides were covered with primary antibodies, including anti-BDNF (Abcam, #ab108319, UK, 1:200) and 5-HT (Serotonin) Rabbit Antibody (Immunostar, #20080, USA, 1:2000), and incubated at 4°C overnight. On the next day, the slides were put at 37°C for 30 min, removed the primary antibodies, covered with the secondary antibodies (1:400, Goat Anti-Rabbit IgG (H+L) (peroxidase/HRP conjugated, Elabscience, China), and incubated for 30 min at 37°C. Then, the slides were washed with PBS three times for 5 min, stained with a DAB kit (ZSGB-BIO, China), and washed with running water for 3 min. Finally, the slides were stained with hematoxylin and dehydrated with alcohol and xylene, following the protocols described in HE staining.

For Nissl staining, the slides were first dewaxed using the procedures described in HE staining. Subsequently, the slides were stained with the Nissl kit for 10 min at room temperature, followed by two washes with ultrapure water for 5 min each. Lastly, the slides were dehydrated using the methods described in HE staining.

Supplementary Figures and tables

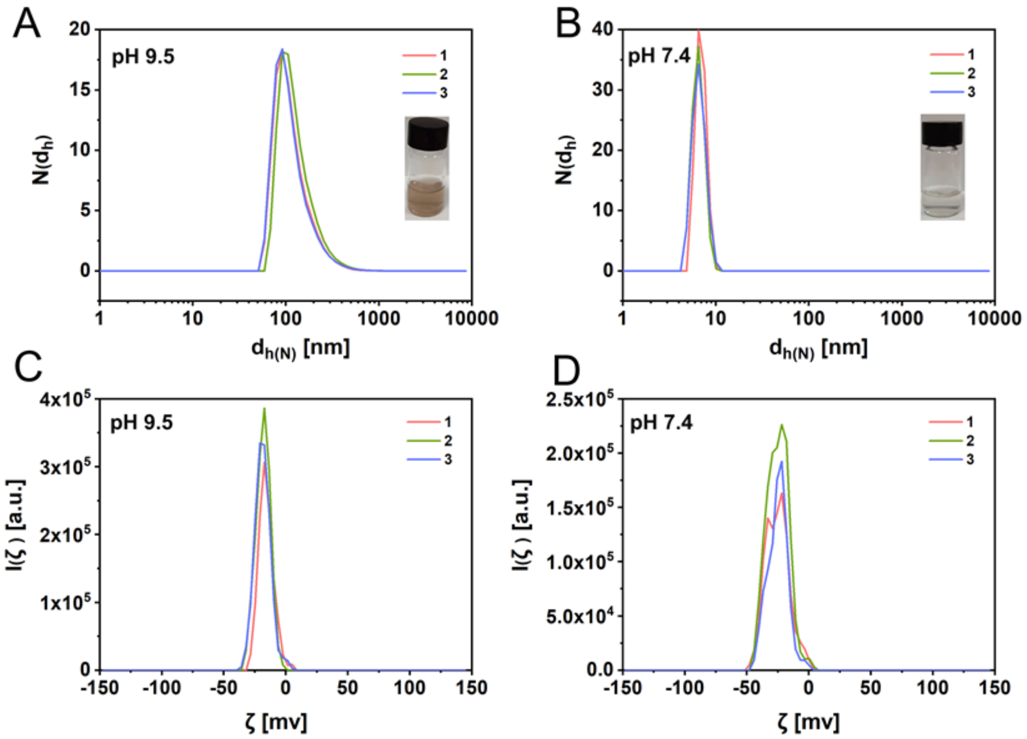

Fig. S1. The number distribution N(d_h) of hydrodynamic diameters of CNCs synthesized in (A) pH 9.5 and (B) pH 7.4 buffers. The distribution of ζ-potential I(ζ) of CNCs synthesized in (C) pH 9.5 and (D) pH 7.4 buffers.

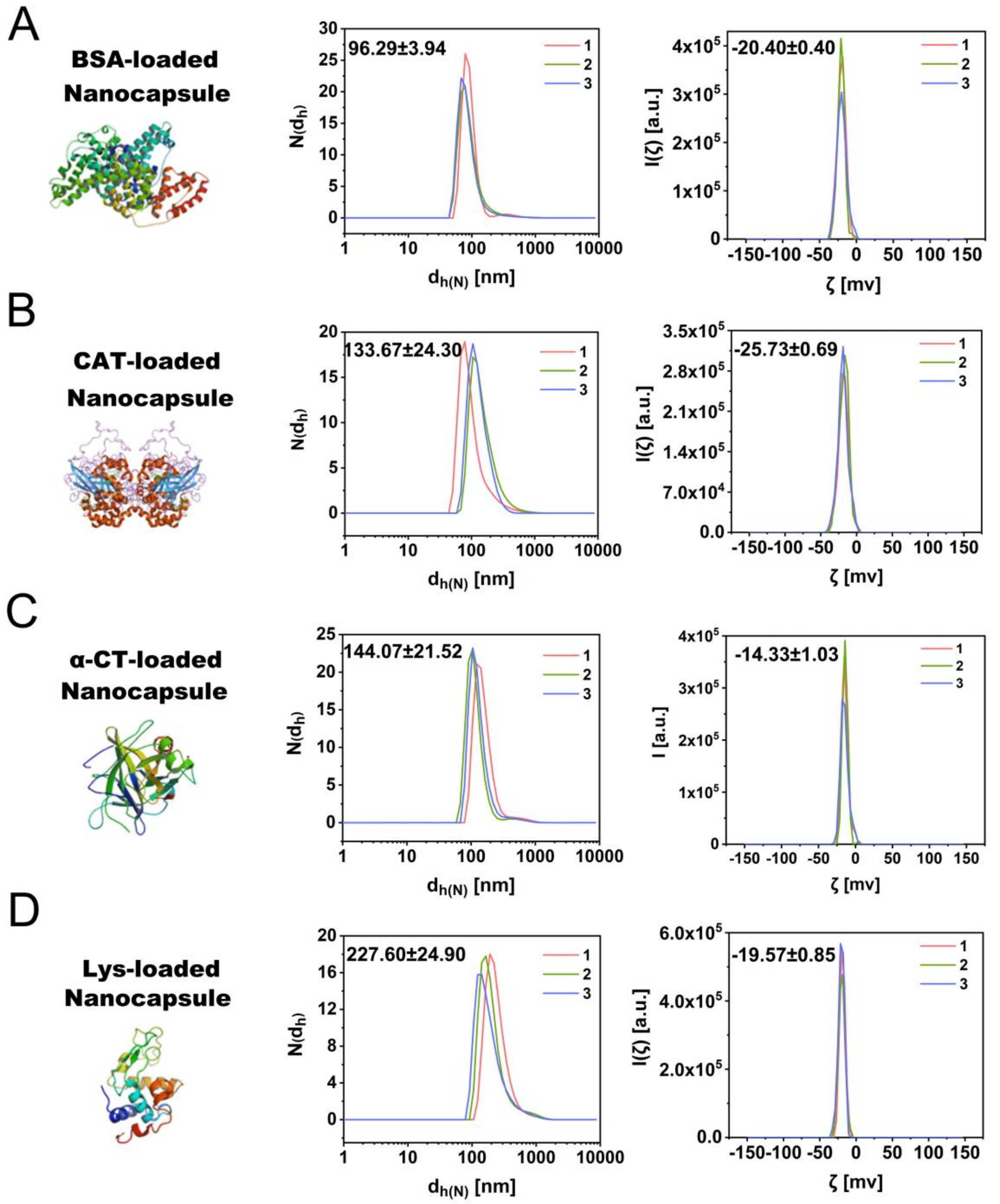

Fig. S2. Compilation of data from Fig. 1B. The number distribution of hydrodynamic diameters $N(d_h)$ and the distribution of ζ-potential $I(\zeta)$ of nanocapsules loaded with (A) BSA, (B) CAT, (C) α-CT and (D) Lys at a concentration C_{NPs} = 1mg/mL.

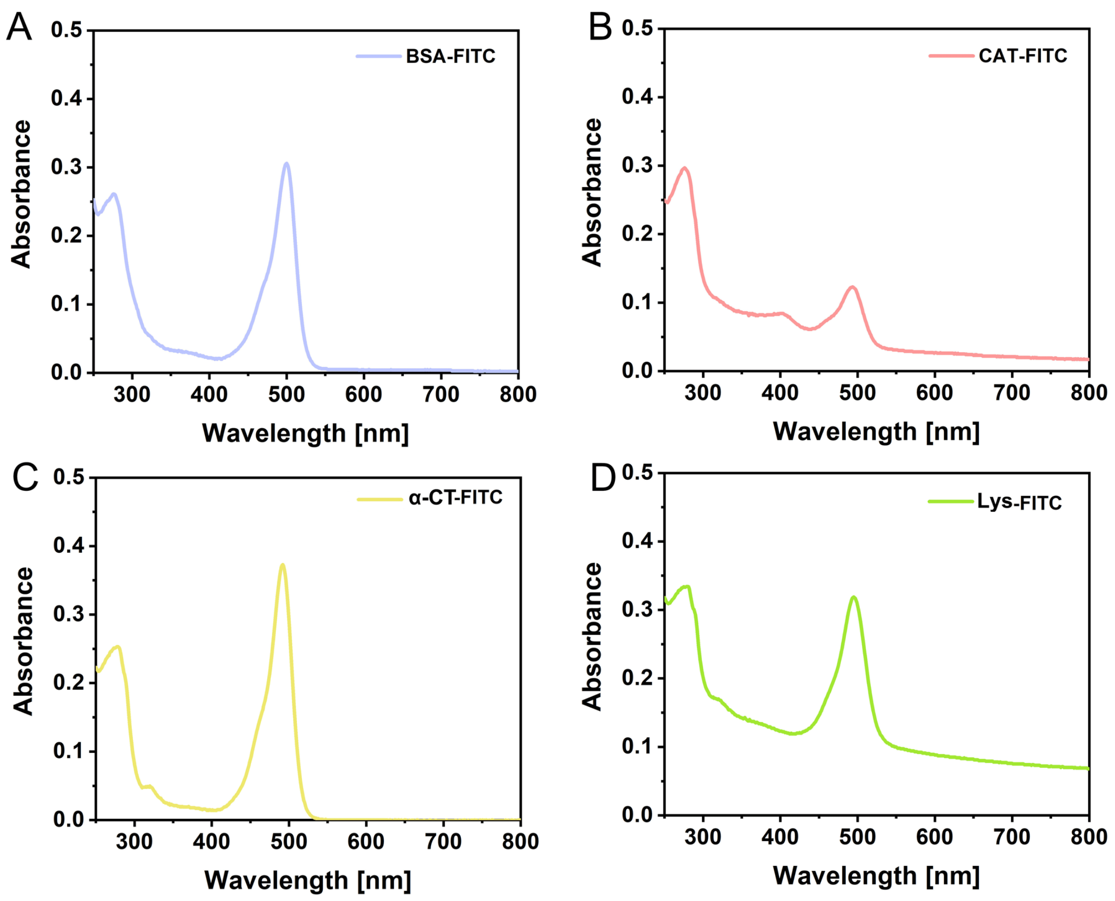

Fig. S3. UV-vis spectrum of (A)BSA-FITC, (B) CAT-FITC, (C)α-CT-FITC, and (D)Lys-FITC at concentration $C_{protein\text{-}FITC}$ = 1 mg/mL.

Table S1. The labeling efficiency of proteins-FITC is calculated from Beer-Lambert's law.

BSA-FITC		CAT-FITC		α-CT-FITC		Lys-FITC	
$\varepsilon_{FITC,492}$	75000	$\varepsilon_{FITC,492}$	75000	$\varepsilon_{FITC,492}$	75000	$\varepsilon_{FITC,492}$	75000
$\varepsilon_{BSA,280}$	43824	$\varepsilon_{CAT,280}$	24600	$\varepsilon_{\alpha\text{-}CT,280}$	50000	$\varepsilon_{Lys,280}$	38940
A_{280}	0.25	A_{280}	0.29	A_{280}	0.25	A_{280}	0.33
A_{492}	0.26	A_{492}	0.12	A_{492}	0.37	A_{492}	0.31
C_{BSA}	5.77×10^{-6}	C_{CAT}	1.18×10^{-5}	$C_{\alpha\text{-}CT}$	5.04×10^{-6}	C_{Lys}	8.58×10^{-6}
C_{FITC}	3.52×10^{-6}	C_{FITC}	1.64×10^{-6}	C_{FITC}	4.97×10^{-6}	C_{FITC}	4.19×10^{-6}
C_{FITC}/C_{BSA}	0.61	C_{FITC}/C_{CAT}	0.14	$C_{FITC}/C_{\alpha\text{-}CT}$	0.99	C_{FITC}/C_{Lys}	0.49

The labeling efficiency of α-CT-FITC has a significant difference between the theoretical result. α-CT easily precipitate in pH 9.5 Tris-HCl buffer, which is close to its isoelectric points (8.9).

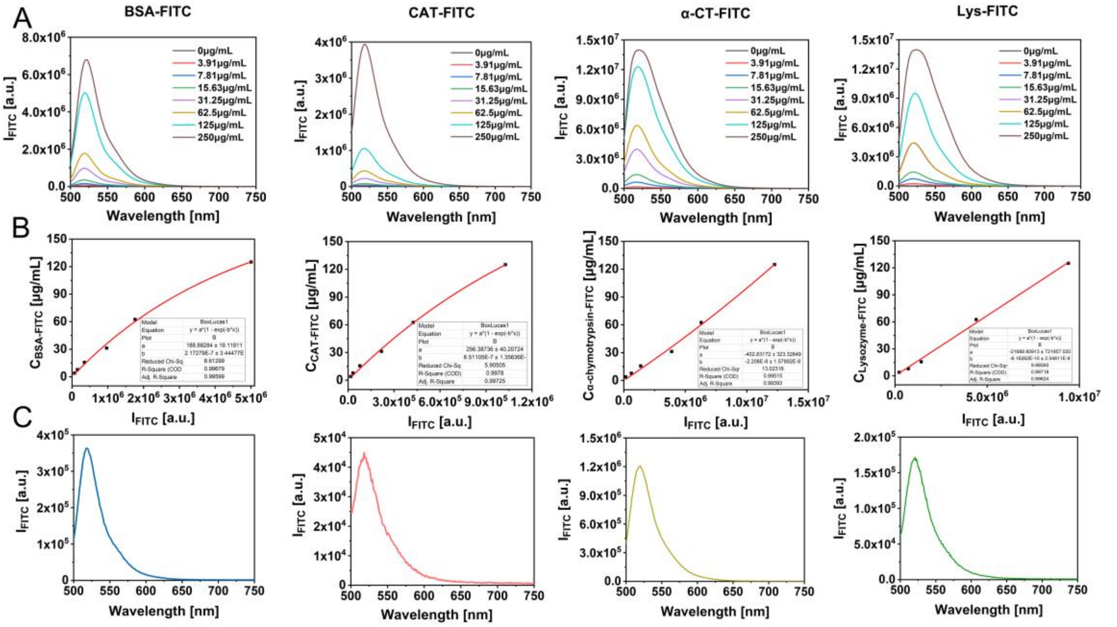

Fig. S4. The payload efficiencies of nanocapsules. (A) Fluorescence spectra of FITC intensity I_{FITC} at 488 nm excitation of free proteins-FITC at different concentrations $C_{protein}$ dispersed in PBS. (B)Fluorescence intensity I_{FITC} (488 nm excitation,520 nm emission) plotted versus the protein mass concentration $C_{poteins}$ via Boxlucas 1 model. The equations are represented as follows, $C_{BSA\text{-}FITC} = 188.88\times(1\text{-}\exp(-2.17\times10^{-7}\times I_{FITC}))$, $R^2 = 0.9960$; $C_{CAT\text{-}FITC} = 256.39\times(1\text{-}\exp(-6.51\times10^{-7}\times I_{FITC}))$, $R^2 = 0.9973$ $C_{\alpha\text{-}CT\text{-}FITC} = -402.83\times(1\text{-}\exp(2.21\times10^{-8}\times I_{FITC}))$, $R^2 = 0.9939$; $C_{Lys\text{-}FITC} = -21688.61\times(1\text{-}\exp(6.13\times10^{-10}\times I_{FITC}))$, $R^2 = 0.9962$. (C)Fluorescence spectrum I_{FITC} at 488 nm excitation of nanocapsules within FITC-labeled proteins. The encapsulation efficiencies of payload proteins were calculated using the Boxlucas 1 model equation in (B).

Table S2. The loading efficiencies of different proteins in nanocapsules. The mass concentration of proteins-FITC $C_{protein\text{-}FITC}$ was calculated from Boxlucas equation representing the relationship between the fluorescence intensity and concentration of FITC labeled proteins. The mass concentrations of the samples C_{NPs} were obtained from weighting after lyophilization.

	**BSA-FITC loaded nanocapsules**	**CAT-FITC loaded nanocapsules**	**α-CT-FITC loaded nanocapsules**	**Lys-FITC loaded nanocapsules**
**$C_{protein\text{-}FITC}$ [μg]**	**14.23**	**0.58**	**10.87**	**2.25**
**C_{NPs} [μg]**	**700**	**200**	**700**	**300**
**$C_{protein\text{-}FITC}$ /C_{NPs}**	**2.03%**	**0.29%**	**1.55%**	**0.75%**

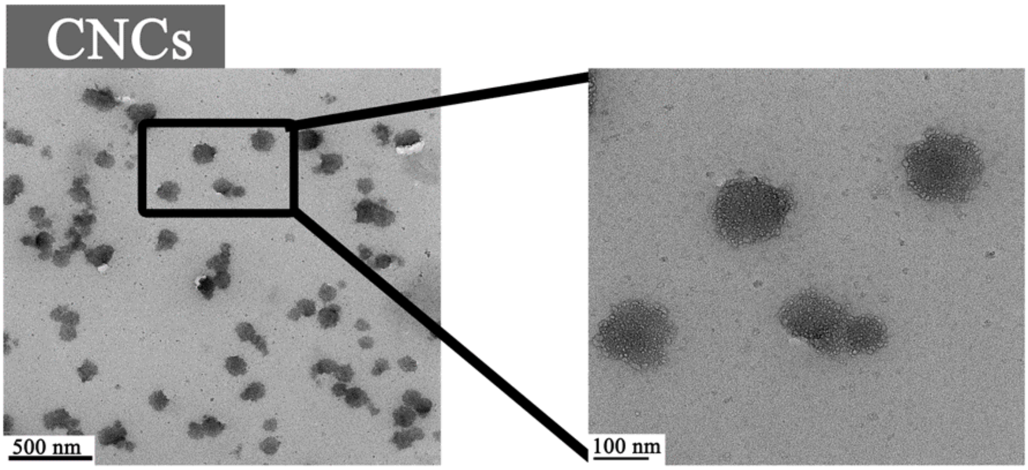

Fig. S5. TEM images of CNCs.

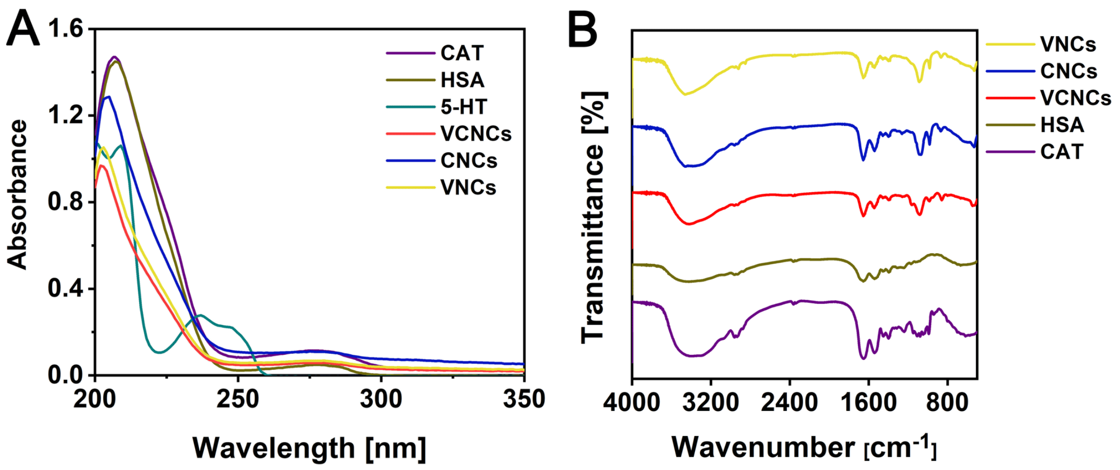

Fig. S6. (A) UV-vis spectra and (B) FTIR spectra of CAT, HSA, 5-HT, VCNCs, CNCs, and VNCs at a concentration 1mg/mL.

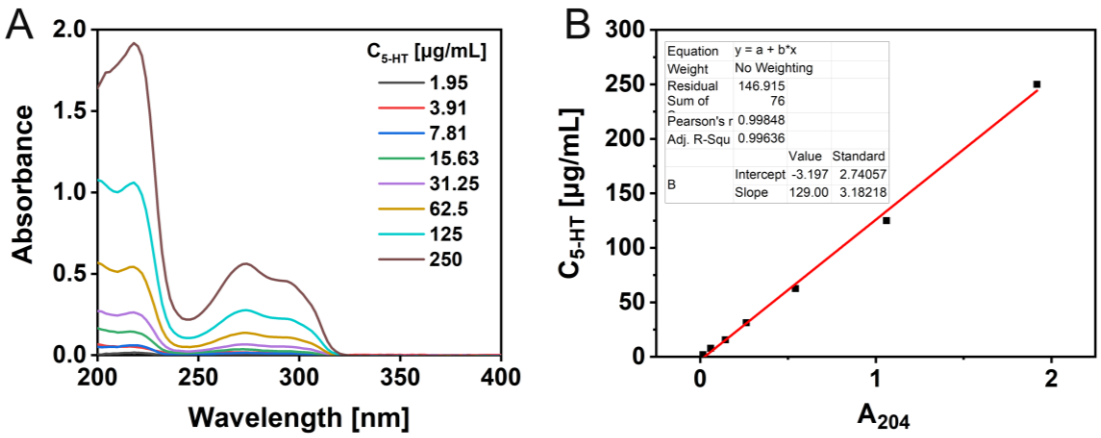

Fig. S7. (A)UV-vis spectra of 5-HT at different dilutions. (B) Linear fitting of the absorbance at 204 nm versus the concentration of 5-HT using the equation $C_{5\text{-}HT} = 129.00 \times A_{204} - 3.20$, $R^2 = 0.9964$.

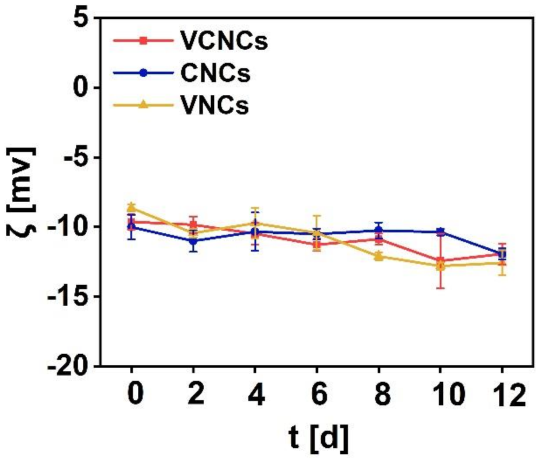

Fig. S8. The ζ-potential of VCNCs, CNCs, and VNCs in PBS at specific time points (C = 1 mg/mL).

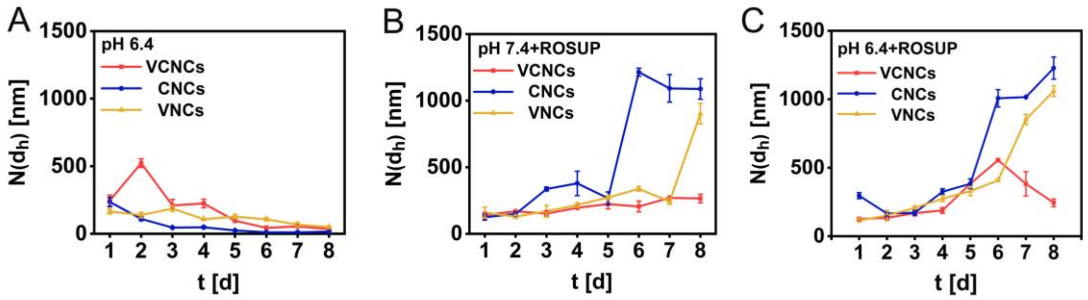

Figure S9. The mean hydrodynamic diameter $d_{h(N)}$ as derived from DLS measures of VCNCs, CNCs, and VNCs at C_{NPs} = 1 mg/mL dispersed in (A) pH 6.4 buffer (Citric acid-Na_2HPO_4), (B) pH 7.4+ROSUP buffer and (C) pH6.4+ROSUP at different time points.

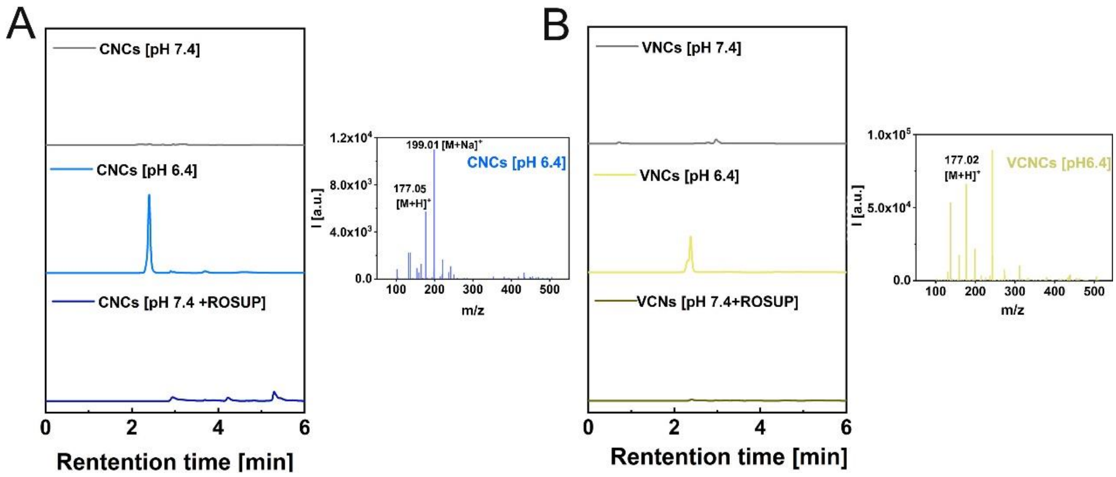

Fig. S10. CNCs and VNCs immersed in solutions of pH 7.4, pH 6.4, and pH 7.4+ROSUP at a concentration of $C_{5\text{-}HT}$ = 5000 μg/mL for 72 h. The samples were centrifuged with an ultracentrifuge filter (cutoff Mw = 3 kDa), and the eluent was collected for analysis by HPLC spectrum. The collected samples with retention times from 2 min to 4 min were further characterized by ESI-MS.

The concentration of 5-HT at different concentrations was determined by HPLC. The linear correlation between the peak area from HPLC spectra and 5-HT concentration was used to construct a standard curve.

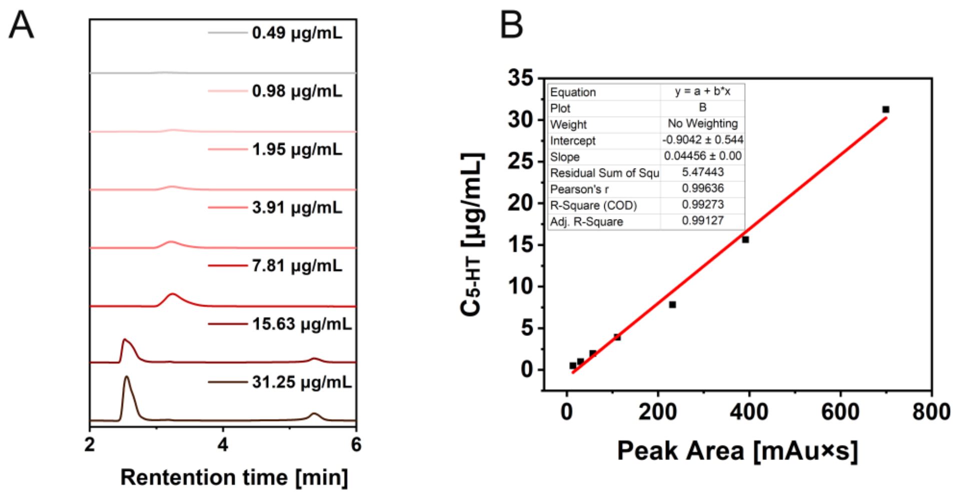

Fig. S11. Linear fitting of the peak area of 5-HT determined by HPLC versus the 5-HT concentration ($C_{5\text{-HT}}$:31.25 -0.49 μg/mL) as determined by weight. The results are $C_{5\text{-HT}}$ [μg/mL] = 0.045×peak area-0.90 , R^2 = 0.9913.

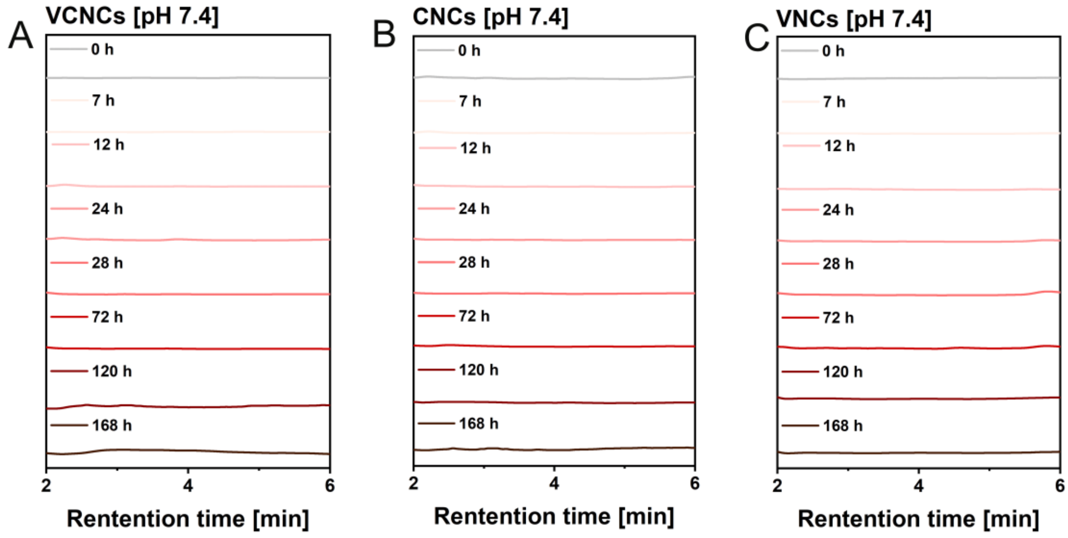

Fig. S12. (A)VCNCs, (B)CNCs, and (C)VNCs at a concentration $C_{5\text{-HT}}$ = 50 μg/mL incubated with pH 7.4 buffer for specific time points. The samples were centrifuged at 8000 rpm for 30 min with an ultracentrifuge filter (cutoff M_w = 3 kDa). The eluents were collected and quantified by HPLC.

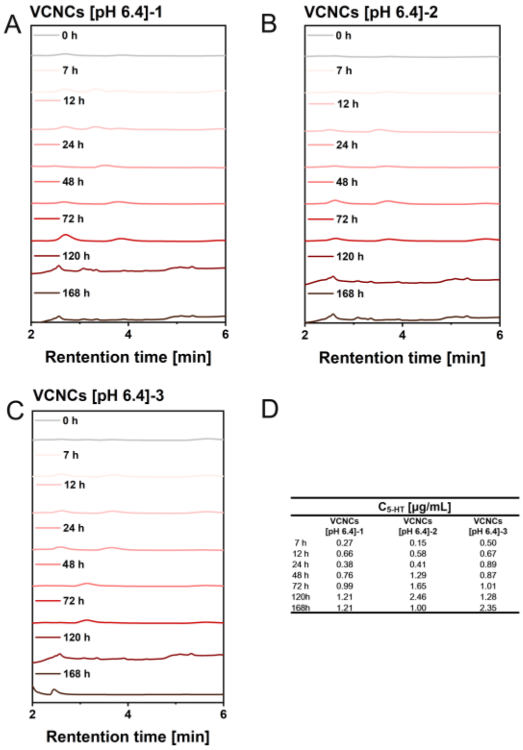

	C_{5-HT} [μg/mL]		
	VCNCs [pH 6.4]-1	VCNCs [pH 6.4]-2	VCNCs [pH 6.4]-3
7 h	0.27	0.15	0.50
12 h	0.66	0.58	0.67
24 h	0.38	0.41	0.89
48 h	0.76	1.29	0.87
72 h	0.99	1.65	1.01
120h	1.21	2.46	1.28
168h	1.21	1.00	2.35

Fig. S13. Compilation of data from Fig. 2C. VCNCs at a concentration of $C_{5\text{-}HT}$ = 50 μg/mL were incubated with pH 6.4 buffer for specific time points. The samples were centrifuged at 8000 rpm for 30 min with an ultracentrifuge filter (cutoff Mw = 3 kDa). The eluents were collected and quantified by HPLC. The results were represented by three independent samples, and the amounts of released 5-HT were quantified through peak area at t = 2-4 min according to the standard curve shown in Figure S11.

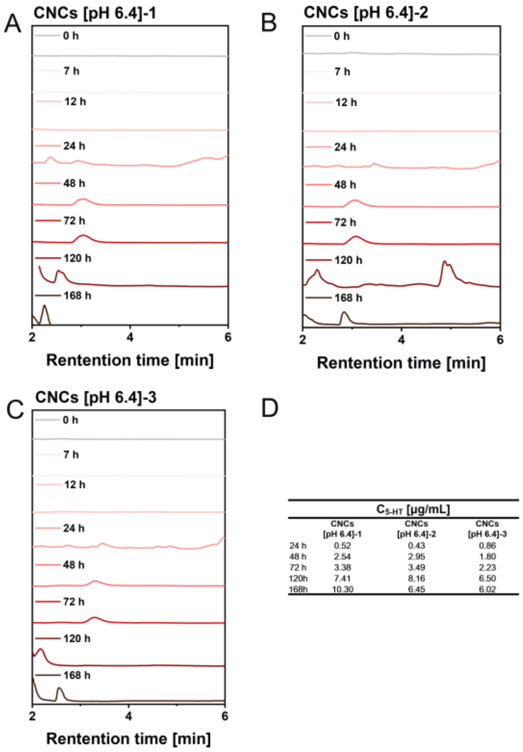

	C_{5-HT} [μg/mL]		
	CNCs [pH 6.4]-1	CNCs [pH 6.4]-2	CNCs [pH 6.4]-3
24 h	0.52	0.43	0.86
48 h	2.54	2.95	1.80
72 h	3.38	3.49	2.23
120h	7.41	8.16	6.50
168h	10.30	6.45	6.02

Fig. S14. Compilation of data from Fig. 2C CNCs at a concentration of $C_{5\text{-}HT}$ = 50 μg/mL were incubated with pH 6.4 buffer for specific time points. The samples were centrifuged at 8000 rpm for 30 min with an ultracentrifuge filter (cutoff Mw = 3 kDa). The eluents were collected and quantified by HPLC. The results were represented by three independent samples, and the amounts of released 5-HT were quantified through peak area at t = 2-4 min according to the standard curve shown in Figure S11.

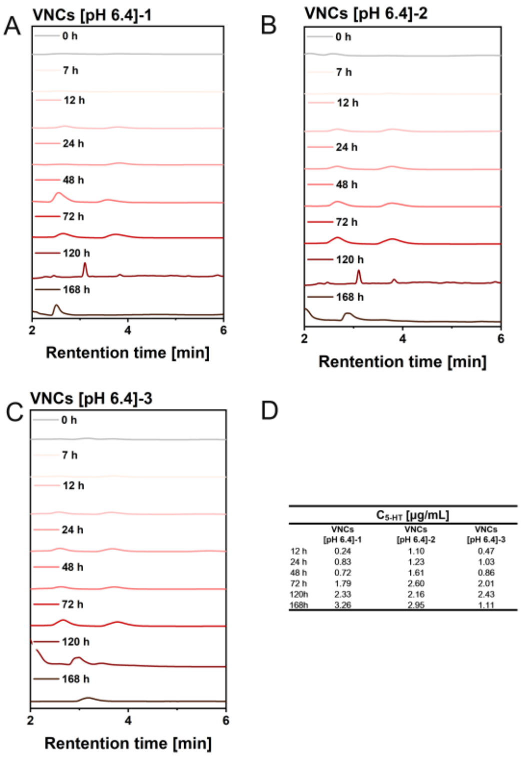

	$C_{5\text{-}HT}$ [μg/mL]		
	VNCs [pH 6.4]-1	VNCs [pH 6.4]-2	VNCs [pH 6.4]-3
12 h	0.24	1.10	0.47
24 h	0.83	1.23	1.03
48 h	0.72	1.61	0.86
72 h	1.79	2.60	2.01
120h	2.33	2.16	2.43
168h	3.26	2.95	1.11

Fig. S15. Compilation of data from Fig. 2C. VNCs at a concentration of $C_{5\text{-}HT}$ = 50 μg/mL were incubated with pH 6.4 buffer for specific time points. The samples were centrifuged at 8000 rpm for 30 min with an ultracentrifuge filter (cutoff Mw = 3 kDa). The eluents were collected and quantified by HPLC. The results were represented by three independent samples, and the amounts of released 5-HT were quantified through peak area at t = 2-4 min according to the standard curve shown in Figure S11.

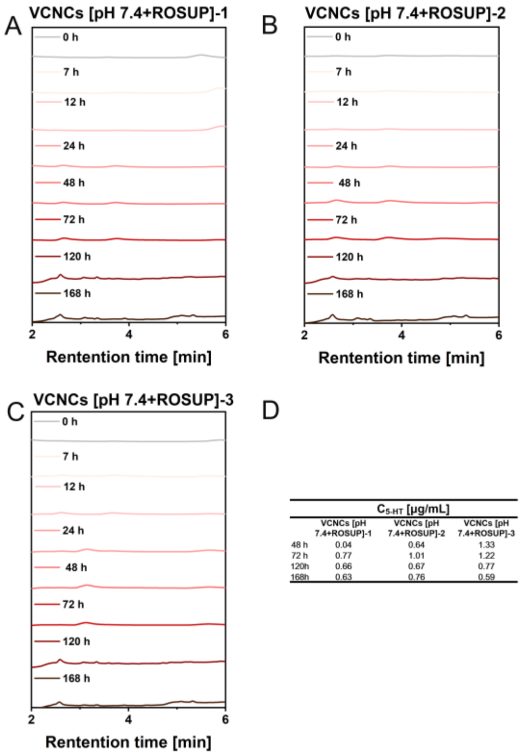

	$C_{5\text{-}HT}$ [μg/mL]		
	VCNCs [pH 7.4+ROSUP]-1	VCNCs [pH 7.4+ROSUP]-2	VCNCs [pH 7.4+ROSUP]-3
48 h	0.04	0.64	1.33
72 h	0.77	1.01	1.22
120h	0.66	0.67	0.77
168h	0.63	0.76	0.59

Fig. S16. Compilation of data from Fig. 2C VCNCs at a concentration of $C_{5\text{-}HT}$ = 50 μg/mL were incubated with pH 7.4+ROSUP buffer for specific time points. The samples were centrifuged at 8000 rpm for 30 min with an ultracentrifuge filter (cutoff Mw = 3 kDa). The eluents were collected and quantified by HPLC. The results were represented by three independent samples, and the amounts of released 5-HT were quantified through peak area at t = 2-4 min according to the standard curve shown in Figure S11.

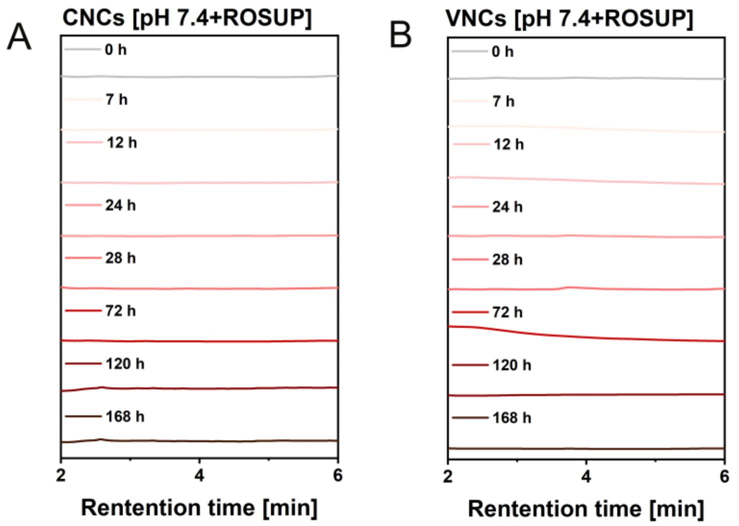

Fig. S17. CNCs and VNCs at a concentration $C_{5\text{-}HT}$ = 50 μg/mL incubated with pH 7.4+ROSUP buffer for specific time points. The samples were centrifuged at 8000 rpm for 30 min with an ultracentrifuge filter (cutoff M_w = 3 kDa). The eluents were collected and quantified by HPLC.

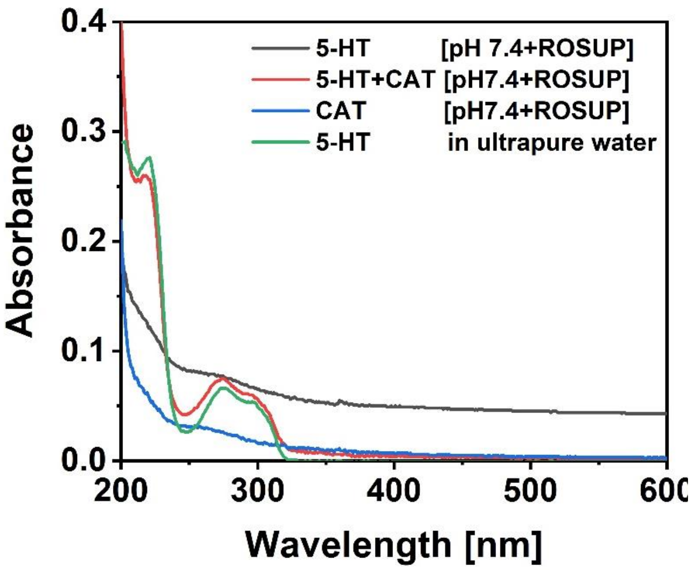

Fig. S18. UV-vis spectra of 5-HT in ultrapure water, 5-HT in pH 7.4+ROSUP buffer, CAT in pH 7.4+ROSUP buffer, 5-HT with CAT in pH 7.4+ROSUP buffer.

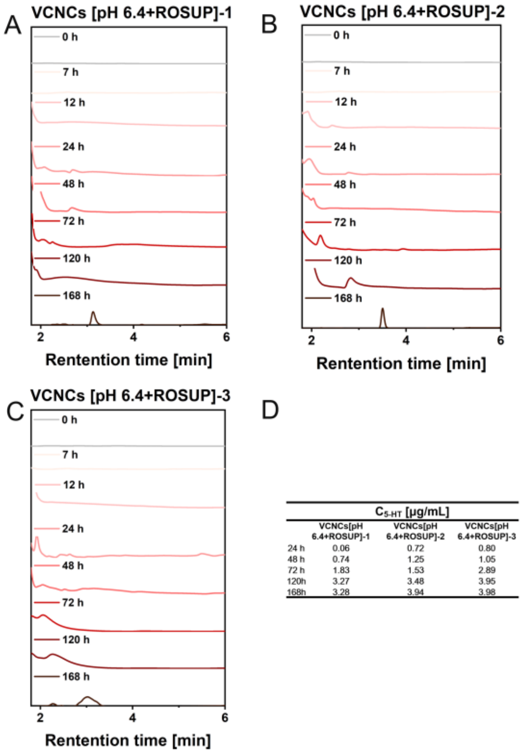

	$C_{5\text{-HT}}$ [μg/mL]		
	VCNCs[pH 6.4+ROSUP]-1	VCNCs[pH 6.4+ROSUP]-2	VCNCs[pH 6.4+ROSUP]-3
24 h	0.06	0.72	0.80
48 h	0.74	1.25	1.05
72 h	1.83	1.53	2.89
120h	3.27	3.48	3.95
168h	3.28	3.94	3.98

Fig. S19. Compilation of data from Fig. 2C. VCNCs at a concentration of $C_{5\text{-HT}}$ = 50 μg/mL were incubated with pH 6.4+ROSUP buffer for specific time points. The samples were centrifuged at 8000 rpm for 30 min with an ultracentrifuge filter (cutoff Mw = 3 kDa). The eluents were collected and quantified by HPLC. The results were represented by three independent samples, and the amounts of released 5-HT were quantified through peak area at t = 2-4 min according to the standard curve shown in Figure S11.

Table S3. The concentrations of released 5-HT over time was normalized to the initial 5-HT concentration (50 μg/mL) in the nanocapsules and plotted as $C_{5\text{-HT}}/C_{5\text{-HT(NPs)}}$.

t [h]	$C_{5\text{-HT}}$ [%]			
	VCNCs [pH 6.4]-1	VCNCs [pH 6.4]-2	VCNCs [pH 6.4]-3	VCNCs [pH 6.4]
7 h	0.54	0.30	1.01	0.62±0.36
12 h	1.32	1.16	1.34	1.28±0.10
24 h	0.77	0.83	1.79	1.13±0.57
48 h	1.52	2.58	1.74	1.94±0.56
72 h	1.99	3.31	2.01	2.43±0.75
120h	2.42	4.91	2.55	3.29±1.40
168h	2.43	1.99	4.70	3.04±1.46
	CNCs [pH 6.4]-1	CNCs [pH 6.4]-2	CNCs [pH 6.4]-3	CNCs [pH 6.4]
24 h	1.03	0.86	1.72	1.20±0.46
48 h	5.08	5.89	3.60	4.86±1.17
72 h	6.76	6.98	4.46	6.07±1.40
120h	14.82	16.33	13.00	14.72±1.67
168h	20.59	12.90	12.05	15.18±4.71
	VNCs [pH 6.4]-1	VNCs [pH 6.4]-2	VNCs [pH 6.4]-3	VNCs [pH 6.4]
12 h	0.47	2.19	0.93	1.20±0.89
24 h	1.66	2.47	2.06	2.06±0.40
48 h	1.44	3.23	1.72	2.13±0.97
72 h	3.57	5.20	4.01	4.26±0.84
120h	4.67	4.31	4.86	4.61±0.28
168h	6.51	5.90	2.22	4.88±2.32
	VNCs [pH 7.4+ROSUP]-1	VNCs [pH 7.4+ROSUP]-2	VNCs [pH 7.4+ROSUP]-3	VNCs [pH 7.4+ROSUP]
48 h	0.082726	1.284064	2.660077	1.34±1.29
72 h	1.547859	2.029998	2.431038	2.00±0.44
120h	1.323277	1.337536	1.532709	1.40±0.12
168h	1.258219	1.513994	1.179794	1.32±0.17
	VNCs [pH 6.4+ROSUP]-1	VNCs [pH 6.4+ROSUP]-2	VNCs [pH 6.4+ROSUP]-3	VNCs [pH 6.4+ROSUP]
24 h	0.12	1.44	1.61	1.06±0.81
48 h	1.48	2.50	2.09	2.02±0.51
72 h	3.67	3.07	5.78	4.17±1.43
120 h	6.53	6.95	7.91	7.13±0.70
168 h	6.57	7.88	7.95	7.47±0.78

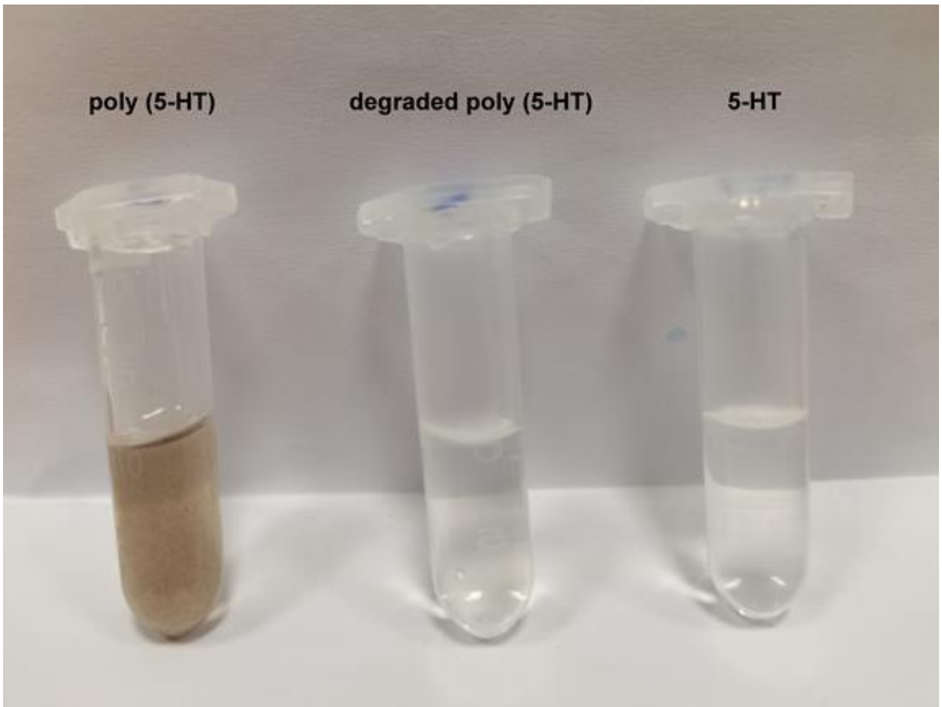

Fig. S20. Digital photos of aqueous poly(5-HT), degraded poly (5-HT), and 5-HT solution at the concentration of 2 mg/mL.

Although poly(5-HT) exhibited poor solubility in water, the presence of proteins within nanocapsules synthesized through 5-HT polymerization significantly enhanced water solubility. Consequently, the resulting nanocapsules demonstrated improved solubility characteristics.

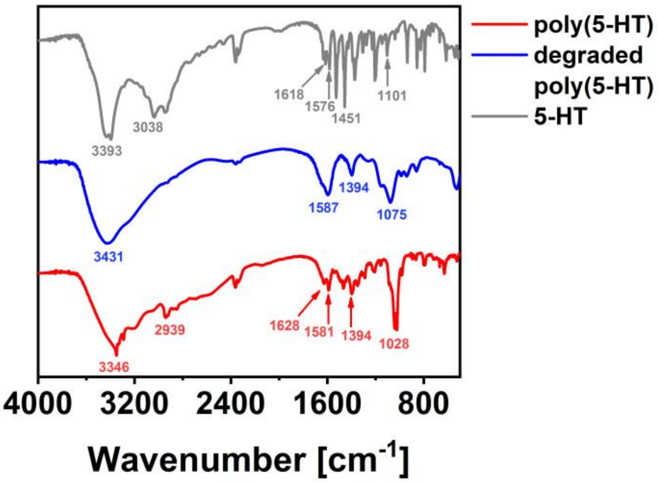

Fig. S21. FTIR spectra of poly(5-HT), degraded poly(5-HT), and 5-HT.

Featured band at ~ 3400 cm^{-1} that is assigned to ν(N-H) stretching, indicating the presence of primary amine group. The aliphatic ν(C-H) stretching at ~ 3000 cm^{-1} and two features at ~ 1600 cm^{-1} and ~ 1500 cm^{-1} assigned to ν_{ring} (C=C) confirmed the presence of benzene ring in 5-HT and poly(5-HT). The δ(O-H) bending at ~ 1500 cm^{-1} and ν(C-O) stretching at 1100-1000 cm^{-1} indicated the presence of phenolic hydroxyl groups in three samples. The peak at 1587 cm^{-1} in degraded poly(5-HT) is assigned to δ(N-H) bending.

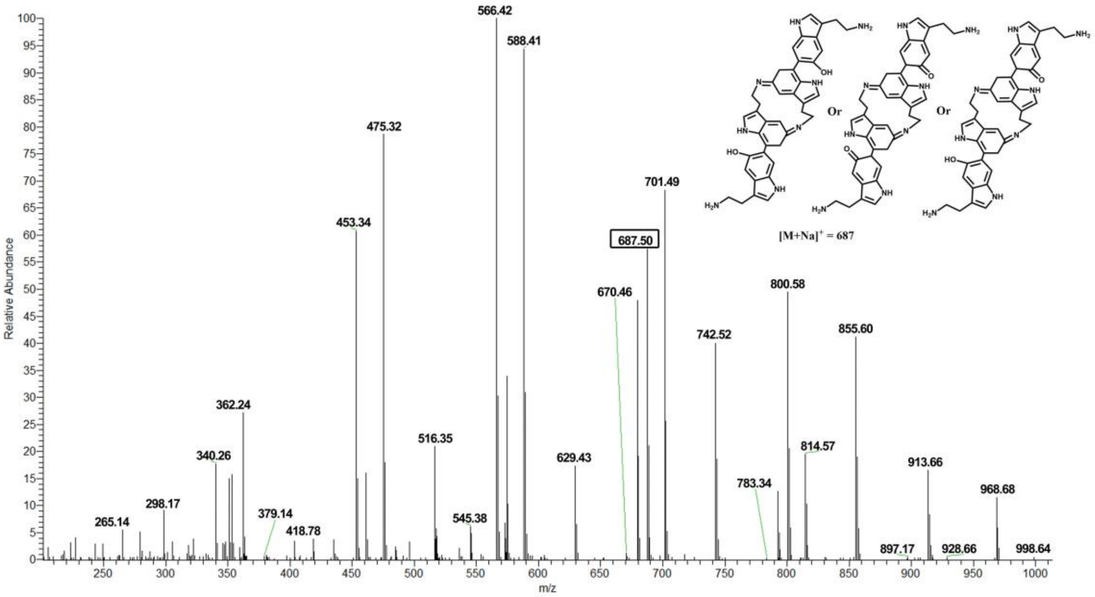

Fig. S22. Mass spectrum of the clear supernatant obtained from 5-HT polymerization at pH 9.5 (Tris-HCl buffer). The possible subunit can be distinguished from the data.

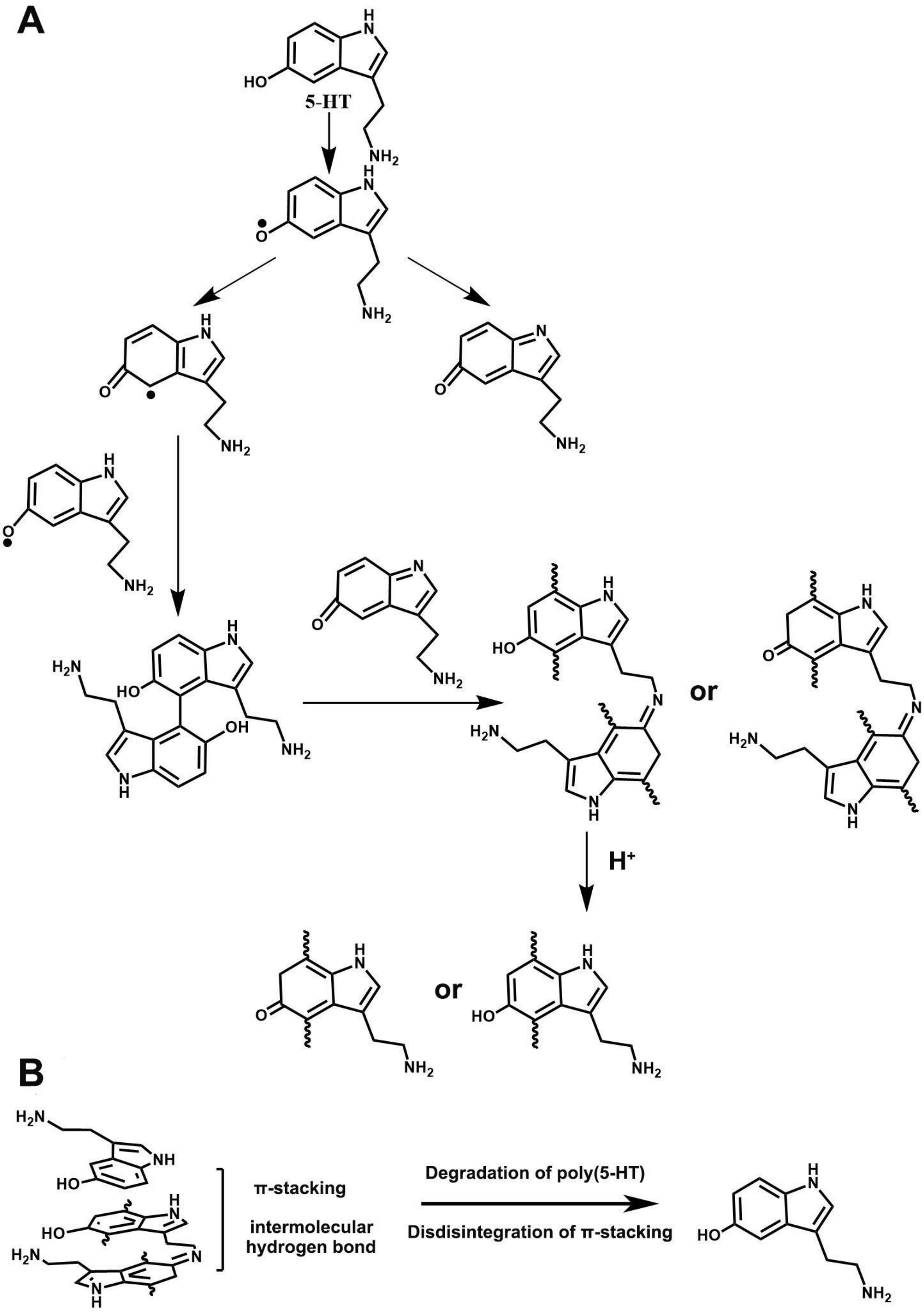

Fig. S23. The two proposed mechanisms of degradation of poly(5-HT).

Table S4. Ranges of concentrations of different samples for cytotoxicity assays.

	VCNCs ($C_{5\text{-}HT}$[μg/mL])	CNCs ($C_{5\text{-}HT}$[μg/mL])	VNCs ($C_{5\text{-}HT}$[μg/mL])	5-HT ($C_{5\text{-}HT}$[μg/mL])	CAT (C_{CAT}[μg/mL])	HSA (C_{HSA}[μg/mL])
1#	50	50	50	50	8	600
2#	25	25	25	25	4	300
3#	12.50	12.50	12.50	12.50	2	150
4#	6.25	6.25	6.25	6.25	1	75
5#	3.13	3.13	3.13	3.13	0.50	37.50
6#	1.56	1.56	1.56	1.56	0.25	18.75

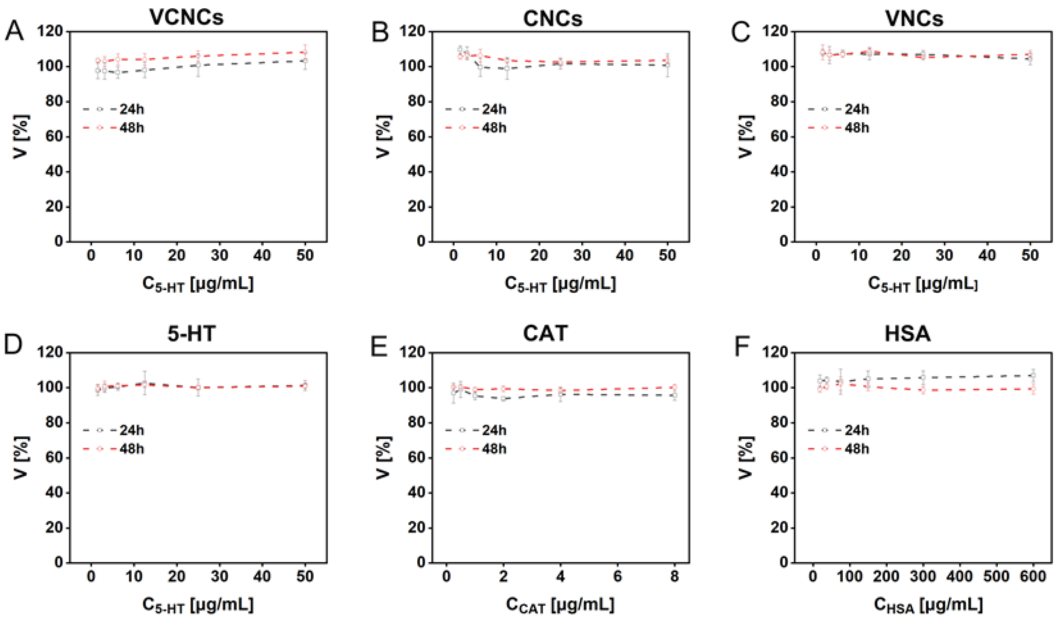

Fig. S24. Cell viability V studies of PC-12 cells exposed to (A)VCNCs, (B) CNCs, and (C)VNCs, (D)5-HT, (E)CAT, and (F) HSA at various concentrations (provided in Table S4). PC-12 cells were incubated with test samples in the cell medium for 24 h and 48 h. Cell viability was assessed using the resazurin assay and presented as the mean ± standard deviation of n≥3 measurements.

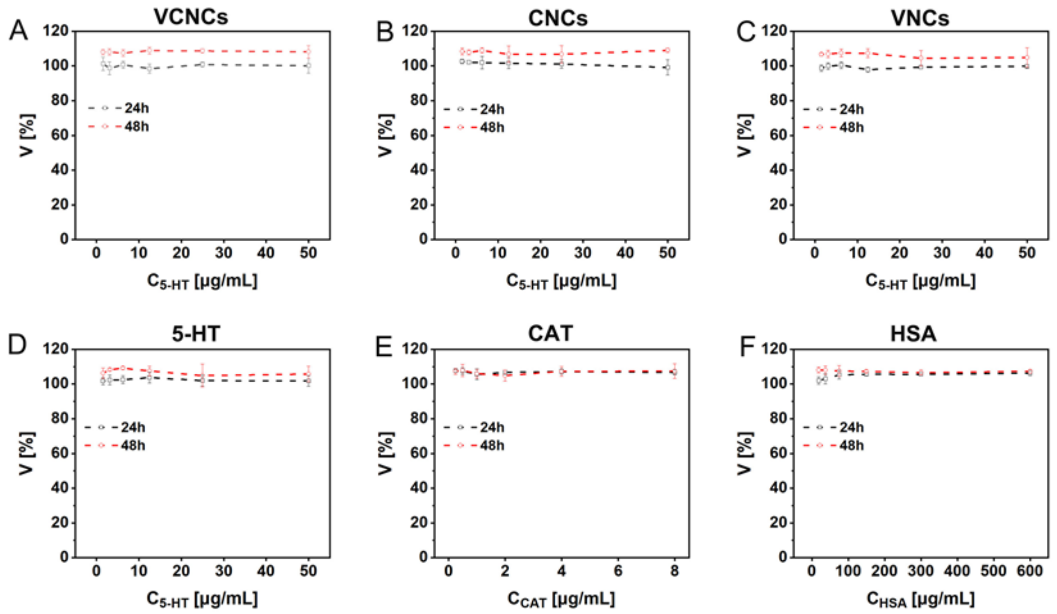

Fig. S25. Cell viability V studies of bEnd.3 cells exposed to (A)VCNCs, (B) CNCs, and (C)VNCs, (D)5-HT, (E)CAT, and (F) HSA at various concentrations (provided in Table S4). bEnd.3 cells were incubated with test samples in the cell medium for 24 h and 48 h. Cell viability was assessed using the resazurin assay and presented as the mean ± standard deviation of n≥3 measurements.

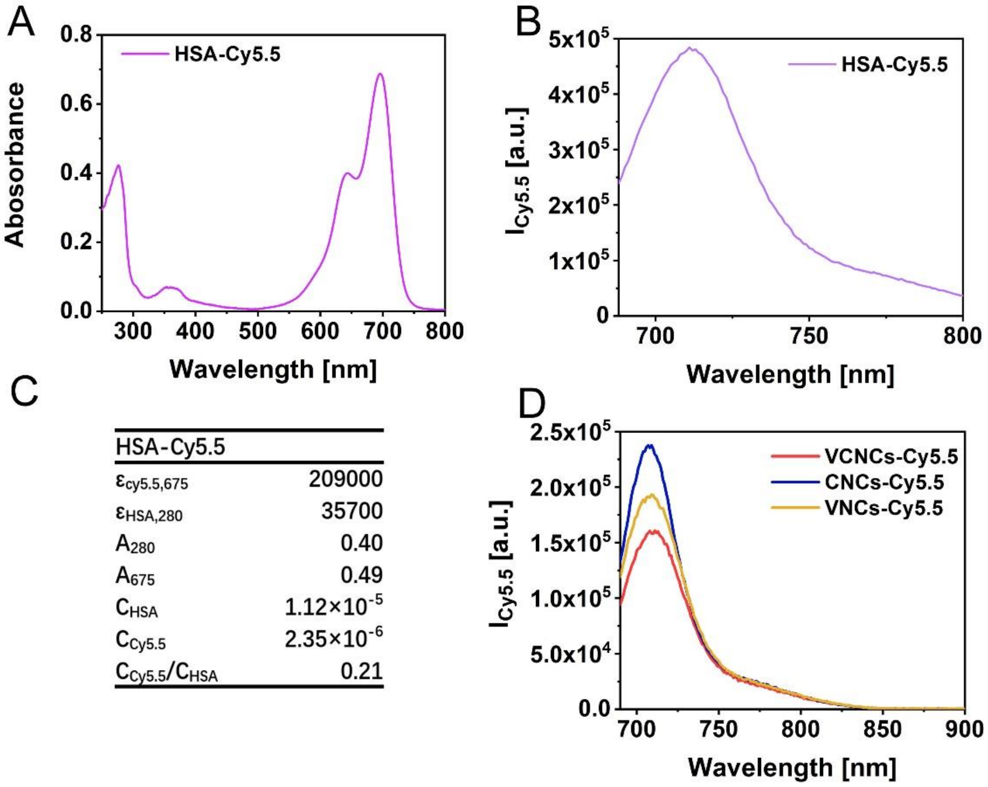

HSA-Cy5.5	
$\varepsilon_{cy5.5,675}$	209000
$\varepsilon_{HSA,280}$	35700
A_{280}	0.40
A_{675}	0.49
C_{HSA}	1.12×10^{-5}
$C_{Cy5.5}$	2.35×10^{-6}
$C_{Cy5.5}/C_{HSA}$	0.21

Fig. S26. (A)The UV-vis spectrum and (B)fluorescence spectrum $I_{Cy5.5}$ of HSA-Cy5.5. (C) The labeling efficiency of HSA-Cy5.5 was calculated by Beer-Lambert's law. (D)The fluorescence spectrum $I_{Cy5.5}$ at 678 nm excitation of VCNCs-Cy5.5, CNCs-Cy5.5, and VNCs-Cy5.5 at C_{NPs}=1mg/mL.

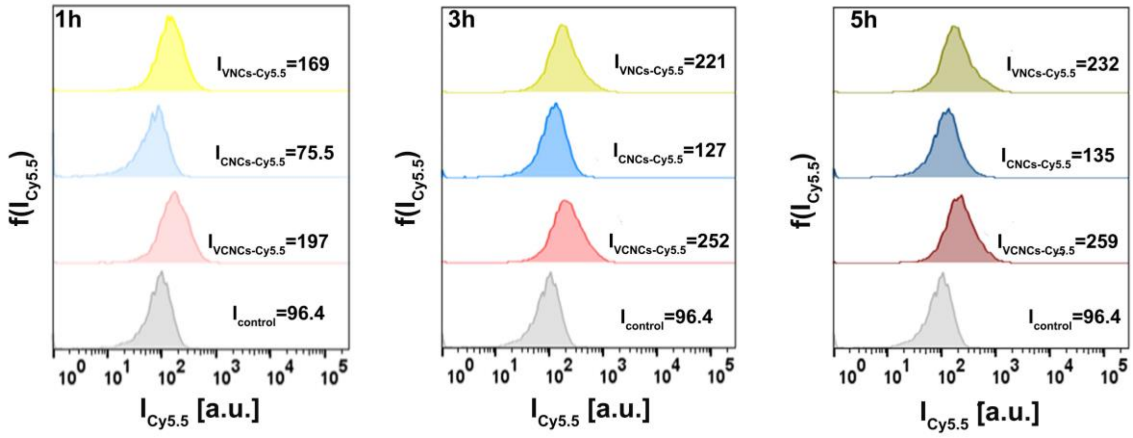

Fig. S27. Distributions of Cy5.5 fluorescence per cell $f(I_{Cy5.5})$ as measured by flow cytometry for bEnd.3 cells which had been exposed to VCNCs-Cy5.5, CNCs-Cy5.5, and VNCs-Cy5.5 at a concentration $C_{5\text{-}HT}$ = 25 μg/mL for 1, 3, and 5 h.

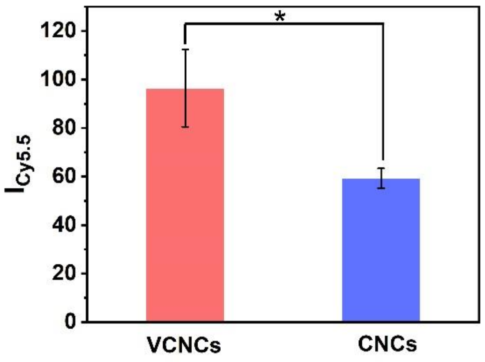

Fig. S28. Compilation of data from Fig. 3A, bEnd.3 cells in the transwell filter were exposed to VCNCs-Cy5.5 and CNCs-Cy5.5 at a concentration $C_{5\text{-}HT}$ = 25 μg/mL in cell medium for 3 h. For uptake study, mean Cy5.5. fluorescence $I_{Cy5.5}$ was determined by flow cytometry at t_{exp} = 3 h. Data are from n = 3 experiments, and significant differences between groups were analyzed using one-way ANOVA, * $P < 0.05$.

In the uptake studies, bEnd.3 cells were exposed to nanocapsules for a duration of t_{exp} = 3 h, resulting in a total cell count of N_{cell} ($t_{exp:3h}$). Subsequently, PC-12 cells were co-incubated with bEnd.3 cells for a further duration of t_{inc} = 24 h, resulting in a total cell count of N_{cell} ($t_{exp:3h}$ + $t_{inc:24h}$). The number of cells had increased by a factor of N_{cell} ($t_{exp:3h}$ + $t_{inc:24h}$)/ N_{cell} ($t_{exp:3h}$) from the initial cell count at $t_{exp:3h}$.

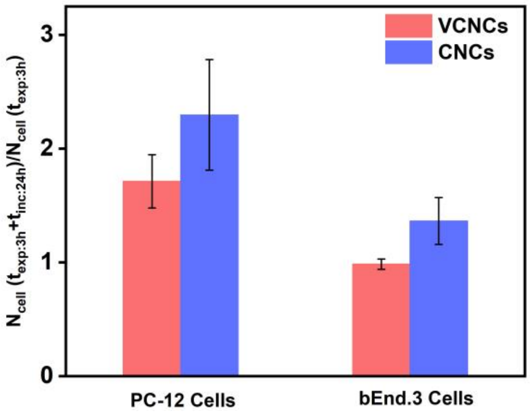

Fig. S29.The factor by which the number of cells N_{cell} had proliferated during t_{exp} + t_{inc} = 3 h + 24 h. The absolute cell numbers at t_{exp} = 3 h were 111.4±7.8 ×10^4 cells/mL (VCNCs) and 119.7 ± 18.8×10^4 cells/mL (CNCs). The factor N_{cell}($t_{exp:3h}$ + $t_{inc:24h}$)/ N_{cell}($t_{exp:3h}$) was calculated accordingly.

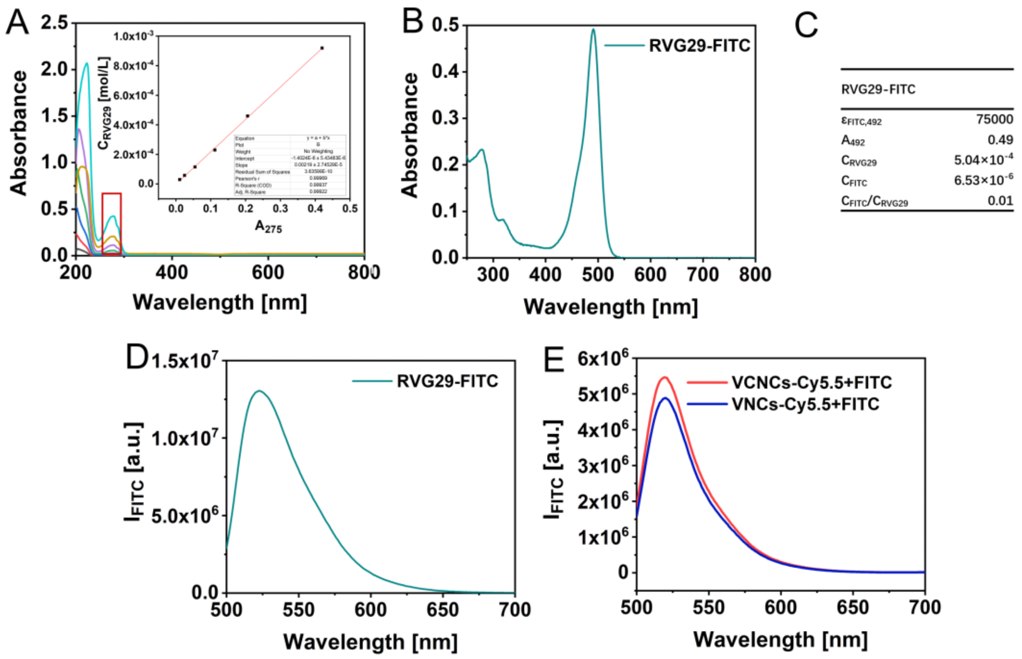

Fig. S30. The labeling efficiency of RVG29. (A) The RVG29 concentration was determined by the weight of the powder before dissolution versus the absorbance at 275 nm. Linear analysis was applied to the obtained data and fit results are shown as $C_{RVG29} = 2.19\times10^{-3}\times A_{276}-1.40\times10^{-6}$, $R^2 = 0.9992$. (B) The UV-vis spectrum of RVG29-FITC. (C) The concentration of RVG29 C_{RVG29} was calculated from the absorbance at 275 nm using the standard curve from the fit results in A. The concentration of FITC C_{FITC} was calculated using Beer-Lambert's law. The fluorescence spectrum I_{FITC} at 488 nm excitation of (D) free RVG29-FITC and (E) doubly labeled VCNCs-Cy5.5+FITC and VNCs-Cy5.5+FITC were also analyzed.

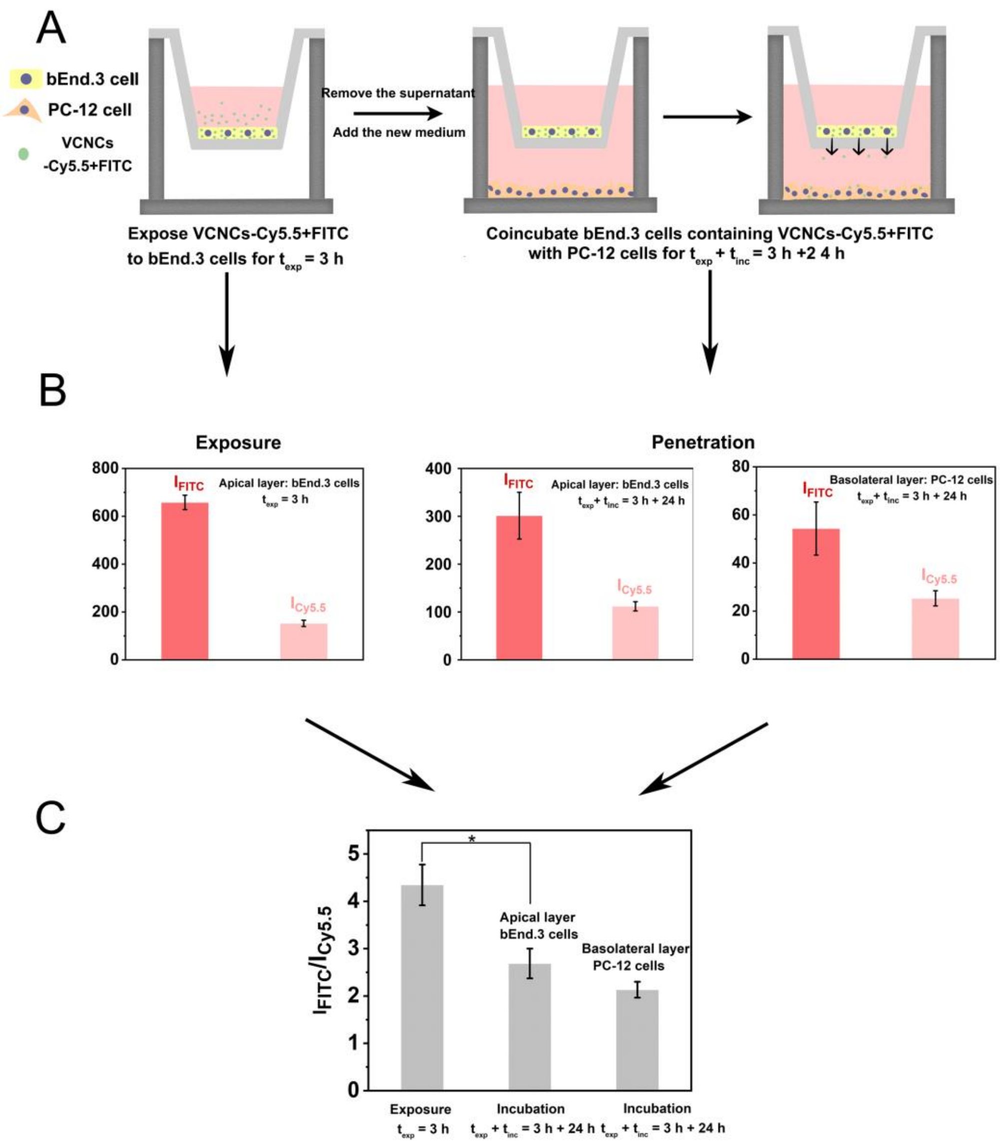

Fig. S31. (A)The schematic illustration of the in vitro BBB penetration model for studying the integrity of nanocapsules after passing through bEnd.3 cells. The doubly labeled VCNCs-Cy5.5+FITC was added to bEnd.3 cells in the transwell filter at a concentration of $C_{5\text{-HT}}$ = 25 μg/mL. After exposure for the time t_{exp} = 3 h, cellular uptake was characterized by flow cytometry. The mean Cy5.5 fluorescence $I_{Cy5.5}$ and mean FITC fluorescence I_{FITC} originating from each cell were background-corrected by control cells without nanocapsules. For the penetration study, the supernatant was replaced with fresh medium after exposure. bEnd.3 cells in the apical layer were then further incubated for the time t_{exp} + t_{inc} = 3 h + 24 h with PC-12 cells in the basolateral layer. (B) The mean Cy5.5 fluorescence $I_{Cy5.5}$ and mean FITC fluorescence I_{FITC} in bEnd.3 cells and PC-12 cells were analyzed by flow cytometry. (C) Ratios of fluorescence per cell originating from RVG29-FITC and HSA-Cy5.5 were determined from the data shown in B. Data correspond to the mean value and standard deviation of n = 3 measurements. One-way ANOVA was used to analyze significant differences between groups (* $P < 0.05$).

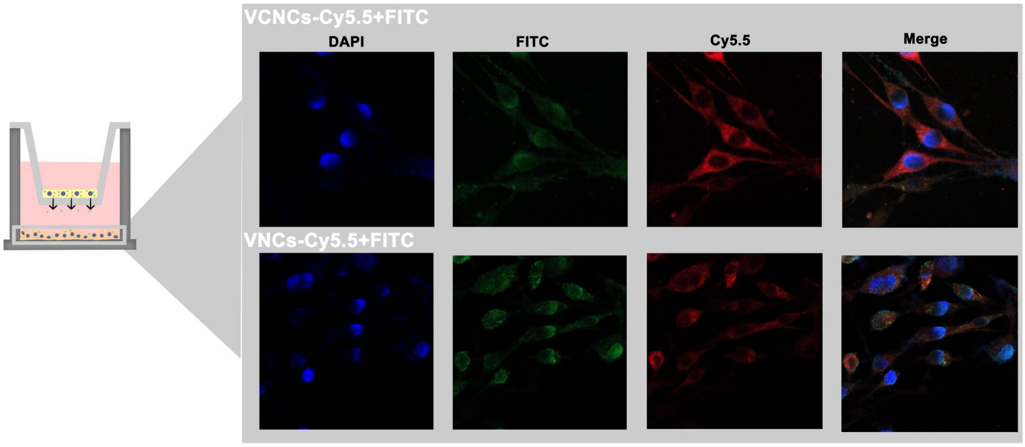

Fig. S32. Confocal images of PC-12 cells containing doubled labeled VCNCs-Cy5.5+FTIC and VNCs-Cy5.5+FITC at t_{exp} + t_{inc} = 3 h + 24 h (Cy5.5: red; FITC: green). Nuclei were stained with DAPI (blue).

Table S5. Concentration ranges of the different samples used for studying the effect on the cellular ROS level.

	VCNCs ($C_{5\text{-HT}}$[μg/mL])	CNCs ($C_{5\text{-HT}}$[μg/mL])	VNCs ($C_{5\text{-HT}}$[μg/mL])	5-HT ($C_{5\text{-HT}}$[μg/mL])	CAT (C_{CAT}[μg/mL])	HSA (C_{HSA}[μg/mL])
1#	50	50	50	50	4	300
2#	25	25	25	25	2	150
3#	12.50	12.50	12.50	12.50	1	75

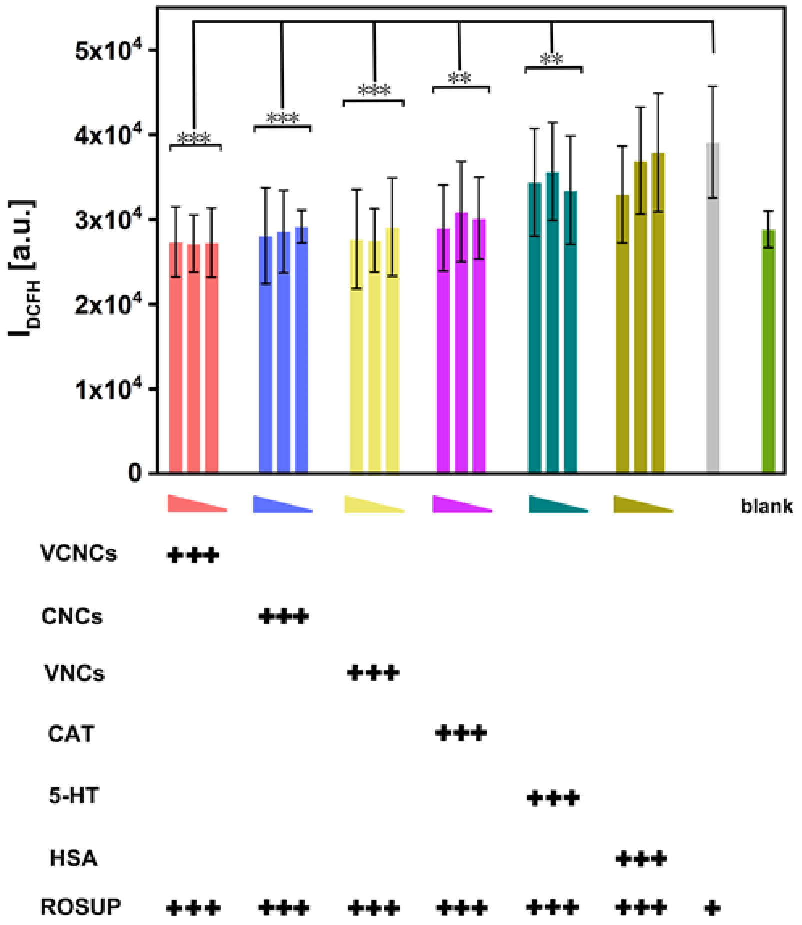

Fig. S33. Detection of DCFH fluorescence in cells after 3 h exposure to VCNCs, CNCs, and VNCs, 5-HT, CAT, and HSA at different concentrations. The legend “triangle” refers to samples exposed at higher concentrations to lower concentrations as indicated in Table S5. Fluorescence was detected by the microplate reader. Results are from three independent experiments. PC-12 cells incubated with DCFH-DA were used as the blank group. The significant differences between groups were analyzed by one-way ANOVA method, * *P < 0.01, * * * P < 0.001.

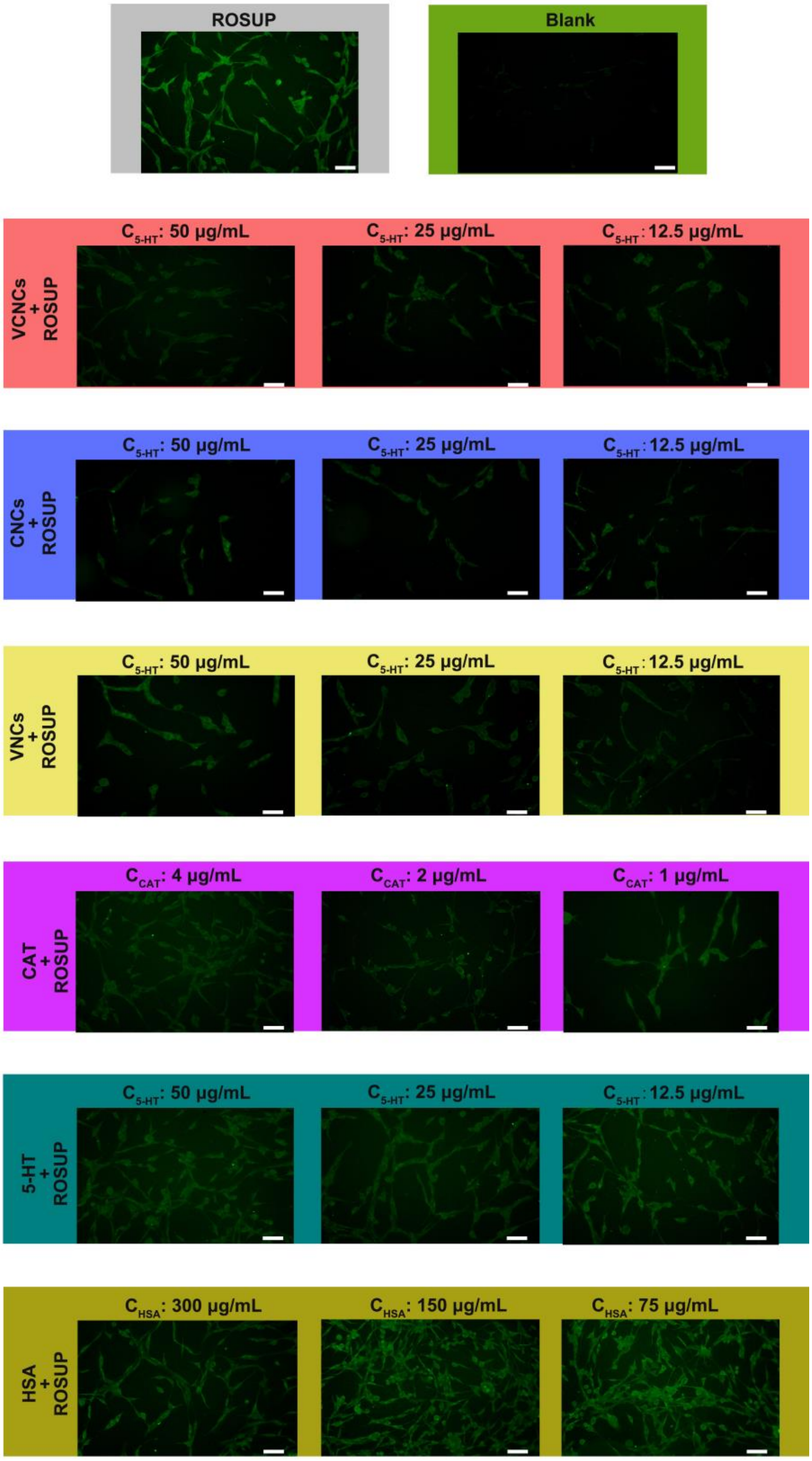

Fig. S34 Fluorescence images of intracellular ROS in PC-12 cells incubated with nanocapsules and reagents at different concentrations for 3 h. The scale bar represents 50 μm

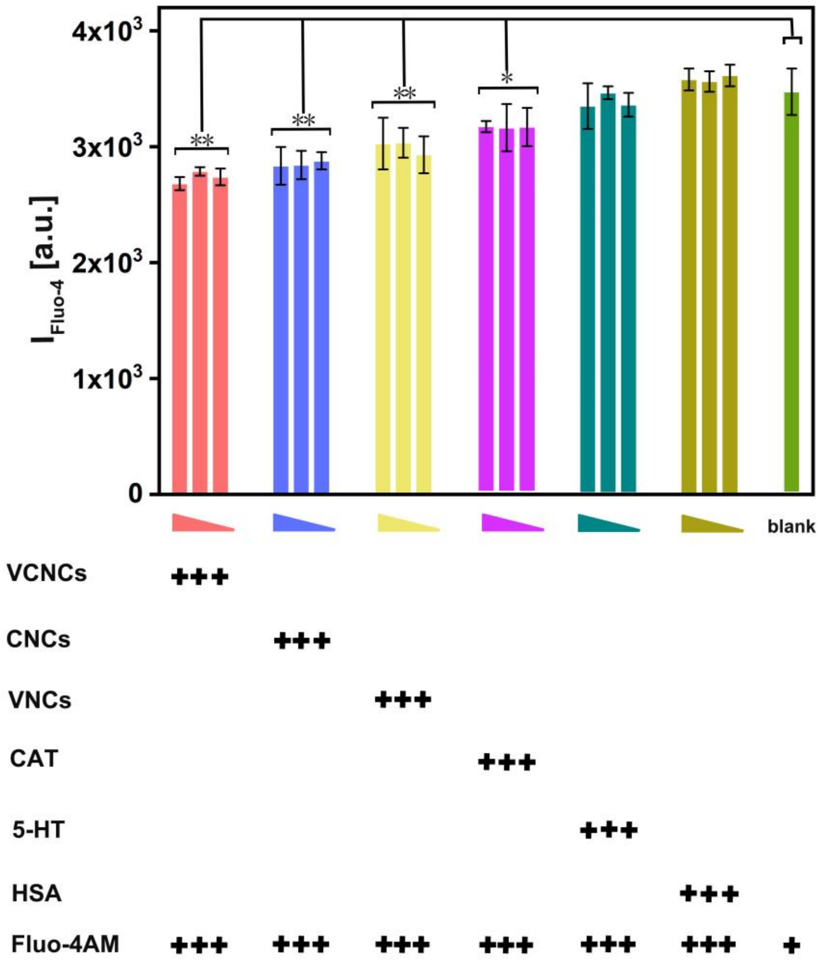

Fig. S35. Cellular fluorescence of Fluo-4 in cells incubated with nanocapsules and reagents for 3 h, recorded by a microplate reader at the FITC channel. The concentrations of nanocapsules and reagents are displayed in Table S5. The intensity of Fluo-4 fluorescence is proportional to the level of cytoplasmic calcium. PC-12 cells incubated with Fluo-4AM were used as the blank group. Results are from three independent experiments. The significant differences between groups were analyzed by the one-way ANOVA method, $^*P < 0.05$, $^{**}P < 0.01$.

Table S6. Mice were subjected to different stressors for 5 weeks.

	Day1	Day2	Day3	Day4	Day5	Day6	Day7
Week1	Wet bedding 24 h	Food deprivation 24 h	Tail pinch 1 min	Water deprivation 24 h	Cage tilting 2 h	Cold swim 3 min	Crowding 24 h
Week2	Restrain 2 h	Light cycle reversal	Dark cycle reversal	Food deprivation 24 h	Strobe light 2 h	Tail pinch 1 min	Cage tilting 2 h
Week3	Wet bedding 24 h	Water deprivation 24 h	Restrain 2 h	Crowding 24 h	Cold swim 3 min	Light cycle reversal	Dark cycle reversal
Week4	Strobe light 2 h	Food deprivation 24 h	Water deprivation 24 h	Cage tilting 2 h	Tail pinch 1 min	Cold swim 3 min	Crowding 24 h
Week5	Wet bedding 24 h	Restrain 2 h	Light cycle reversal	Dark cycle reversal	Strobe light 2 h	Food deprivation 24 h	Water deprivation 24 h

Table S7. The CUMS schedule during the treatment session.

	Day1	Day2	Day3	Day4	Day5	Day6	Day7
Week1	Wet bedding 24 h	Food deprivation 24 h	Water deprivation 24 h	Cage tilting 2 h	Restrain 2 h	Light cycle reversal	Crowding 24 h
Week2	Water deprivation 24 h	Wet bedding 24 h	Cage tilting 2 h	Food deprivation 24 h	Crowding 24 h	Restrain 2 h	Dark cycle reversal

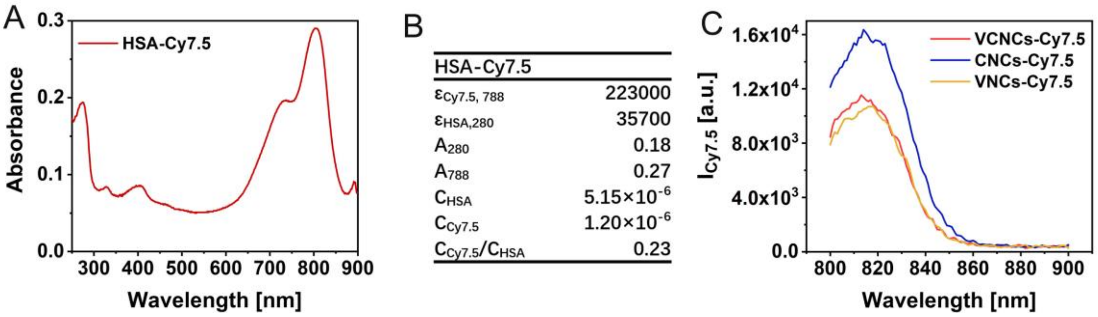

B

HSA-Cy7.5	
$\varepsilon_{Cy7.5,\ 788}$	223000
$\varepsilon_{HSA,280}$	35700
A_{280}	0.18
A_{788}	0.27
C_{HSA}	5.15×10^{-6}
$C_{Cy7.5}$	1.20×10^{-6}
$C_{Cy7.5}/C_{HSA}$	0.23

Fig. S36. (A) The UV-vis spectrum of HSA-Cy7.5. (B) The labeling efficiency of HSA-Cy7.5 was calculated by Beer-Lambert's law. (C)The fluorescence spectrum $I_{Cy7.5}$ at 788 nm excitation for VCNCs-Cy7.5, CNCs-Cy7.5, and VNCs-Cy7.5 at C_{NPs} = 1mg/mL.

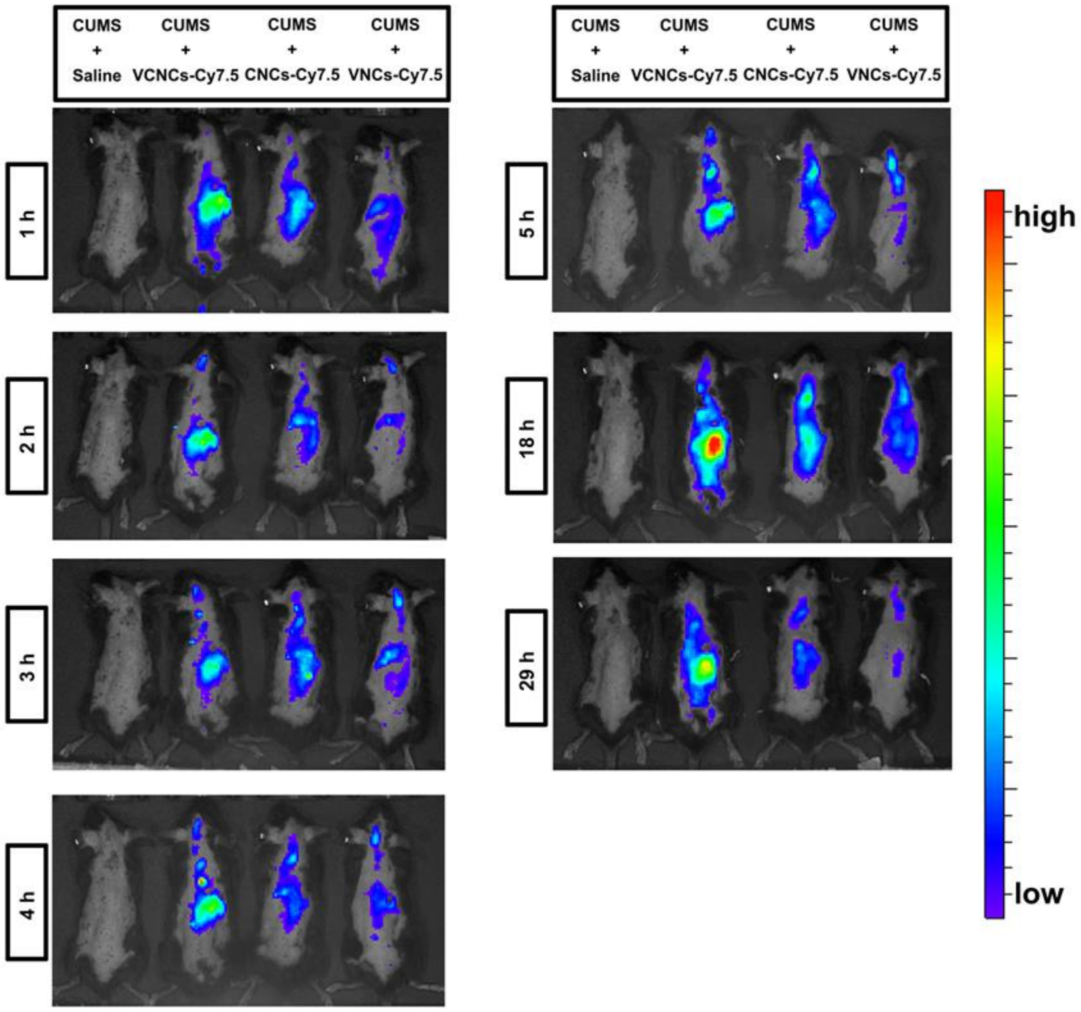

Fig. S37. *In vivo* Cy7.5 fluorescence of CUMS mice after intravenous injection with VCNCs-Cy7.5, CNCs-Cy7.5, and VNCs-Cy7.5 at a concentration $C_{5\text{-HT}}$:400 μg/kg for 3 h.

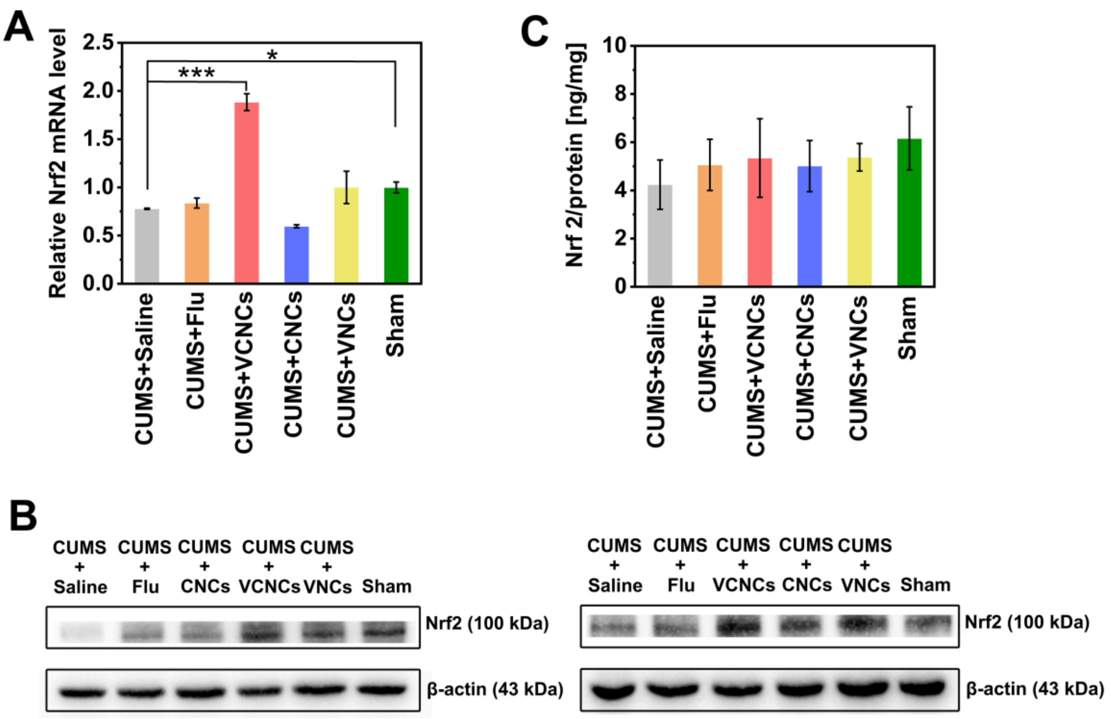

Fig. S38. (A) RT-qPCR analysis showing the relative mRNA levels of Nrf2 in the hippocampi of mice across all groups (n = 3). (B) Compilation of data from Fig. 5G. Western blot analysis illustrating the Nrf2 protein levels in hippocampus tissues subjected to different treatments. (C) ELISA detection of hippocampal Nrf2 levels following treatments, with normalization to the protein concentration of each sample (n = 3). The significant differences between groups were analyzed using the one-way ANOVA method, $*P < 0.05$, $**P < 0.01$, $***P < 0.001$.

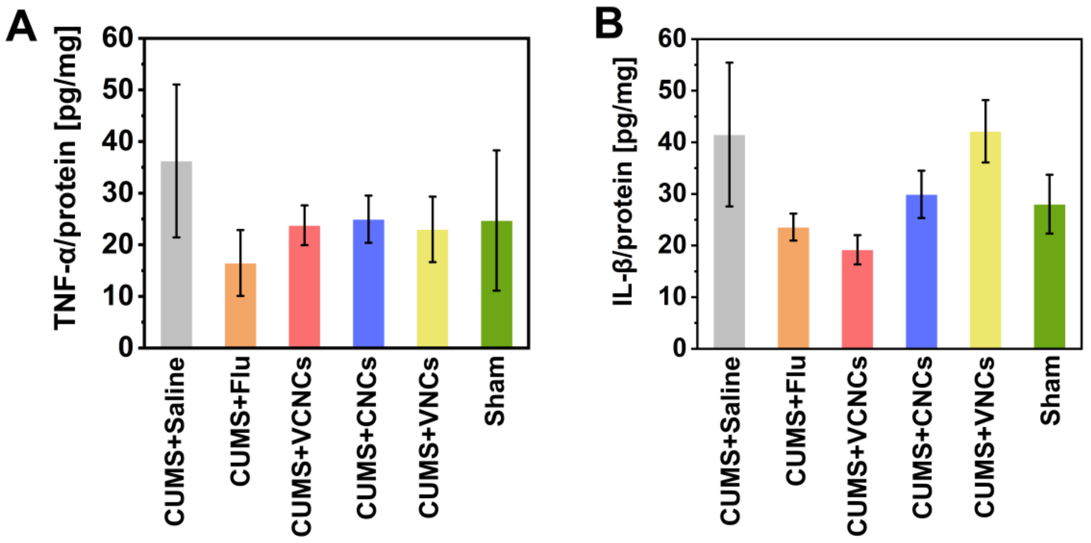

Fig. S39. The levels of (A) TNF-α and (B) IL-1β in the mice hippocampi after different antidepressive treatment were detected using ELISA kits. The results normalized by the protein concentration of each sample (n = 3).

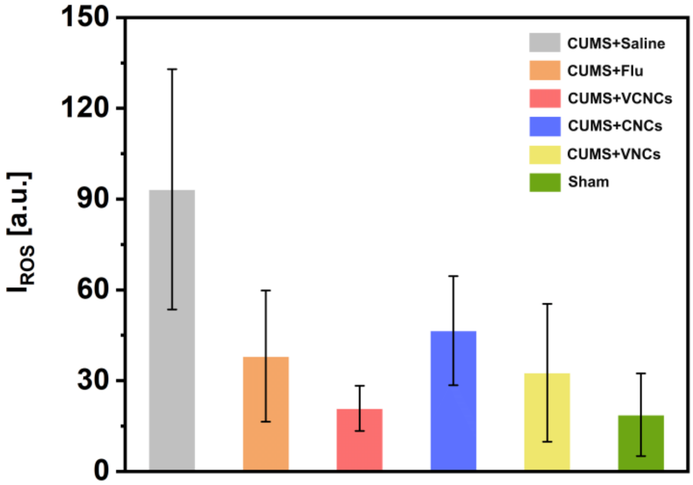

Fig. S40. Compilation of data from Fig. 5F. Quantitative evaluation of the ROS intensity in Fig. 5F analyzed by Image J.

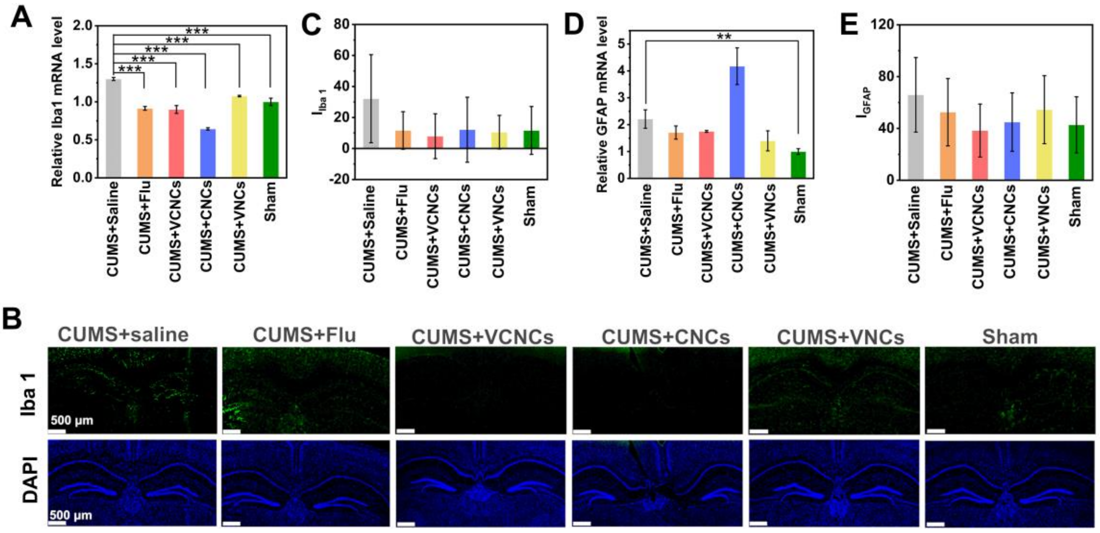

Fig. S41. RT-qPCR of relative mRNA levels of (A) Iba 1 and (D) GFAP in the hippocampi of mice in all groups (n = 3). (B) Iba 1/DAPI staining of the brain after different treatments. Quantitative evaluation of the (C) Iba 1 and (E) GFAP intensity in Fig. S41B and Fig. 5F respectively analyzed by Image J. The significant differences between groups were analyzed using the one-way ANOVA method, *P < 0.05, **P < 0.01, *** P < 0.001.

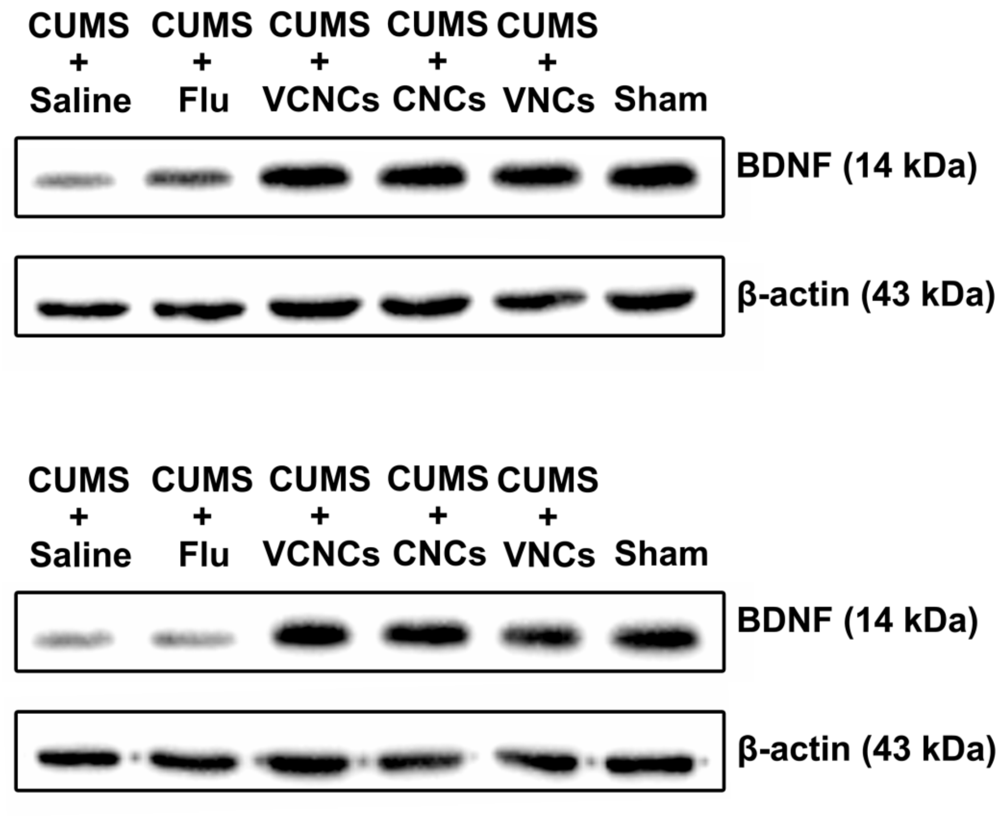

Fig. S42. Compilation of data from Fig. 5H. The other two replicates of western blot analysis for BDNF protein levels in hippocampus tissues subjected to different treatments.

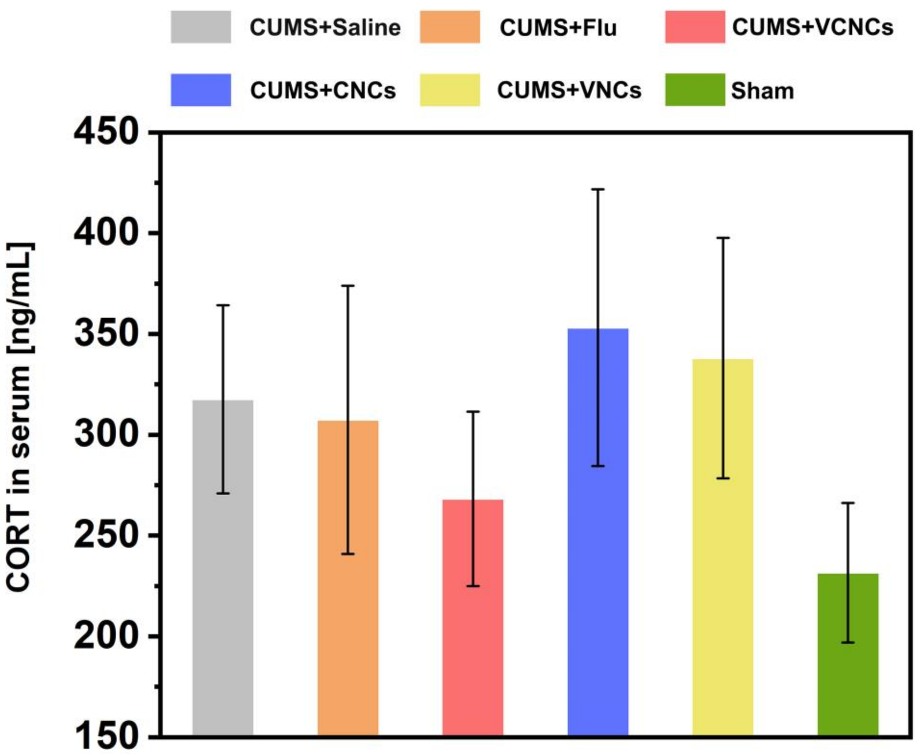

Fig. S43. The cortisol level of serum in the mice after different antidepressant treatments. The data are from three independent experiments.

Table S8. The blood indexes examination from the mice after the treatments. Data are presented as mean±s.d.(n=3)

	**Normal range**	**Saline**	**Fluoxetine**	**VCNCs**	**CNCs**	**VNCs**	**Sham**
**WBC**	**0.80-6.80 (10^9/L)**	**4.70±0.22**	**4.6±1.25**	**5.13±1.01**	**2.45±0.64**	**3.25±1.91**	**3.80±0.85**
**Gran#**	**0.10-1.80 (10^9/L)**	**1.18±0.17**	**0.97±0.23**	**1.33±0.64**	**0.55±0.21**	**0.40±0.28**	**0.70±0.28**
**RBC**	**6.36-9.42 (10^{12}/L)**	**8.89±0.55**	**8.18±0.19**	**8.48±0.16**	**7.93±1.64**	**8.20±0.25**	**9.41±0.04**
**HGB**	**110.00-143.00 (g/L)**	**143.25±13.30**	**143.33±2.08**	**135.00±7.55**	**139.00±2.83**	**131.00±12.73**	**154.50±6.36**
**MCH**	**15.80-19.00 (pg)**	**16.05±1.22**	**17.50±0.40**	**15.83±0.61**	**17.90±4.10**	**15.95±1.06**	**16.35±0.64**
**PLT**	**450.00-1590.00 (10^9/L)**	**574.75±141.19**	**723.67±126.47**	**534.333±11.02**	**853.50±649.83**	**567.50±144.96**	**507.50±12.02**

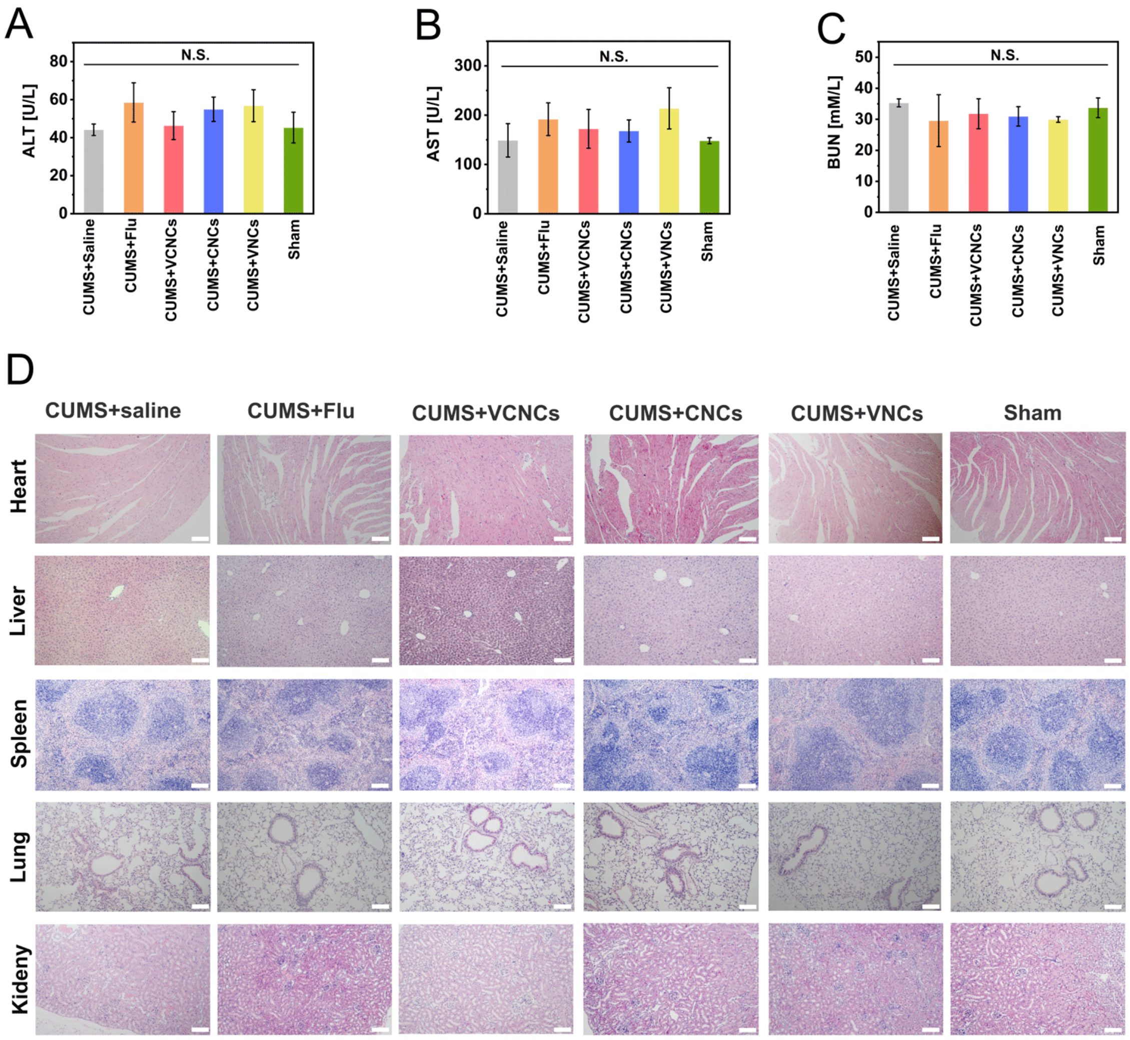

Fig. S44. Biosafety of the nanocapsules. Levels of liver and kidney biomarkers including (A) ALT, (B) AST, and (C) BUN in mice treated with nanocapsules (n = 3). The significant differences between groups were analyzed by one-way ANOVA method, where N.S. represents not statistically significant. (D) HE stained images of major tissues (heart, liver, spleen, lung, and kidney) in mice after antidepression treatments. The scale bar represents 100 μm.
